# Readily Accessible Strained Difunctionalized *trans*‐Cyclooctenes with Fast Click and Release Capabilities**

**DOI:** 10.1002/chem.202203375

**Published:** 2022-12-07

**Authors:** Daan Sondag, Luuk Maartense, Heleen de Jong, Frank F. J. de Kleijne, Kimberly M. Bonger, Dennis W. P. M. Löwik, Thomas J. Boltje, Jan Dommerholt, Paul B. White, Daniel Blanco‐Ania, Floris P. J. T. Rutjes

**Affiliations:** ^1^ Institute for Molecules and Materials Radboud University 6525 AJ Nijmegen Netherlands

**Keywords:** bioorthogonal conjugation, click and release, click chemistry, cycloaddition, TCO, tetrazine, *trans*-cyclooctene

## Abstract

The click reaction between a functionalized *trans*‐cyclooctene (TCO) and a tetrazine (Tz) is a compelling method for bioorthogonal conjugation in combination with payload releasing capabilities. However, the synthesis of difunctionalized TCOs remains challenging. As a result, these compounds are poorly accessible, which impedes the development of novel applications. In this work, the scalable and accessible synthesis of a new bioorthogonal difunctionalized TCO is reported in only four single selective high yielding steps starting from commercially available compounds. The TCO‐Tz click reaction was assessed and revealed excellent kinetic rates and subsequently payload release was shown with various functionalized derivatives. Tetrazine triggered release of carbonate and carbamate payloads was demonstrated up to 100 % release efficiency and local drug release was shown in a cellular toxicity study which revealed a >20‐fold increase in cytotoxicity.

## Graphical abstract

**The synthesis of a novel** strained difunctionalized *trans*‐cyclooctene in only four high‐yielding steps is presented and the releasing capabilities of functionalized derivatives were evaluated upon tetrazine activation. This method presents a major step forward in the ease and efficiency of synthesizing releasing difunctionalized *trans*‐cyclooctenes.

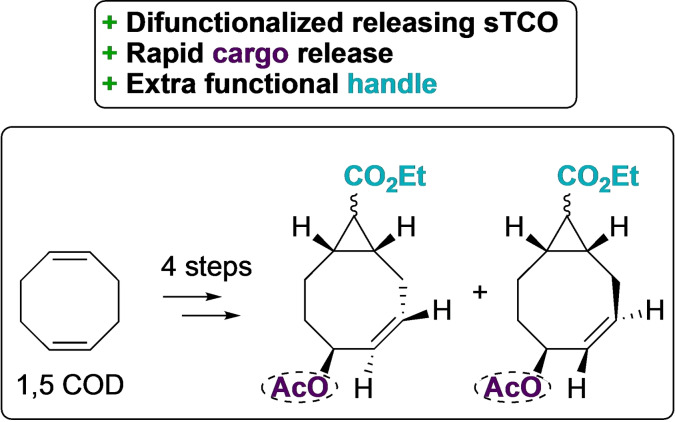

## Introduction

Ever since the visionary publication of the Sharpless group about click chemistry, this field has grown significantly and has had a tremendous impact on drug discovery, bioconjugation and modern chemistry in general.[^1^, ^2^] Many click reactions are bioorthogonal, a term first used by Bertozzi in 2003, indicating that functional groups are only mutually reactive and do not interfere with biochemical processes occurring in living systems.[^3^, ^4^, ^5^, ^6^] Examples of bioorthogonal click reactions include the strain‐promoted azide‐alkyne cycloaddition (SPAAC), the Staudinger ligation and the strain‐promoted oxidation‐controlled cyclooctyne‐1,2‐quinone cycloaddition (SPOQC).[^7^, ^8^, ^9^] In the last decade, bioorthogonal chemistry has successfully been employed in both diagnostic (e. g. radiolabeling)[^10^, ^11^] and therapeutic applications such as antibody‐drug conjugates (ADCs)^[12]^ and prodrugs.[^13^, ^14^, ^15^]

The inverse‐electron‐demand Diels‐Alder reaction (IEDDA), a reaction between an electron‐rich dienophile and an electron‐poor diene, has been applied for the ligation of *trans*‐cyclooctenes (TCOs) and tetrazines (Tz).^[16]^ This bioorthogonal ligation usually shows high reaction rates and makes in vivo bioconjugation possible at low concentrations, as demonstrated in numerous studies.[^17^, ^18^, ^19^, ^20^] As a next step, in 2013 Versteegen et al. reported on the design and synthesis of TCO derivatives that upon reaction with a tetrazine eliminate a substituent at the allylic position of the TCO. This so‐called click‐to‐release approach was applied to enable site‐specific and traceless drug release upon TCO‐tetrazine ligation.[^21^, ^22^] The fast reaction kinetics and click and release capability of the TCO‐tetrazine adduct opens up countless possibilities for novel applications in drug release and beyond.[^23^, ^24^] However, the complex synthesis of TCOs ‐ especially of the releasing ones ‐ prevents the roll‐out of this versatile technology towards novel applications.

**Figure 1:**
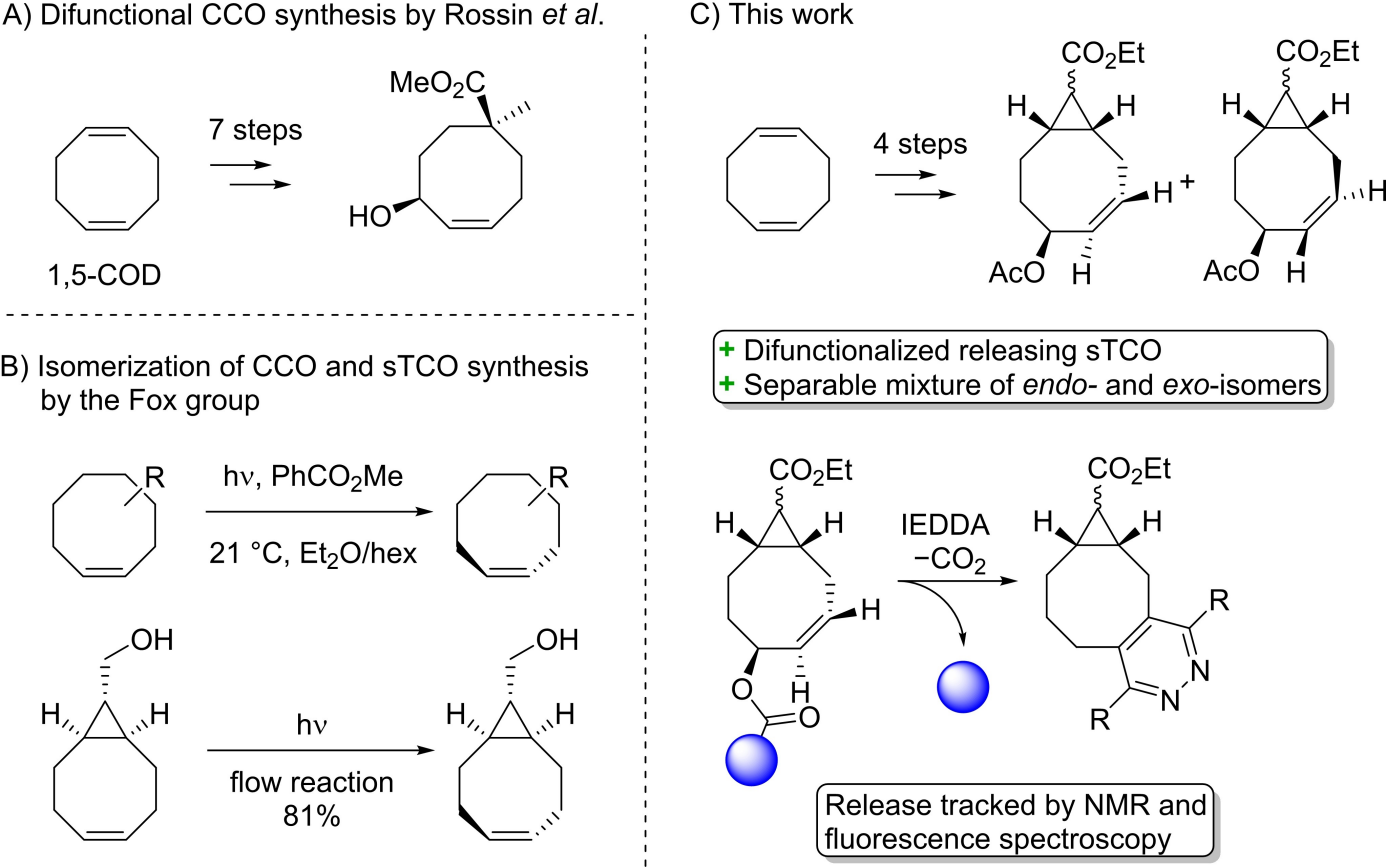
A) Difunctionalized CCO synthesis in seven steps as reported by Rossin et al. B) CCO isomerization strategy by the Fox group for the synthesis of the corresponding TCOs. C) Our work featuring a straightforward four‐step synthesis of a difunctional releasing strained TCO and the subsequent click and release studies.

In 2016 Rossin et al. reported the synthesis of the first difunctionalized *cis*‐cyclooctene (CCO) with an allylic moiety in seven steps starting from *cis,cis*‐1,5‐cyclooctadiene (1,5‐COD, Figure 1A).^[17]^ The authors reported the formation of a mixture of the axial and equatorial difunctionalized TCO derivatives after isomerization with UV‐light and a sensitizer as described by the Fox group (Figure 1B).^[25]^ Currently, the synthetic accessibility of the most commonly used difunctionalized TCOs with releasing properties is rather problematic, mainly due to low yielding steps and a lengthy overall synthesis.[^17^, ^24^] Ideally, a TCO should have multiple orthogonal handles, a fast click reaction with a tetrazine and a rapid release functionality. In comparison with the difunctionalized TCO of Rossin et al., a shorter and higher yielding synthesis method of a TCO with similar properties is desired.

Herein, we present the straightforward synthesis of a strained and allylic functionalized TCO with two orthogonal handles in only four steps starting from readily available 1,5‐COD (Figure 1C). After separation, both the *endo*‐ and *exo*‐diastereomers were diastereoselectively functionalized at the allylic position prior to the photochemical isomerization step of the *cis*‐double bond. A recent continuous flow isomerization method including an aqueous extraction to separate the *cis*‐ and *trans*‐cyclooctynes, developed by Blanco et al. was employed in this work, facilitating the scalability of this pathway.^[26]^ Functionalized derivatives of the TCO were evaluated for their releasing capabilities. This method represents a major step forward in the ease and efficiency of the synthesis of releasing TCOs.

## Results and Discussion

**Scheme 1:**
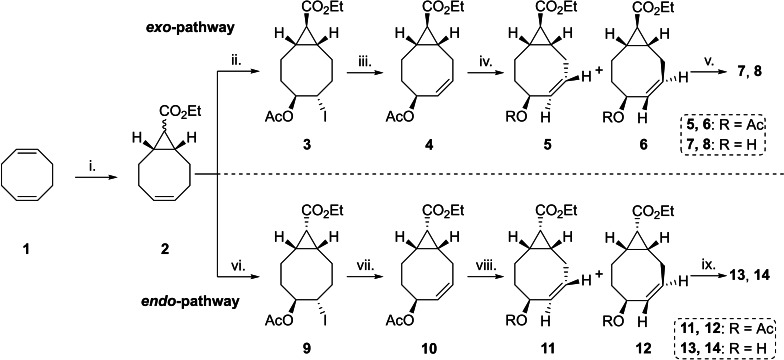
Synthesis of the difunctionalized TCO derivatives starting from commercially available *cis*,*cis*‐1,5‐cyclooctadiene (**1**). i. copper(ii) acetylacetonate (0.01 equiv), ethyl diazoacetate (0.17 equiv), 90 °C, 18 h, 61 % (*exo*/*endo* 2 : 1). ii. sodium acetate (3.0 equiv), acetic acid, NIS (1.2 equiv), 21 °C, 3 h, 83 %. iii. DBU (3.0 equiv), toluene, 100 °C, 18 h, 89 %. iv. methyl benzoate (2.03 equiv), heptane, MTBE, h*v*, silver nitrate (2.97 equiv.), 21 °C, 16 h, **5**/**6** 7 : 13, 46 %. v. potassium carbonate (2.0 equiv), ethanol, 21 °C, 18 h, **7**: 28 %, **8**: 59 %. vi. sodium acetate (3.0 equiv), acetic acid, NIS (1.2 equiv), 21 °C, 72 h, 82 %. vii. DBU (3.0 equiv), toluene, 100 °C, 48 h, 48 %. viii. methyl benzoate (0.4 equiv), heptane, h*v*, silver nitrate (2.0 equiv), 21 °C, 43 h, **11**/**12** 1 : 1, 31 %. ix. potassium carbonate (2.0 equiv), ethanol, 21 °C, 18 h, **13**/**14** 1 : 1, 43 %.

In the design of new TCOs, we were inspired by our previous work on bicyclo[6.1.0]nonyne (BCN), where we demonstrated that the fusion of a cyclopropane with the cyclooctyne induced excellent conjugation rates and kinetics due to the increased ring strain.^[8]^ Cyclopropanated CCOs have also been isomerized by the Fox group to obtain strained TCOs (sTCOs), revealing an enhanced reactivity towards tetrazines as a result of the fusion with the cyclopropane ring.[^27^, ^28^] Our synthesis commenced with monocyclopropanation of 1,5‐COD (**1**) by reaction with ethyl diazoacetate in the presence of a catalytic amount of copper(II) acetylacetonate under neat conditions to allow 1,5‐COD recovery after completion.^[29]^ This afforded a mixture of the *exo‐* and *endo‐*diastereomers of **2** (Scheme 1) in a 2 : 1 ratio, respectively (61 % total yield), which were separated by silica gel chromatography. In our experience, substitution reactions on eight‐membered rings have proven to be inefficient, therefore we envisioned that activating **2** with *N*‐iodosuccinimide (NIS) in the presence of sodium acetate and acetic acid would incorporate both the iodide and acetate in a single step as in the work of Goto and co‐workers.^[30]^ We were pleased to find that this reaction, which proceeds through the corresponding iodiranium ion, leads to highly diastereoselective acetate addition resulting in the acetylated iodohydrin **3** in 83 % yield.

**Figure 2:**
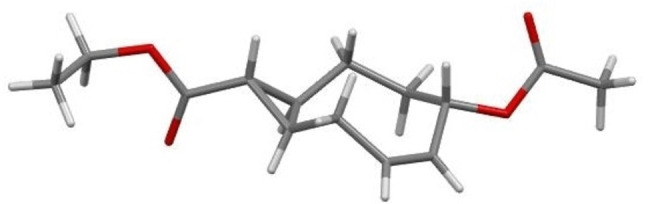
X‐ray crystal structure of compound **4**. Deposition Number 2108332 contains the supplementary crystallographic data for this paper. These data are provided free of charge by the joint Cambridge Crystallographic Data Centre and Fachinformationszentrum Karlsruhe Access Structures service.

Subsequently, a dehydroiodination reaction was performed with 1,8‐diazabicyclo[5.4.0]undec‐7‐ene (DBU) in toluene to afford difunctionalized CCO **4** in 89 % yield, presenting the Oac and CHCO_2_Et groups *cis* to each other. The stereochemical elucidation of **4** was carried out based on the single‐crystal X‐ray diffraction (Figure 2).

Compound **4** was then subsequently isomerized into the TCO derivative with UV irradiation at a wavelength of 254 nm in the presence of methyl benzoate (photosensitizer) in a continuous flow setup.[^25^, ^26^] Selective removal of the TCO with aqueous silver nitrate via liquid‐liquid extraction coupled to the photoflow system gave access to multigram amounts of the desired TCO.^14^ With the isomerization a new stereoelement was introduced, giving rise to an inseparable mixture of the *exo*‐diastereomers **5** and **6**. Fortunately, the products could be easily separated with silica gel chromatography after ethanolysis of the acetate with potassium carbonate in ethanol to furnish the corresponding hydroxylated TCOs **7** and **8**, having the allylic hydroxyl group available for functionalization with a payload.

**Scheme 2:**
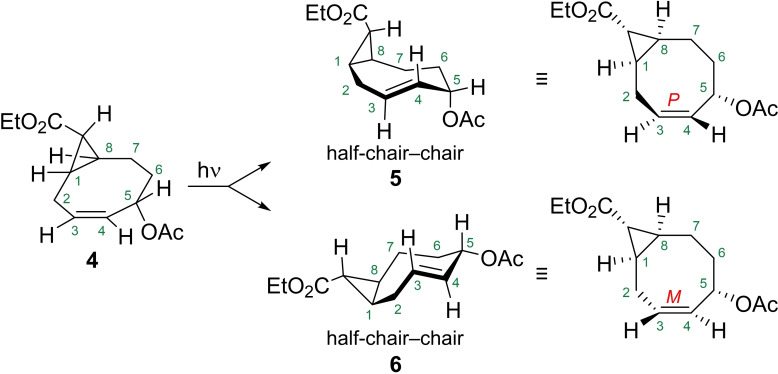
Isomerization of CCO **4** into *M*‐ and *P*‐TCOs **5** and **6**, respectively.

When the alkene of compound **4** is isomerized, the two planes C${}_{\mathrm{sp}{}^{3}}$ ‐C${}_{\mathrm{sp}{}^{2}}$ ‐H on both sides of the alkene rotate both clock‐ and counterclockwise to form *P*‐ and *M*‐cyclooctenes **5** and **6**, respectively.[^26^, ^31^] In these cases, the resulting TCOs adopt a half‐chair‐chair conformation where the acetoxy group occupies an axial position in the *P*‐isomer **5** and an equatorial position in the *M*‐isomer **6** (Scheme 2).

Next to the *exo*‐derivatives, the *endo*‐TCO analogues **13** and **14** were synthesized utilizing the same synthetic methodology (Scheme 1, *endo*‐pathway). The iodohydrin step was performed on **2** and afforded **9** diastereoselectively in 82 % yield. The *endo*‐TCO‐precursor **10** was obtained after dehydroiodination with DBU and subsequently isomerized into the corresponding *endo*‐TCOs **11** and **12** in a 1 : 1 ratio. Similar to the *exo*‐pathway, we obtained an inseparable mixture of the equatorial and axial diastereomers **11** and **12**. Unfortunately, after ethanolysis with potassium carbonate an inseparable mixture of the two diastereoisomers **13** and **14** was obtained.

**Scheme 3:**
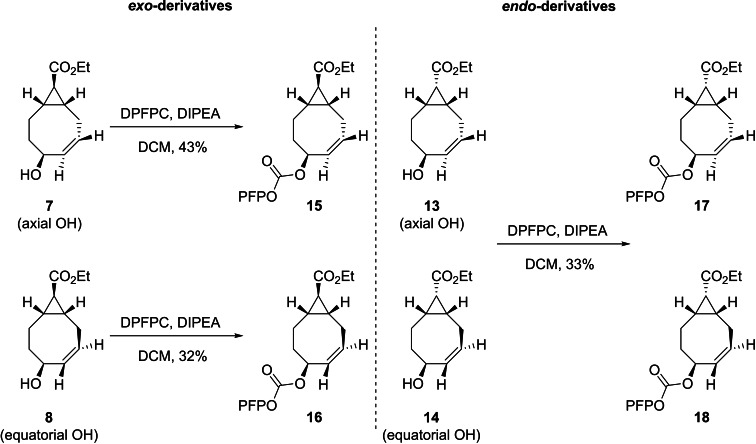
Synthesis of the PFP derivatives **15**, **16**, **17** and **18** from the corresponding TCOs **7**, **8**, **13** and **14** with DPFPC (2.75 equiv) and DIPEA (3.0 equiv) in dichloromethane, at room temperature for 18 h. The *endo*‐derivatives were obtained as an inseparable mixture of the axial (**17**) and equatorial (**18**) diastereoisomers in a 1 : 2.5 ratio respectively. PFP=pentafluorophenyl.

In order to study the click and release kinetics various allylic functionalized TCO derivatives were synthesized. First, we aimed to monitor the click and release reactions with ^19^F NMR spectroscopy by eliminating a fluorinated payload upon tetrazine activation. The allylic alcohols of the TCOs **7**, **8**, **13** and **14** were functionalized with a pentafluorophenyl (PFP) group (Scheme 3). The *exo*‐PFP derivatives were synthesized in one step from **7** and **8** with bis(pentafluorophenyl) carbonate (DPFPC) and *N*,*N*‐diisopropylethylamine (DIPEA) in dichloromethane to afford **15** and **16** in 43 and 32 % yield, respectively. Although the deacetylated *endo*‐TCOs were obtained as a mixture of axial and equatorial diastereoisomers, we still studied the release capabilities. Therefore, the *endo*‐PFP analogues **17** and **18** were synthesized in a similar fashion with DPFPC and DIPEA in an overall 33 % yield obtained as an inseparable mixture of the axial and equatorial derivatives (ratio 1 : 2.5 respectively).

**Scheme 4:**
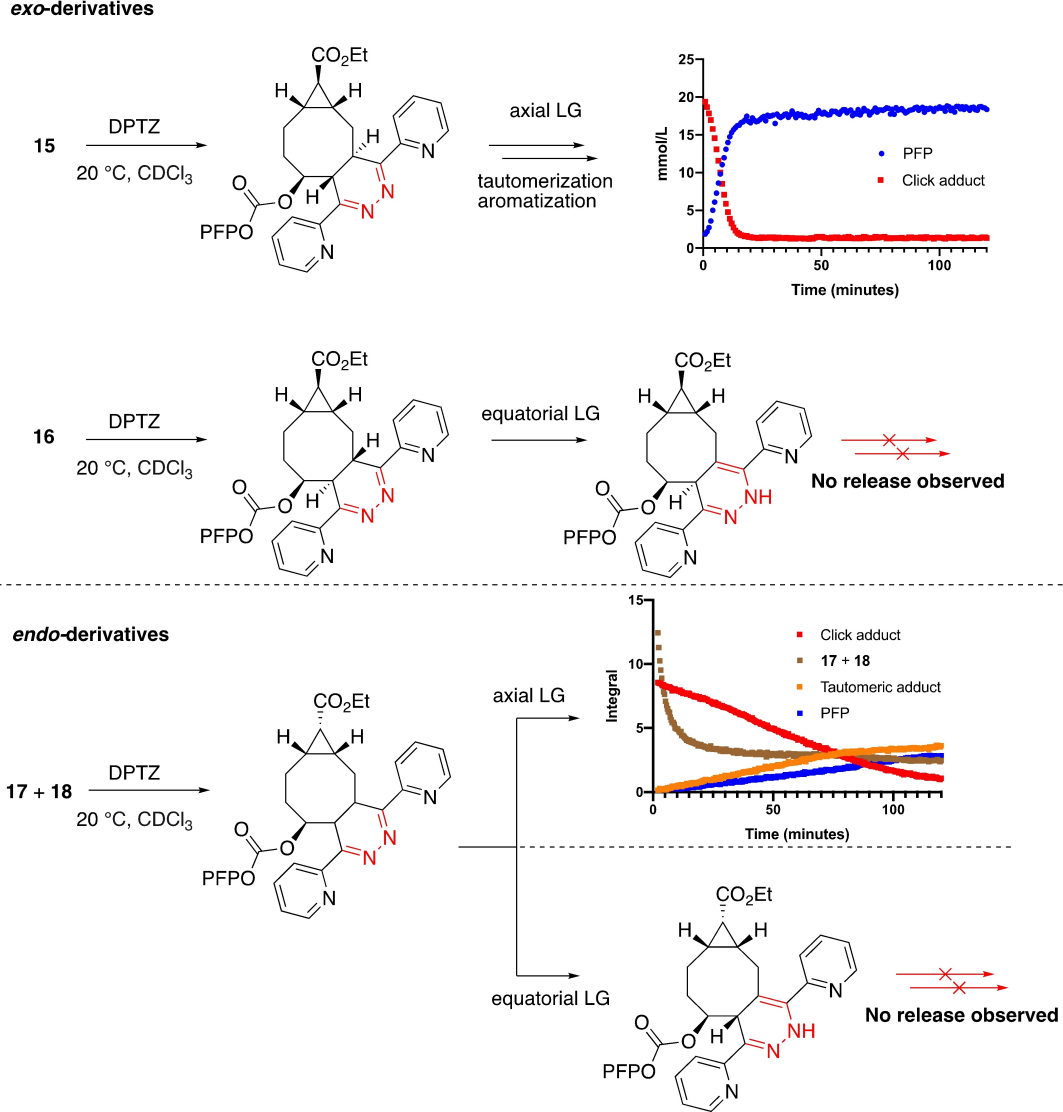
^19^F NMR click reaction kinetics study of the axial‐TCOs **15** and **17** and equatorial‐TCOs **16** and **18** by reacting them with DPTZ (**19**) in CDCl_3_. TCO **15** revealed complete release of PFP upon the click reaction within 20 minutes. In case of **16**, only the click conjugate and the tautomerized products were observed, but no release of PFP. In case of the diastereomic mixture of the *endo*‐derivatives **17** and **18**, minor release of PFP upon the click reaction of the axial isomer with **19** was observed, the equatorial primarily afforded a stable tautomeric product.

Having the new allylic functionalized TCO derivatives in hand, we set out to investigate the click and release kinetics with ^19^F NMR spectroscopy (Scheme 4). The functionalized TCOs **15**, **16**, **17** and **18** were dissolved in CDCl_3_, a solution of 3,6‐di‐2‐pyridyl‐1,2,4,5‐tetrazine (DPTZ, **19**) was added and a ^19^F NMR spectrum was recorded every 48 seconds. In case of the axial‐TCO **15**, we observed complete release of the PFP moiety within 20 minutes. Further investigation of the click and release reactions with **15** revealed that at −20 °C solely the click conjugate was observed and no release of PFP was obtained. Only after elevating the temperature to 10 °C, the PFP carbonate was released upon tautomerization from the conjugated product at such a rate that the intermediate tautomeric product could not be observed (see: Figure S8). The equatorial‐TCO **16** clearly showed conjugation with tetrazine **19**, but unfortunately no release of the PFP moiety. Instead, a stable tautomerized product with the intact PFP carbonate moiety was formed over time (Scheme 4). This rate is in line with previous studies showing that the equatorial isomer has a significant reactivity decrease because of the steric and possible electronic effects, as again confirmed in this study.[^21^, ^32^]

In case of the diastereomic mixture of the *endo*‐derivatives **17** and **18**, we observed a significantly slower click reaction under the same conditions than for the *exo*‐TCOs. Similar to the *exo*‐derivatives, the reaction was followed via ^19^F NMR spectroscopy. The different axial and equatorial intermediates and PFP product had an isolated signal which was integrated over time. The *endo*‐axial isomer **17** showed only minor PFP release within 120 minutes and the equatorial isomer **18** primarily afforded the tautomeric product and no PFP release (see: Figure S9).

Since we observed minor release of PFP with the *endo*‐TCO derivatives **17** and **18**, we focused on the *exo*‐isomers, also taking into account that the *endo*‐TCO (**13** and **14**) was obtained as an inseparable diastereomeric mixture. Besides, the precursor was synthesized in an overall lower yield as compared to the *exo*‐one and an excess of the *exo*‐derivative was formed in the cyclopropanation step (**2**, 2 : 1 ratio). Conversely, we showed that the mixture of isomers in **2** can be isomerized solely into the *exo‐*isomer with 1 M potassium *tert*‐butoxide in THF and dry ethanol (data not shown).^[33]^

Next, the click kinetics of the axial *exo*‐TCO **7** were investigated upon reaction with a single equivalent of tetrazine **19** or **20**. The second‐order rate constants were determined in acetonitrile at room temperature.^[21]^ For the click reaction of tetrazine **19** with **7** at 188 μM we found a *K*_2_=594.0 M^−1^ s^−1^±12.38 and for tetrazine **20** *K*_2_=0.8589 M^−1^ s^−1^±0.001, as also expected due to increased electron density of tetrazine **19** (see: Table 1). When the click reaction was carried out at 97 μM we encountered similar values. These results seem to demonstrate that our new TCO outperforms the currently available releasing difunctional TCOs in the literature in terms of click kinetics.

**Table 1:** Second‐order rate constants for reaction of TCO **7** with tetrazines **19** and **20**. Values were determined using UV/Vis spectroscopy at 540 nm (specific for tetrazine) in acetonitrile at 21 °C.

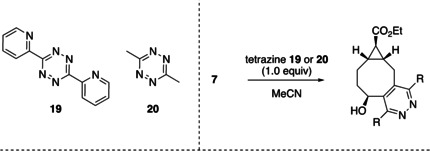		
**tetrazine**	97 μM	188 μM
**19**	516.2 M^−1^ s^−1^±16.78	594.0 M^−1^ s^−1^±12.38
**20**	0.9864 M^−1^ s^−1^±0.004	0.8589 M^−1^ s^−1^±0.001

Next to the carbonate‐linked PFP TCOs, we wanted to explore more stable carbamate linked derivatives, as these have already been reported in the literature to provide successful release.^[22]^ We synthesized fluorescently caged *exo*‐TCO derivatives by coupling 7‐amino‐4‐methylcoumarin (AMC) via a carbamate linkage to the allylic alcohol on our TCO. With this approach we could directly asses the release properties of our system with straightforward fluorescence measurements, since the characteristic fluorescence of AMC can easily be quenched by modifying the free amine as also demonstrated in similar TCO‐AMC release studies in the recent literature.[^34^, ^35^] The best synthesis results for the fluorescent TCO derivatives **21** and **22** were obtained by first forming the isocyanate of AMC with triphosgene and DIPEA in toluene at reflux temperature. Subsequently, the TCO derivative with the free allylic hydroxyl **7** or **8** and 4‐(dimethylamino)pyridine (DMAP) were dissolved in dichloromethane and the AMC‐isocyanate was added. This afforded both the axial and equatorial *exo*‐TCO derivatives **21** and **22** in 63 and 42 % yield, respectively.

**Scheme 5:**
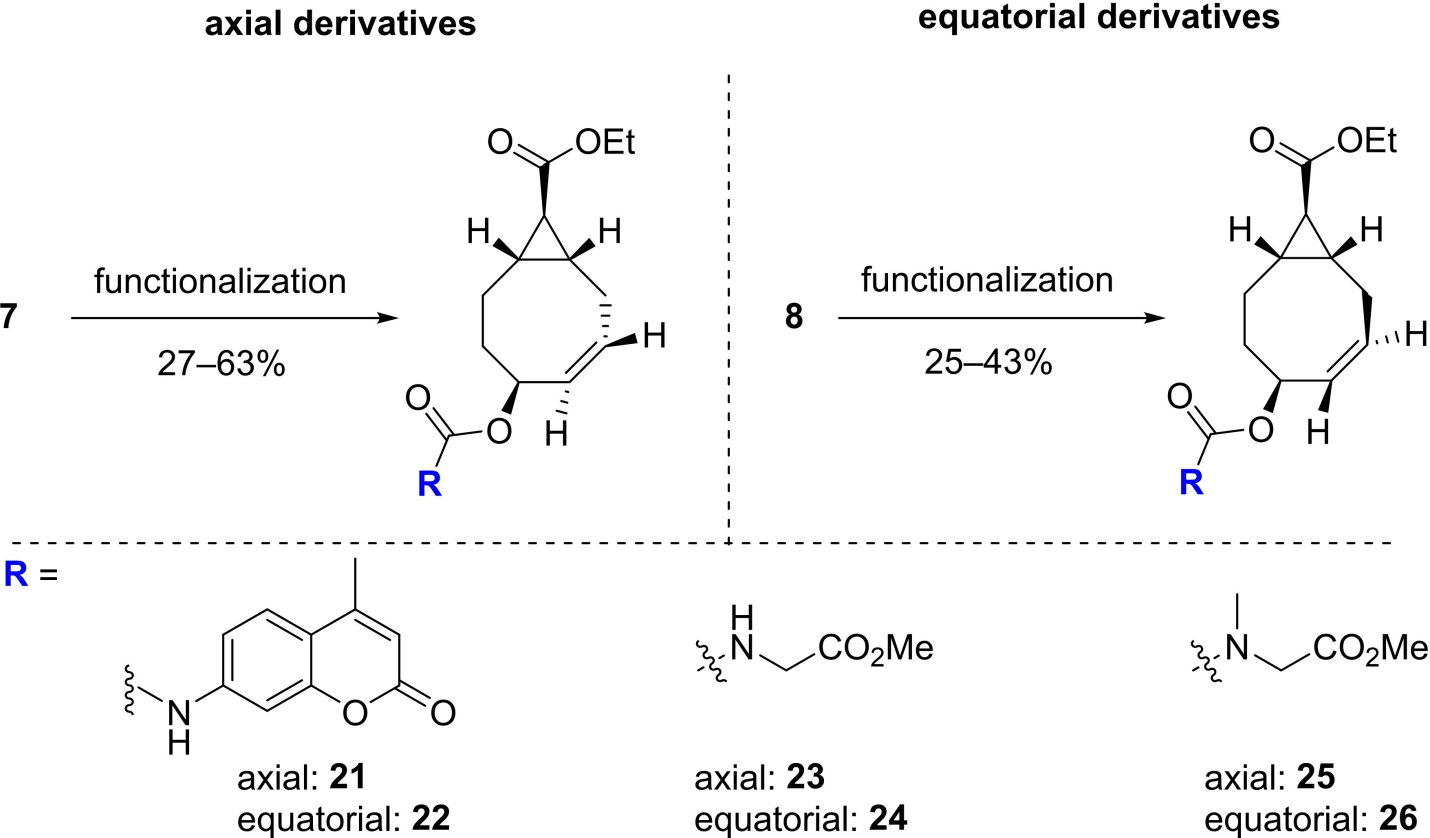
Synthesis of the functionalized *exo*‐TCO carbamate derivatives from the axial and equatorial isomers **7** and **8**. **21**/**22**: 7‐amino‐4‐methylcoumarin (1.25 equiv), triphosgene (1.25 equiv.), DIPEA (4.6 equiv.), DMAP (3.0 equiv.), toluene, DCM, 21 °C, 18 h, 42–63 %. **23**/**24**: i. DPFPC (2.0 equiv.), DIPEA (5.0 equiv.), DMAP (0.1 or 5.0 equiv.), MeCN. ii. glycine methyl ester hydrochloride (5.0‐7.0 equiv.), DIPEA (2.5‐4.5 equiv.), MeCN, 21 °C, 18 h, 27–42 %. **25**/**26** i. DPFPC (2.0 equiv.), DIPEA (5.0 equiv.), DMAP (0.1 equiv.), MeCN. ii. sarcosine methyl ester hydrochloride (2.0 equiv.), DIPEA (2.5 equiv), MeCN, 21 °C, 18 h, 25–31 %.

Moreover, we also synthesized aliphatic carbamate derivatives of the *exo*‐TCOs to further elucidate the scope of their release properties (Scheme 5). To this extent, we coupled a glycine or sarcosine derivative to the allylic alcohol of the TCO. These compounds were chosen with the work of Carlson and co‐workers in mind, who showed that an unmethylated nitrogen ‐ as in the glycine derivative ‐ can result in formation of a dead‐end product, while an *N*‐methylated nitrogen as in sarcosine should prevent this.^[36]^ All compounds were generated in a two‐step synthesis, wherein hydroxy TCOs **7** and **8** were first activated with DPFPC, DIPEA and DMAP. In the second step, the amine was added in a basic environment to form the desired carbamate. The glycine derivatives **23** and **24** were obtained in 27 and 42 % yield and the sarcosine derivatives **25** and **26** in 31 and 25 %, respectively.

**Figure 3:**
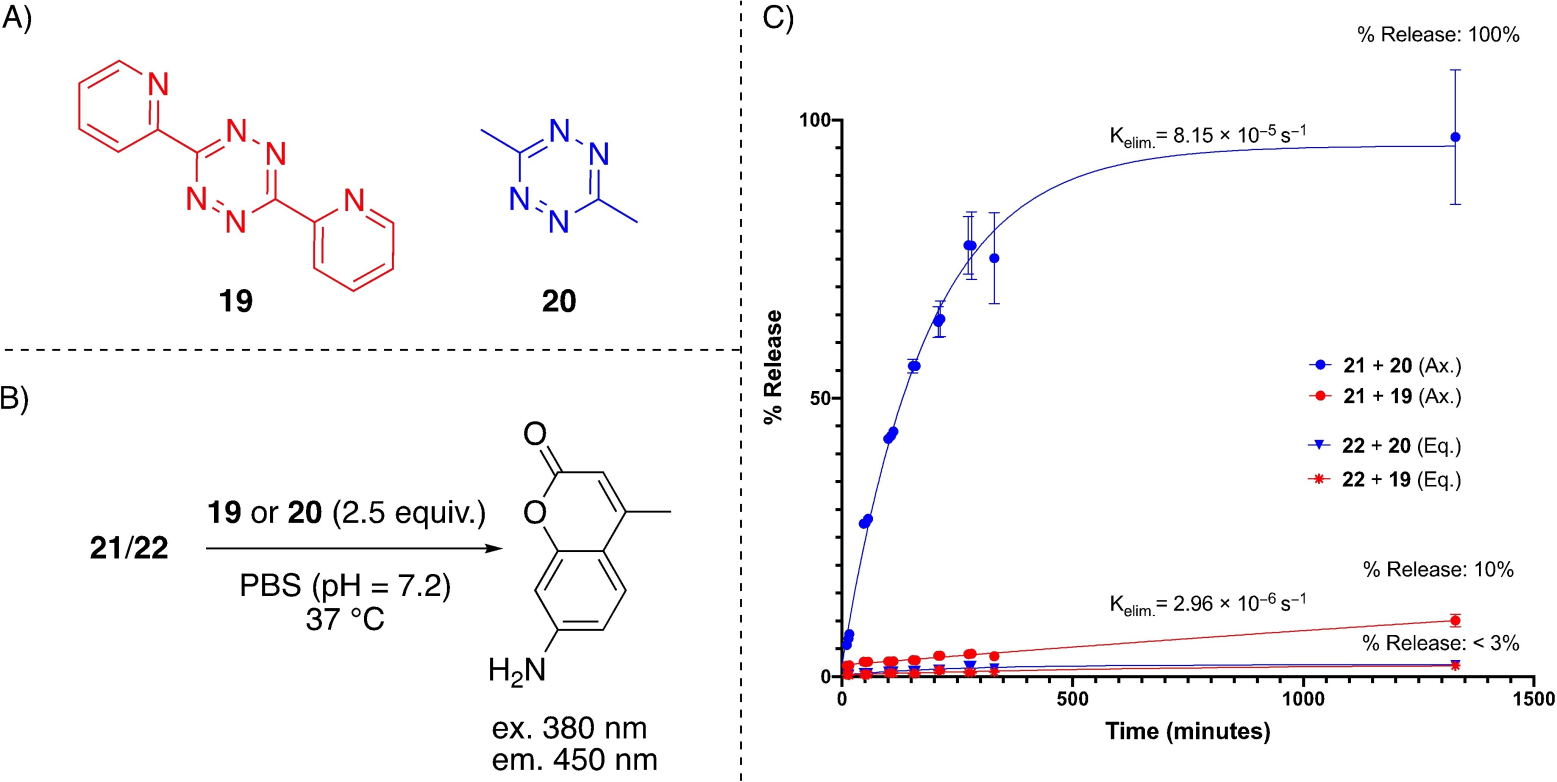
Release study of the caged fluorescent TCO derivatives **21** and **22** by the click and release reaction with tetrazine **19** or **20**. A) The used tetrazines in this study. B) Study conditions, either **21** or **22** was incubated with tetrazine **19** or **20** and the fluorescence (excitation: 380 nm, emission: 450 nm) was measured at certain time points after the addition of the tetrazine. C) The release efficiency plotted as a function over time.

Next, we examined the elimination kinetics of the caged fluorescent TCO derivatives **21** and **22**. Both derivatives were incubated in phosphate‐buffered saline (PBS, pH=7.4) and subsequently a 2.5‐fold excess of either tetrazine **19** or **20** was added. The characteristic fluorescence of the released AMC (excitation 380 nm, emission 450 nm) was measured as a function over time in a plate reader. As positive control the maximum amount of possible released AMC was also measured, in this way the releasing efficiency could readily be calculated. As expected, and also observed in our ^19^F NMR experiments, the equatorial‐TCO derivative **22** furnished minimal release of the fluorescent AMC moiety with tetrazine **19** or **20** (<3% in 22 h, Figure 3). Nonetheless, the axial analogue **21** afforded complete release of the fluorescent AMC within 23 h upon ligation with **20**, after less than three hours already more than 50 % release was observed (*k*_elim_=8.15×10^−5^ s^−1^). As anticipated, the elimination reaction was heavily depending on the used tetrazine, as for tetrazine **19** the release was significantly slower (10 % within 23 h, *k*_elim_=2.96×10^−6^ s^−1^), as also confirmed in the recent literature.^[35]^

**Figure 4:**
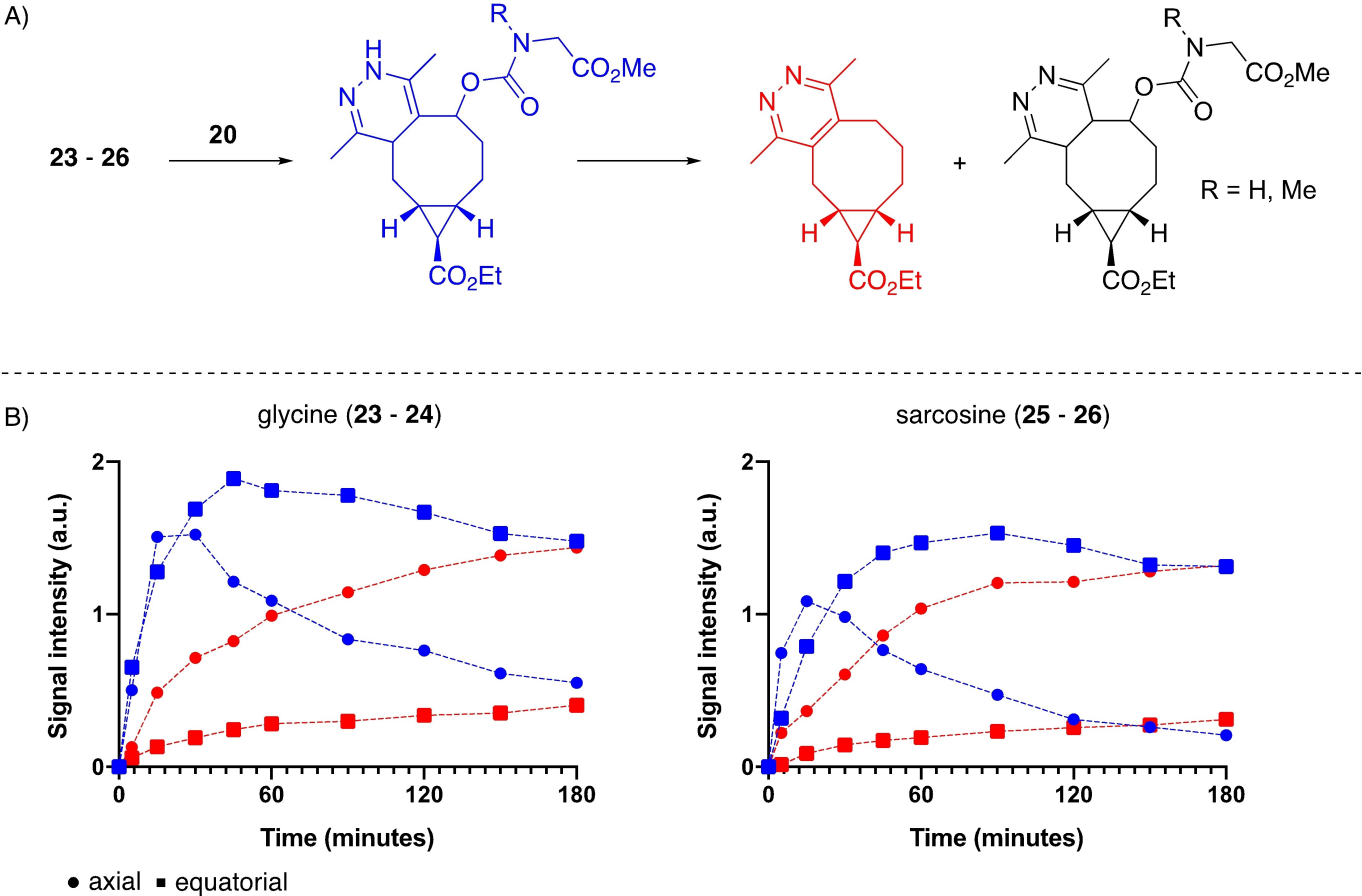
Release study of the allylic amine TCO derivatives **23**–**26**. A) Studied reaction of TCO derivatives **23**–**26** and 3,6‐dimethyl‐1,2,4,5‐tetrazine (**20**). B) The signal intensity of the protons measured in the ^1^H NMR study of the tetrazine methyl‐protons of the intermediate (in blue) and release product (in red) of the axial (•) and equatorial (▪) isomers.

Hereafter, tetrazine **20** was chosen to compare the four allylic carbamate TCO derivatives **23**‐**26**. The derivatives were dissolved in a mixture of deuterated methanol and PBS (1 : 1, v/v) and hereafter **20** was added. ^1^H NMR spectra were measured over a period of three hours. All derivatives revealed a complete click reaction with **20** within the first five minutes. The axial compounds **23** and **25** showed rapid release of the amino acid payloads (Figure 4, in red) accompanied by a respective decrease in the initial click conjugates (Figure 4, in blue). The respective equatorial derivatives **24** and **26** revealed less release and a more gradual decrease of the click conjugate. Compared to their axial analogues, 38 % of the glycine equatorial isomer **24** and 23 % of the sarcosine equatorial isomer **26** released payload successfully.

Hereafter, the stability of the releasing axial TCO was investigated in both PBS and mouse serum at 37 °C. The free carboxylic derivative of **7** dissolved in PBS showed no changes in the ^1^H NMR spectrum after seven days (see: Figure S1). TCO **7** was stable in mouse serum up to several hours and showed no isomerization into the CCO analogue, however, slow degradation of **7** over time was observed (see: Figure S2).

**Figure 5:**
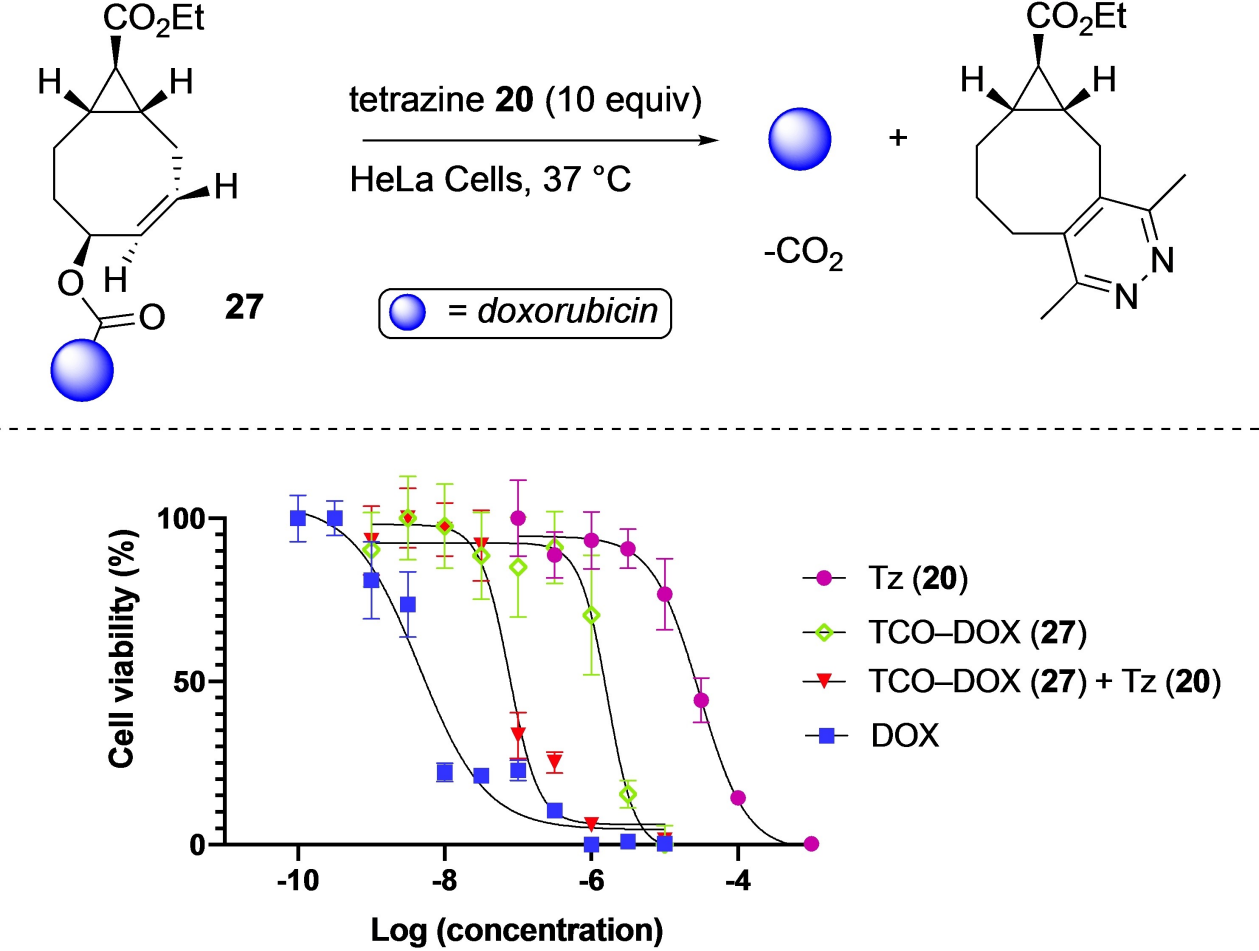
Doxorubicin cellular toxicity study of **27** induced by the click reaction with tetrazine **20** in HeLa Cells. The cells were incubated for three days with the indicated compounds at 37 °C after which cellular viability was assessed using a CCK‐8 assay. EC_50_ curves and values for 3,6‐dimethyl‐1,2,4,5‐tetrazine (Tz (**20**), in purple), TCO‐doxorubicin (TCO‐DOX (**27**), in green), TCO‐doxorubicin with 10 equiv. of tetrazine **20** (TCO‐DOX (**27**)+Tz (**20**), in red) and doxorubicin (DOX, in blue).

After we observed that the new TCO gave satisfactory payload release, we decided to synthesize a drug‐conjugated TCO that would release the drug upon tetrazine ligation. This was examined with the release of the chemotherapeutic drug doxorubicin in a cellular environment.^[21]^ The doxorubicin conjugate **27** was synthesized from the 4‐nitrophenyl activated TCO and purified with reversed‐phase HPLC. The effectiveness of doxorubicin release was tested in HeLa cells through an EC_50_ assay (Figure 5). The cells were incubated for three days at 37 °C with varying concentrations of the indicated compounds (tetrazine **20**, doxorubicin, TCO‐Dox (**27**), or TCO‐Dox (**27**)+10 equiv. tetrazine **20**). Addition of 10 equivalents of tetrazine **20** increased the cytotoxicity of the doxorubicin TCO conjugate **27** with a 21‐fold (75.9±12.08 nM vs. 1.654±0.418 μM) which indicates successful release of doxorubicin (Table 2). This change cannot be attributed to toxicity of the tetrazine, as its EC_50_ was in the mid‐micromolar range (29.81±5.21 μM). Together, these findings confirmed that conjugation to the TCO reduces doxorubicin's toxicity and that the drug can effectively be released upon tetrazine activation. Besides, HPLC analysis of the same click reaction in solution revealed successful and rapid release of doxorubicin from the conjugate (see: Figure S14).

**Table 2:** EC_50_ values for tetrazine **20**, TCO‐DOX (**27**), TCO‐DOX (**27**)+10 equiv. **20** and doxorubicin from proliferation assays in HeLa cells determined using a CCK‐8 assay.

**Compound**	**EC_50_**	**95 % CI** ^[a]^
**Tetrazine 20**	29.81 μM	24.60–36.40 μM
**TCO‐DOX 27**	1.654 μM	1.236–2.217 μM
**TCO‐DOX 27+10 equiv 20**	0.076 μM	0.064–0.096 μM
**Doxorubicin**	**0.0045 μM**	**0.0031–0.0036 μM**

[a] EC_50_ 95 % confidence interval (n=6).

## Conclusion

In conclusion, we have developed a straightforward synthesis of a novel difunctionalized strained TCO with cargo‐releasing capabilities in only four high yielding steps. The acetylated iodohydrin intermediate and the subsequent elimination reaction were key steps in this pathway to afford the TCOs in excellent yields. To our knowledge this is the shortest reported synthesis of difunctionalized TCOs with releasing properties allowing improved access to these click reagents for both diagnostic and therapeutic applications. Furthermore, because of the high yields and the continuous photoflow isomerization process our pathway is amenable for larger scale synthesis. The TCOs have two orthogonal handles so that derivatization with different moieties is possible, while the allylic alcohol enables release of a payload upon click reaction with a tetrazine derivative, as demonstrated with carbonate and carbamate linked cargo molecules, even in a cellular environment. Moreover, we identified a TCO that fully retains the allylic moiety upon the click reaction. This product may be valuable for non‐releasable applications requiring stable conjugates with orthogonal handles on the TCO. The broad range applicability of the TCO‐tetrazine click and release system combined with this efficient synthetic accessibility will in our view strongly contribute to new in vitro and in vivo biomedical applications in the near future.

## Experimental Section

***exo*** **‐CCO 3**: A suspension of cyclooctene **2** (20.4 g, 105 mmol) and sodium acetate (25.8 g, 315 mmol) in acetic acid (240 mL) was stirred and cooled with a water bath until the temperature stabilized to ambient temperature. Next, *N*‐iodosuccinimide (28.4 g, 126 mmol) was added and the mixture was stirred at ambient temperature for 3 h. After full conversion was observed on TLC, brine (50 mL) and heptane (200 mL) were added to the reaction mixture. The organic layer was collected and washed with brine (250 mL), a mixture of saturated aqueous sodium thiosulfate (50 mL), brine (200 mL) followed by saturated aqueous sodium bicarbonate (200 mL). The aqueous layers were back‐extracted in similar order with two additional portions of heptane (2×200 mL). The combined organic layers were dried with MgSO_4_ and concentrated to afford the crude product (39 g). This was purified with silica gel column chromatography (25→75 % EtOAc in heptane) to afford *exo*‐CCO **3** (33.2 g, 83 %) as white crystals. TLC (EtOAc/heptane, 1 : 3 v/v): *R*_F_=0.14. ^1^H NMR (400 MHz, CDCl_3_) δ 4.86 (ddd, *J*=7.7, 6.6, 3.5 Hz, 1H), 4.75 (td, *J*=7.8, 5.3 Hz, 1H), 4.08 (q, *J*=7.2 Hz, 2H), 2.24–2.17 (m, 2H), 2.15–2.08 (m, 1H), 2.07 (s, 3H), 2.05–1.96 (m, 2H), 1.84–1.70 (m, 1H), 1.54–1.46 (m, 1H), 1.46–1.38 (m, 1H), 1.34–1.25 (m, 2H), 1.23 (t, *J*=7.1 Hz, 3H), 1.15 (t, *J*=4.3 Hz, 1H). ^13^C NMR (101 MHz, CDCl_3_) δ 173.6, 169.7, 73.3, 60.4, 37.5, 33.7, 33.2, 26.1, 26.1, 26.0, 25.7, 23.6, 21.4, 14.2.

***exo*** **‐CCO 4**: DBU (39.5 mL, 262 mmol) was added to a stirred solution of CCO **3** (33.2 g, 87.3 mmol) in toluene (279 mL, 2.62 mol). The mixture was stirred at 100 °C for 18 h. The reaction mixture was cooled with an ice bath and filtered to remove the solids. The residue was washed with toluene (2×50 mL). The filtrate was washed with brine (2×250 mL) and the aqueous layers were back‐extracted with toluene (100 mL). The combined organic layers were dried with MgSO_4_ and concentrated to afford a yellow oil which was purified with silica gel column chromatography (10→20 % EtOAc in heptane) to afford CCO **4** (19.6 g, 89 %). TLC (EtOAc/heptane, 1 : 4 v/v): *R*_F_=0.26. ^1^H NMR (500 MHz, CDCl_3_) δ 5.81 (dddd, *J*=11.3, 9.2, 6.9, 2.0 Hz, 1H), 5.62 (dd, *J*=11.1, 5.3 Hz, 1H), 5.32 (dd, *J*=10.6, 5.2 Hz, 1H), 4.10 (q, *J*=7.2 Hz, 2H), 2.44–2.34 (m, 2H), 2.05 (s, 3H), 1.91–1.85 (m, 1H), 1.79–1.63 (m, 3H), 1.44–1.28 (m, 3H), 1.25 (t, *J*=7.1 Hz, 3H). ^13^C NMR (126 MHz, CDCl_3_) δ 173.5, 170.1, 133.9, 129.1, 75.3, 60.3, 36.4, 31.2, 28.6, 26.9, 26.4, 25.4, 21.3, 14.2. HRMS (m/z): [M+Na]^+^ calcd. for C_14_H_20_O_4_Na: 275.1259, found 275.1240.

***exo*** **‐TCOs 5 and 6**: A custom‐made long‐necked flask was charged with an aqueous solution of silver nitrate (7.00 g, 41.2 mmol) in water (100 mL). Next, a solution of **4** (3.50 g, 150 mmolar, 13.9 mmol) and methyl benzoate (3.84 g, 3.56 mL, 28.2 mmol) in deoxygenated heptane (160 mL) and MTBE (40 mL) was loaded into a UV irradiation setup (as described in Blanco‐Ania et al.).^[26]^ The continuous process ran for 16 h. Next, the biphasic reaction mixture was loaded into a separation funnel and the aqueous layer was collected. The organic layer was washed with water (100 mL). The combined aqueous layers were washed with heptane (100 mL). Subsequently, 25 % aqueous ammonium hydroxide (25 mL) was added to the aqueous layer before extracting it with EtOAc (100 mL). The combined organic layers were dried with MgSO_4_ and concentrated to afford an unseparable diastereomeric mixture consisting of a 7 : 13 ratio of the axial and equatorial TCO‐isomers **5** and **6** as a clear oil (1.6 g, 46 %). HRMS (m/z): [M+Na]^+^ calcd. for C_14_H_20_O_4_Na: 275.1259, found 275.1264. **5** (axial): TLC (EtOAc/heptane, 1 : 1 v/v): *R*_F_=0.54. ^1^H NMR (500 MHz, CDCl_3_) δ 6.11 (ddd, *J*=16.8, 10.9, 5.9 Hz, 1H), 5.55 (dd, *J*=16.9, 3.4 Hz, 1H), 5.12 (q, *J*=3.1 Hz, 1H), 4.14–4.05 (m, 2H), 2.67 (dt, *J*=12.6, 6.0 Hz, 1H), 2.51–2.45 (m, 1H), 2.25–2.20 (m, 1H), 2.07 (m, 1H), 2.05 (s, 3H), 2.00–1.95 (m, 1H), 1.80–1.75 (m, 1H), 1.60–1.55 (m, 2H), 1.50–1.45 (m, 1H), 1.25 (m, 3H). ^13^C NMR (126 MHz, CDCl_3_) δ 174.9, 170.0, 131.7, 129.5, 70.7, 60.5, 37.2, 31.3, 29.2, 26.7, 21.1, 20.6, 14.2. **6** (equatorial): TLC (EtOAc/heptane, 1 : 1 v/v): *R*_F_=0.54. ^1^H NMR (500 MHz, CDCl_3_) δ 5.91 (ddd, *J*=16.0, 9.1, 6.4 Hz, 1H), 5.67 (ddd, *J*=16.6, 9.7, 1.5 Hz, 1H), 5.01 (td, *J*=9.8, 5.3 Hz, 1H), 4.14–4.05 (m, 2H), 2.86 (dtt, *J*=14.7, 9.2, 1.1 Hz, 1H), 2.40–2.35 (m, 1H), 2.38–2.30 (m, 2H), 2.18 (m, 2H), 2.06 (s, 3H) 1.36 (t, *J*=5.6 Hz, 1H), 1.25 (m, 3H), 1.25‐1.20 (m, 1H), 0.77 (dt, *J*=15.4, 11.4 Hz, 1H). ^13^C NMR (126 MHz, CDCl_3_) δ 174.2, 170.6, 136.1, 131.2, 77.2, 60.4, 37.6, 36.6, 34.6, 30.2, 29.0, 28.4, 21.2, 14.2.

***exo*** **‐TCOs 7 and 8**: A solution of the *P*‐ and *M*‐isomers **5** and **6** (2.00 g, 7.93 mmol) and potassium carbonate (2.19 g, 15.9 mmol) in ethanol (19.9 mL, 341 mmol) was stirred at 21 °C for 18 h. The flask was shielded from light with aluminum foil. After completion of the reaction was observed with TLC, acetic acid (1.91 mL, 33.3 mmol) in a 1 : 1 water‐brine mixture (50 mL) was added to quench the reaction. Then, EtOAc (25 mL) was added and separated from the aqueous layer, the organic layer was washed with aqueous saturated sodium bicarbonate (25 mL) and then with brine (25 mL). All aqueous layers were back‐extracted with EtOAc (2×25 mL). The combined organic layers were dried with MgSO_4_, concentrated *in vacuo* and purified with silica gel column chromatography (10 % acetone in toluene) to afford **7** (470 mg, 28 %) and **8** (980 mg, 59 %). TCO **7** (EtOAc/heptane, 1 : 1 v/v): *R*_F_=0.37. ^1^H NMR (500 MHz, CDCl_3_) δ 6.26 (ddd, *J*=16.8, 10.9, 5.9 Hz, 1H), 5.55 (ddd, *J*=16.8, 3.1, 0.9 Hz, 1H), 4.36 (q, *J*=3.0 Hz, 1H), 4.08 (qd, *J*=7.1, 2.1 Hz, 2H), 2.66 (dt, *J*=12.5, 6.0 Hz, 1H), 2.49–2.42 (m, 1H), 2.25 (dd, *J*=16.1, 11.9 Hz, 1H), 2.00–1.91 (m, 1H), 1.87 (q, *J*=8.0 Hz, 1H), 1.79–1.74 (m, 1H), 1.68 (t, *J*=13.1 Hz, 1H), 1.55–1.48 (m, 2H), 1.46–1.40 (m, 1H), 1.24 (t, *J*=7.2 Hz, 3H). ^13^C NMR (126 MHz, CDCl_3_) δ 175.2, 133.5, 130.4, 68.5, 60.4, 39.7, 31.7, 29.1, 27.0, 20.5, 19.3, 14.2. TCO **8** (equatorial): TLC (EtOAc/heptane, 1 : 1 v/v): *R*_F_=0.27. ^1^H NMR (500 MHz, CDCl_3_) δ 5.76 (ddd, *J*=15.8, 8.9, 6.5 Hz, 1H), 5.62 (ddd, *J*=16.5, 9.4, 1.4 Hz, 1H), 4.08 (dd, *J*=9.8, 5.2 Hz, 1H), 4.05 (q, *J*=7.1 Hz, 2H), 2.81 (dtt, *J*=14.8, 9.3, 1.2 Hz, 1H), 2.34–2.29 (m, 1H), 2.28–2.23 (m, 1H), 2.16–2.08 (m, 1H), 2.10–2.05 (m, 1H), 1.43 (dddd, *J*=13.1, 11.4, 9.7, 0.8 Hz, 1H), 1.32–1.29 (m, 1H), 1.20 (t, *J*=7.1 Hz, 3H), 1.18–1.13 (m,1H), 0.68 (dt, *J*=15.4, 11.3 Hz, 1H). ^13^C NMR (126 MHz, CDCl_3_) δ 174.5, 140.3, 129.3, 76.1, 60.4, 38.0, 37.7, 30.3, 29.3, 28.6, 28.5, 14.2.

## Conflict of interest

The authors declare no conflict of interest.

1

## Supporting information

As a service to our authors and readers, this journal provides supporting information supplied by the authors. Such materials are peer reviewed and may be re‐organized for online delivery, but are not copy‐edited or typeset. Technical support issues arising from supporting information (other than missing files) should be addressed to the authors.

Supporting Information

## Data Availability

The data that support the findings of this study are available in the supplementary material of this article.
