# Supplementary material for: Readily Accessible Strained Difunctionalized trans‐Cyclooctenes with Fast Click and Release Capabilities
Source: Chemistry. 2022 Dec 7;29(6):e202203375. doi: 10.1002/chem.202203375 (PMC10107714; doi:10.1002/chem.202203375)
Supplement: Supplementary file 1 — Supporting Information [file CHEM-29-0-s001.pdf]

Chemistry–A European Journal

Supporting Information

Readily Accessible Strained Difunctionalized *trans*-Cyclooctenes with Fast Click and Release Capabilities

Daan Sondag, Luuk Maartense, Heleen de Jong, Frank F. J. de Kleijne, Kimberly M. Bonger, Dennis W. P. M. Löwik, Thomas J. Boltje, Jan Dommerholt, Paul B. White, Daniel Blanco-Ania, and Floris P. J. T. Rutjes*

Table of Contents

1. General S3
    a. Material and methods S3
2. Synthesis S4 – S26
    a. TCO synthesis S4 – S6
    b. *exo*-TCO compounds S7 – S10
    c. *endo*-TCO compounds S11 – S14
    d. *exo* axial derivatives S15 – S18
    e. *exo* equatorial derivatives S19 – S22
    f. *endo* derivatives S23 – S24
    g. Doxorubicin *exo* releasing derivative S25 – S26
3. Stability assays S27 – S29
    a. Stability assay in PBS S27 – S28
    b. Stability assay in mouse serum S29
4. Kinetics study of the click and release S30 – S43
    a. Click reaction kinetics S30 – S32
    b. PFP carbonate release study with ^{19}F NMR S33 – S35
    c. AMC carbamate release study with fluorescence spectroscopy S36
    d. Glycine/sarcosine release study with ^{1}H-NMR S37 – S40
    e. Cell viability assay S41
    f. Doxorubicin release HPLC study S42 – S43
5. Rotamer determination S44 – S45
6. NMR spectra S46 – S76
7. References S77

1. General

a. Material and methods

NMR spectra were recorded on a Bruker Avance III 400 MHz or a Bruker 500 MHz spectrometer and the compounds were assigned using ^{1}H NMR, ^{13}C NMR, COSY, HSQCED, HMBC and NOESY spectra. Chemical shifts were reported in parts per million (ppm.) relative to reference ($CDCl_3$: ^{1}H: 7.26 ppm. and ^{13}C 77.16 ppm.; CD_3OD: ^{1}H: 3.31 ppm. and ^{13}C 49.00 ppm.; D_2O: ^{1}H: 4.79 ppm.; $(CD_3)_2SO$: ^{1}H: 2.50 ppm. and ^{13}C 39.52 ppm.). NMR data are presented in the following way: chemical shift, multiplicity (s = singlet, bs = broad singlet, d = doublet, t = triplet, dd = doublet of doublets, ddd = doublet of doublet of doublets, dtd = doublet of triplet of doublets h = heptet, m = multiplet and/or multiple resonances) and coupling constants J in Hz. Mass spectra were recorded on a JEOL AccuTOF CS JMS-T100CS (ESI) mass spectrometer. Automatic flash column chromatography was executed on a Biotage Isolera Spektra One using SNAP or Silicycle cartridges (Biotage, 30–100 µm, 60 Å) 4–50 g. Reactions under protective atmosphere were performed under positive Ar./N_2 flow using flame-dried glassware. The photoirradiation was done with a Rayonet RMR-600 photoreactor (254 nm) with FEP tubing (i.d. 1.57 mm, o.d. 3.18 mm; distance between the tubing and the UV lamp = 4.0 cm) and a self-fabricated photoreactor consisting of a OSAGA RVS UV-C 55w pond filter with a Philips PL-L 55 W UV-C lamp (254 nm) and FEP tubing (i.d. 2.7 mm, o.d. 3.18 mm; distance between the tubing and the UV lamp = 0.7 – 1.4 cm). Preparative HPLC was performed on a Phenomenex® Gemini-NX 3u C18 110A reversed-phase column (150 × 21.2 mm) using gradient elution with a constant flow of 10 mL/min at 30 °C. MiliQ (0.1% formic acid) and CH_3CN (0.1% formic acid) were used as the solvents. The pure fractions containing product were combined and lyophilized overnight to yield the target compounds. Tetrazines **19**, **20** and doxorubicin hydrochloride were bought (TCI Europe N.V.). Mouse serum for stability assay was also bought commercially (Invitrogen).

2. Synthesis

a. TCO synthesis

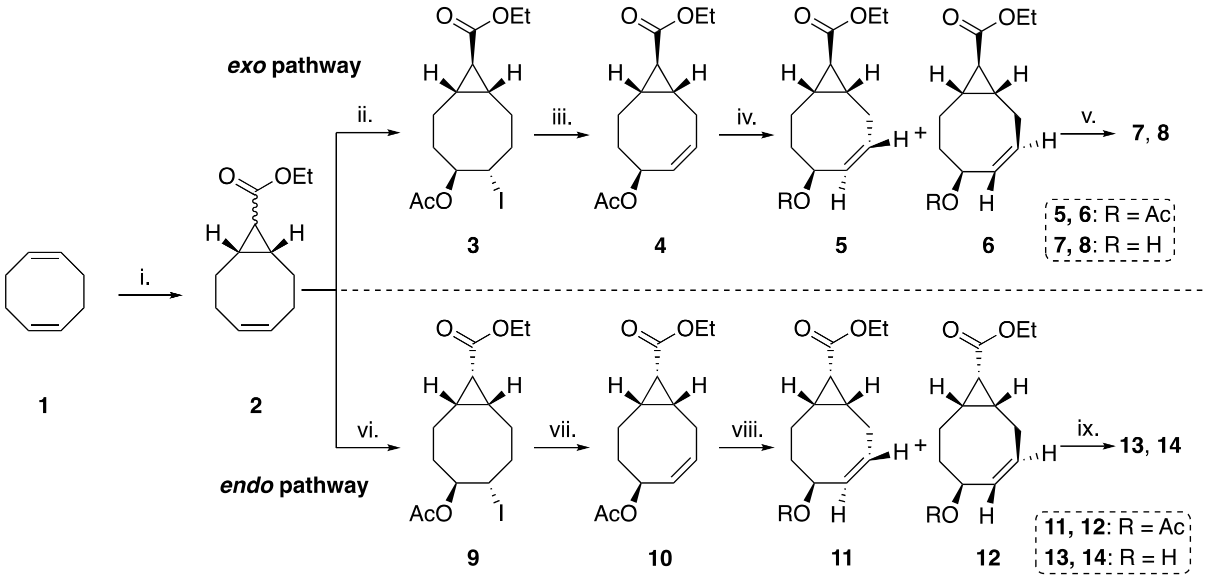

Scheme S1: i. copper(ii) acetylacetonate (0.01 equiv), ethyl diazoacetate (0.17 equiv), 90 °C, 18 h, 61% (*exo*/*endo* 2:1). ii. sodium acetate (3.0 equiv), acetic acid, NIS (1.2 equiv), 21 °C, 3 h, 83%. iii. DBU (3.0 equiv), toluene, 100 °C, 18 h, 89%. iv. methyl benzoate (2.03 equiv), heptane, MTBE, h*ν*, silver nitrate (2.97 equiv.), 21 °C, 16 h, **5**/**6** 7:13, 46%. v. potassium carbonate (2.0 equiv), ethanol, 21 °C, 18 h, **7**: 28%, **8**: 59%. vi. sodium acetate (3.0 equiv), acetic acid, NIS (1.2 equiv), 21 °C, 72 h, 82%. vii. DBU (3.0 equiv), toluene, 100 °C, 48 h, 48%. viii. methyl benzoate (0.4 equiv), heptane, h*ν*, silver nitrate (2.0 equiv), 21 °C, 43 h **11**/**12** 1:1, 31%. ix. potassium carbonate (2.0 equiv), ethanol, 21 °C, 18 h, **13**/**14** 1:1, 43%.

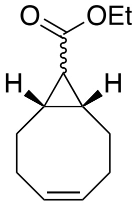
Ethyl (1*R*,4*Z*,8*S*,9*r*)-bicyclo[6.1.0]non-4-ene-9-carboxylate and ethyl (1*R*,4*Z*,8*S*,9*s*)-bicyclo[6.1.0]non-4-ene-9-carboxylate (2): A flask was charged with 1,5 cyclooctadiene (265 mL, 2.16 mol) and copper(II) acetylacetonate (7.05 g, 27 mmol). The mixture was stirred and purged with argon for 15 minutes. Next, the temperature was elevated to 90 °C followed by the dropwise addition of ethyl diazoacetate (45.4 mL, 82 Wt. %, 360 mmol) and the reaction mixture was stirred for 18 h before it was cooled to 21 °C and the unreacted 1,5-cyclooctadiene was removed by vacuum distillation at 40 °C and a high vacuum (max 10 mbar). To the resulting liquid was added a solution of brine (125 mL) and aqueous ammonium hydroxide (14 mL, 25 Wt. %, 90 mmol). The aqueous layers were back extracted with diethyl ether (2 × 250 mL). Subsequently, the organic layer was washed with ammonium hydroxide (5.5 mL, 25 Wt. %, 36 mmol) in brine (135 mL), an aqueous saturated Na_4EDTA solution (125 mL), dried with $MgSO_4$ and concentrated to afford the product as a mixture of the *exo*- and *endo* diastereomers **2** (42.6 g, 61%) as a yellow oil in a 2:1 ratio respectively. The diastereomers could subsequently be separated with silica gel column chromatography (1:19 EtOAc in pentane) to afford both the pure diastereomers. Spectral data were in accordance with previous reported.[1]

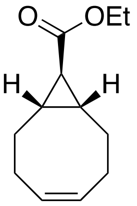
2 (*exo*): **TLC** (EtOAc/heptane, 1:19 v/v): R_F = 0.30. **^{1}H NMR** (500 MHz, $CDCl_3$): δ 5.66 (ddd, *J* = 5.5, 4.0, 1.4 Hz, 2H), 4.12 (q, *J* = 7.1 Hz, 2H), 2.38–2.28 (m, 2H), 2.26–2.17 (m, 2H), 2.15–2.05 (m, 2H), 1.62–1.55 (m, 2H), 1.54–1.45 (m, 1H), 1.27 (t, *J* = 7.1 Hz, 3H), 1.20 (t, *J* = 4.6 Hz, 1H). **^{13}C NMR** (126 MHz, $CDCl_3$): δ 174.58, 130.07, 60.39, 28.42, 28.04, 27.88, 26.81, 14.45.

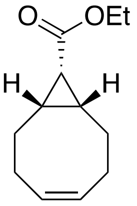
2 (*endo*): **TLC** (EtOAc/heptane, 1:19 v/v): R_F = 0.40. **^{1}H NMR** (500 MHz, $CDCl_3$): δ 5.63 (ddt, *J* = 5.4, 3.6, 0.9 Hz, 2H), 4.13 (q, *J* = 7.1 Hz, 2H), 2.62–2.43 (m, 2H), 2.30–2.16

(m, 2H), 2.14–2.01 (m, 2H), 1.92–1.80 (m, 1H), 1.72 (t, *J* = 8.8 Hz, 1H), 1.50–1.35 (m, 2H), 1.28 (t, *J* = 7.1 Hz, 3H). **^{13}C NMR** (126 MHz, $CDCl_3$): δ 172.43, 129.59, 59.86, 27.22, 24.32, 22.81, 21.39, 14.56.

b. *exo*-TCO compounds

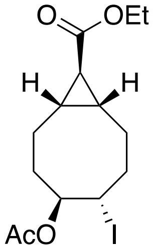

Ethyl (1*S*,4*S*,5*S*,8*R*,9*R*)-4-acetoxy-5-iodobicyclo[6.1.0]nonane-9-carboxylate (3): A suspension of ethyl (1*R*,4*Z*,8*S*,9*r*)-bicyclo[6.1.0]non-4-ene-9-carboxylate (**2** (*exo*), 20.4 g, 105 mmol) and sodium acetate (25.8 g, 315 mmol) in acetic acid (240 mL) was stirred and cooled with a water bath until the temperature stabilized to ambient temperature. Next, NIS (28.4 g, 126 mmol) was added and the mixture was stirred at ambient temperature for 3 h. After full conversion was observed on TLC, brine (50 mL) and heptane (200 mL) were added to the reaction mixture. The organic layer was collected and washed with brine (250 mL), a mixture of sat. aqueous sodium thiosulfate solution (50 mL) and brine (200 mL) followed by sat. aqueous sodium bicarbonate solution (200 mL). The aqueous layers were back-extracted in similar order with two additional portions of heptane (2 × 200 mL). The combined organic layers were dried with $MgSO_4$ and concentrated to afford the crude product (39 g). The crude product was purified with silica gel column chromatography (25 → 75% EtOAc in heptane) to afford **3** (33.2 g, 83%) as white crystals. **TLC** (EtOAc/heptane, 1:3 v/v): R_F = 0.14. **^{1}H NMR** (400 MHz, $CDCl_3$) δ 4.86 (ddd, *J* = 7.7, 6.6, 3.5 Hz, 1H), 4.75 (td, *J* = 7.8, 5.3 Hz, 1H), 4.08 (q, *J* = 7.2 Hz, 2H), 2.24–2.17 (m, 2H), 2.15–2.08 (m, 1H), 2.07 (s, 3H), 2.05–1.96 (m, 2H), 1.84–1.70 (m, 1H), 1.54–1.46 (m, 1H), 1.46–1.38 (m, 1H), 1.34–1.25 (m, 2H), 1.23 (t, *J* = 7.1 Hz, 3H), 1.15 (t, *J* = 4.3 Hz, 1H). **^{13}C NMR** (101 MHz, $CDCl_3$) δ 173.6, 169.7, 73.3, 60.4, 37.5, 33.7, 33.2, 26.1, 26.1, 26.0, 25.7, 23.6, 21.4, 14.2.

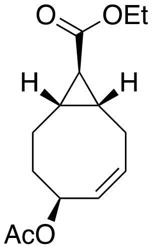

Ethyl (1*R*,3*Z*,5*S*,8*S*,9*R*)-5-acetoxybicyclo[6.1.0]non-3-ene-9-carboxylate (4): DBU (39.5 mL, 262 mmol) was added to a stirred solution of ethyl 4-acetoxy-5-iodobicyclo[6.1.0]nonane-9-carboxylate (**3**, 33.2 g, 87.3 mmol) in toluene (279 mL, 2.62 mol). The mixture was stirred at 100 °C for 18 h. The reaction mixture was cooled with an ice bath and filtered to remove solids. The filter residue was washed with toluene (2 × 50 mL). The filtrate was washed with brine (2 × 250 mL) and the aqueous layers were back extracted with

toluene (100 mL). The combined organic layers were dried with $MgSO_4$ and concentrated to afford a yellow oil as the crude product (25 g). The organic layer was concentrated *in vacuo* and purified with silica gel column chromatography (10 → 20% EtOAc in heptane) to afford **4** (19.6 g, 89%). **TLC** (EtOAc/heptane, 1:4 v/v): R_F = 0.26. **^{1}H NMR** (500 MHz, $CDCl_3$) δ 5.81 (dddd, *J* = 11.3, 9.2, 6.9, 2.0 Hz, 1H), 5.62 (dd, *J* = 11.1, 5.3 Hz, 1H), 5.32 (dd, *J* = 10.6, 5.2 Hz, 1H), 4.10 (q, *J* = 7.2 Hz, 2H), 2.44–2.34 (m, 2H), 2.05 (s, 3H), 1.91–1.85 (m, 1H), 1.79–1.63 (m, 3H), 1.44–1.28 (m, 3H), 1.25 (t, *J* = 7.1 Hz, 3H). **^{13}C NMR** (126 MHz, $CDCl_3$) δ 173.5, 170.1, 133.9, 129.1, 75.3, 60.3, 36.4, 31.2, 28.6, 26.9, 26.4, 25.4, 21.3, 14.2. **HRMS** (m/z): $[M + Na]^+$ calcd. for $C_{14}H_{20}O_4Na$: 275.1259, found 275.1240.

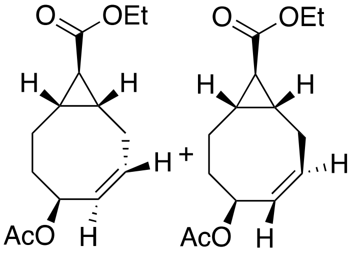

Ethyl (1*R*,3*E*,5*S*,8*S*,9*R*,*P*)-5-acetoxybicyclo[6.1.0]non-3-ene-9-carboxylate (5) and ethyl (1*R*,3*E*,5*S*,8*S*,9*R*,*M*)-5-acetoxybicyclo[6.1.0]non-3-ene-9-carboxylate (6): A custom-made long-necked flask was charged with an aqueous solution of silver nitrate (7.00 g, 41.2 mmol) in water (100 mL). Next, a solution of ethyl (1*R*,3*Z*,5*S*,8*S*,9*R*)-5-acetoxybicyclo[6.1.0]non-3-ene-9-carboxylate (**4**, 3.50 g, 150 mmolar, 13.9 mmol) and methyl benzoate (3.84 g, 3.56 mL, 28.2 mmol) in deoxygenated heptane (160 mL) and MTBE (40 mL) was loaded into a UV irradiation setup (as described in Blanco-Ania *et al.*)[2] The continuous process ran for 16 h. Next, the biphasic reaction mixture was loaded into a separation funnel and the aqueous layer was collected. The organic layer was washed with water (100 mL). The combined aqueous layers were washed with heptane (100 mL). Subsequently, 25% aqueous ammonium hydroxide (25 mL) was added to the aqueous layer before extracting it with EtOAc (100 mL). The combined organic layers were dried with $MgSO_4$ and concentrated to afford a diastereomeric mixture with a ratio of the axial and equatorial isomers 7:13 (**5**/**6**) as a clear oil (1.6 g, 46%). **HRMS** (m/z): $[M + Na]^+$ calcd. for $C_{14}H_{20}O_4Na$: 275.1259, found 275.1264.

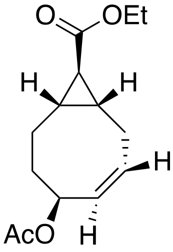

5 (axial): **TLC** (EtOAc/heptane, 1:1 v/v): R_F = 0.54. **^{1}H NMR** (500 MHz, $CDCl_3$) δ 6.11 (ddd, *J* = 16.8, 10.9, 5.9 Hz, 1H), 5.55 (dd, *J* = 16.9, 3.4 Hz, 1H), 5.12 (q, *J* = 3.1 Hz, 1H), 4.14–4.05 (m, 2H), 2.67 (dt, *J* = 12.6, 6.0 Hz, 1H), 2.51–2.45 (m, 1H), 2.25–2.20 (m, 1H), 2.07 (m, 1H), 2.05 (s, 3H), 2.00–1.95 (m, 1H), 1.80–1.75 (m, 1H), 1.60–1.55 (m, 2H), 1.50–1.45 (m, 1H), 1.25 (m, 3H). **^{13}C NMR** (126 MHz, $CDCl_3$) δ 174.9, 170.0, 131.7, 129.5, 70.7, 60.5, 37.2, 31.3, 29.2, 26.7, 21.1, 20.6, 14.2.

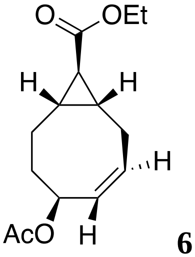

6 (equatorial): **TLC** (EtOAc/heptane, 1:1 v/v): R_F = 0.54. **^{1}H NMR** (500 MHz, $CDCl_3$) δ 5.91 (ddd, *J* = 16.0, 9.1, 6.4 Hz, 1H), 5.67 (ddd, *J* = 16.6, 9.7, 1.5 Hz, 1H), 5.01 (td, *J* = 9.8, 5.3 Hz, 1H), 4.14–4.05 (m, 2H), 2.86 (dtt, *J* = 14.7, 9.2, 1.1 Hz, 1H), 2.40–2.35 (m, 1H), 2.38–2.30 (m, 2H), 2.18 (m, 2H), 2.06 (s, 3H) 1.36 (t, *J* = 5.6 Hz, 1H), 1.25 (m, 3H), 1.25–1.20 (m, 1H), 0.77 (dt, *J* = 15.4, 11.4 Hz, 1H). **^{13}C NMR** (126 MHz, $CDCl_3$) δ 174.2, 170.6, 136.1, 131.2, 77.2, 60.4, 37.6, 36.6, 34.6, 30.2, 29.0, 28.4, 21.2, 14.2.

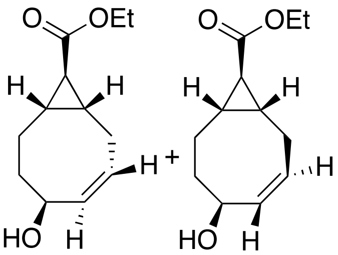

Ethyl (1*R*,3*E*,5*S*,8*S*,9*R*,*P*)-5-hydroxybicyclo[6.1.0]non-3-ene-9-carboxylate (7) and ethyl (1*R*,3*E*,5*S*,8*S*,9*R*,*M*)-5-hydroxybicyclo[6.1.0]non-3-ene-9-carboxylate (8): A solution of *P*- and *M*-isomers of ethyl (1*R*,3*E*,5*S*,8*S*,9*R*)-5-acetoxybicyclo[6.1.0]non-3-ene-9-carboxylate (**5** and **6**, 2.00 g, 7.93 mmol) and potassium carbonate (2.19 g, 15.9 mmol) in ethanol (19.9 mL, 341 mmol) was stirred at 21 °C for 18 h. The flask was shielded from light with aluminum foil. After completion of the reaction was observed with TLC, acetic acid (1.91 mL, 33.3 mmol) in a 1:1 water-brine mixture (50 mL)

was added to quench the reaction. Then, EtOAc (25 mL) was added and separated from the aqueous layer, the organic layer was washed with aqueous sat. $NaHCO_3$ (25 mL) and then with brine (25 mL). All aqueous layers were back-extracted with EtOAc (2 × 25 mL). The combined organic layers were dried with $MgSO_4$, concentrated *in vacuo* and purified with silica gel column chromatography (10% acetone in toluene) to afford **7** (470 mg, 28%) and **8** (980 mg, 59%).

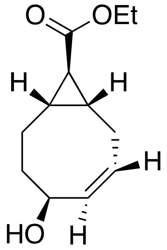

7 (axial): **TLC** (EtOAc/heptane, 1:1 v/v): R_F = 0.37. **^{1}H NMR** (500 MHz, $CDCl_3$) δ 6.26 (ddd, *J* = 16.8, 10.9, 5.9 Hz, 1H), 5.55 (ddd, *J* = 16.8, 3.1, 0.9 Hz, 1H), 4.36 (q, *J* = 3.0 Hz, 1H), 4.08 (qd, *J* = 7.1, 2.1 Hz, 2H), 2.66 (dt, *J* = 12.5, 6.0 Hz, 1H), 2.49–2.42 (m, 1H), 2.25 (dd, *J* = 16.1, 11.9 Hz, 1H), 2.00–1.91 (m, 1H), 1.87 (q, *J* = 8.0 Hz, 1H), 1.79–1.74 (m, 1H), 1.68 (t, *J* = 13.1 Hz, 1H), 1.55– 1.48 (m, 2H), 1.46–1.40 (m, 1H), 1.24 (t, *J* = 7.2 Hz, 3H). **^{13}C NMR** (126 MHz, $CDCl_3$) δ 175.2, 133.5, 130.4, 68.5, 60.4, 39.7, 31.7, 29.1, 27.0, 20.5, 19.3, 14.2.

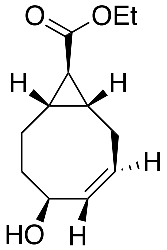

8 (equatorial): TLC (EtOAc/heptane, 1:1 v/v): R_F = 0.27. **^{1}H NMR** (500 MHz, $CDCl_3$) δ 5.76 (ddd, *J* = 15.8, 8.9, 6.5 Hz, 1H), 5.62 (ddd, *J* = 16.5, 9.4, 1.4 Hz, 1H), 4.08 (dd, *J* = 9.8, 5.2 Hz, 1H), 4.05 (q, *J* = 7.1 Hz, 2H), 2.81 (dtt, *J* = 14.8, 9.3, 1.2 Hz, 1H), 2.34–2.29 (m, 1H), 2.28–2.23 (m, 1H), 2.16–2.08 (m, 1H), 2.10–2.05 (m, 1H), 1.43 (dddd, *J* = 13.1, 11.4, 9.7, 0.8 Hz, 1H), 1.32–1.29 (m, 1H), 1.20 (t, *J* = 7.1 Hz, 3H), 1.18–1.13 (m,1H), 0.68 (dt, *J* = 15.4, 11.3 Hz, 1H). **^{13}C NMR** (126 MHz, $CDCl_3$) δ 174.5, 140.3, 129.3, 76.1, 60.4, 38.0, 37.7, 30.3, 29.3, 28.6, 28.5, 14.2.

c. ***endo*-TCO compounds**

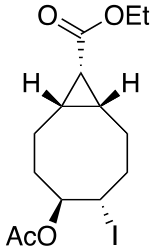

Ethyl (1*S*,4*S*,5*S*,8*R*,9*S*)-4-acetoxy-5-iodobicyclo[6.1.0]nonane-9- carboxylate (9) Ethyl (1*R*,4*Z*,8*S*,9*s*)-bicyclo[6.1.0]non-4-ene-9-carboxylate (**2** (*endo*), 501 mg, 2.6 mmol) and NIS (696 mg, 3.1 mmol) were dissolved in acetic acid (3.0 mL) under an inert atmosphere. To the solution was added 3.0 mL of a saturated NaOAc in AcOH solution. The reaction mixture was stirred for 72 h after which it was diluted with ethyl acetate (20 mL), water (15 mL), brine (15 mL) and 10% aqueous $Na_2S_2O_3$ solution (10 mL). The organic phase was separated from the aqueous layers and the aqueous layers were extracted with ethyl acetate (5x 40 mL). The combined organic layers were dried with $MgSO_4$, concentrated *in vacuo* and purified with silica gel column chromatography (0 → 20% EtOAc in heptane) to afford **9** (800 mg, 82%) as a yellow dense liquid. **TLC** (EtOAc/heptane, 3:7 v/v): R_F = 0.55. **^{1}H NMR** (500 MHz, $CDCl_3$) δ 4.95 (ddd, *J* = 7.8, 6.8, 3.2 Hz, 1H), 4.76 (ddd, *J* = 9.3, 7.8, 3.8 Hz, 1H), 4.14 (q, *J* = 7.1 Hz, 2H), 2.40 – 2.23 (m, 2H), 2.22–2.08 (m, 6H), 1.91–1.64 (m, 4H), 1.42 (dtd, *J* = 10.9, 8.7, 4.0 Hz, 1H), 1.35–1.21 (m, 4H). **^{13}C NMR** (126 MHz, $CDCl_3$) δ 171.8, 169.8, 74.0, 60.2, 38.2, 33.9, 33.9, 23.6, 23.5, 21.7, 21.3, 21.3, 18.7, 14.5.

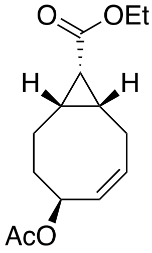

Ethyl (1*R*,3*Z*,5*S*,8*S*,9*S*)-5-acetoxybicyclo[6.1.0]non-3-ene-9- carboxylate (10): Ethyl (1*S*,4*S*,5*S*,8*R*,9*S*)-4-acetoxy-5-iodobicyclo[6.1.0]nonane-9-carboxylate (**9**, 800 mg, 2.1 mmol) was dissolved in dry toluene (10 mL). DBU (1 mL, 6.3 mmol) was added to the solution and the solution was heated to 100 °C. The reaction was stirred at 100 °C for a total of 48 h. The reaction was cooled to 21 °C, washed with water (10 mL), 1 M HCl (10 mL) and brine (10 mL). Subsequently the combined aqueous layers were extracted with toluene (10 mL) and the combined organic layers were dried with $MgSO_4$, concentrated *in vacuo* and purified with silica gel column chromatography (0 → 30% EtOAc in heptane) to afford **10** (254 mg, 48%) as a colorless oil. **TLC** (EtOAc/heptane, 3:7 v/v): R_F = 0.66. **^{1}H NMR** (500 MHz, $CDCl_3$) δ 5.82 (dddd, *J* = 11.1, 9.8, 7.0, 2.1 Hz, 1H), 5.57 (ddd, *J* = 10.9, 5.2, 1.2 Hz, 1H), 5.48–5.39 (m, 1H),

4.12 (q, J = 7.1 Hz, 2H), 2.60 (dddd, J = 13.7, 12.7, 9.8, 1.3 Hz, 1H), 2.29–2.14 (m, 1H), 2.11–2.02 (m, 4H), 1.98–1.90 (m, 2H), 1.76–1.64 (m, 2H), 1.45 (dtd, J = 12.2, 8.7, 3.3 Hz, 1H), 1.34–1.22 (m, 4H). **^{13}C NMR** (126 MHz, $CDCl_3$) δ 172.0, 170.2, 134.0, 130.2, 75.6, 60.1, 36.6, 28.5, 23.3, 22.2, 21.5, 20.6, 14.5. **HRMS** (m/z): $[M + Na]^+$ calcd. for $C_{14}H_{20}O_4Na$: 275.1259, found 275.1243.

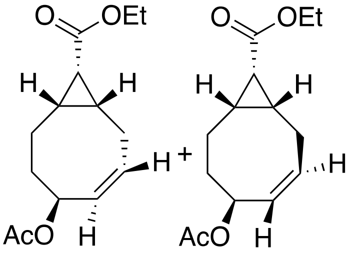

Ethyl (1*R*,3*E*,5*S*,8*S*,9*S*,*P*)-5-acetoxybicyclo[6.1.0]non-3-ene-9-carboxylate (11) and ethyl (1*R*,3*E*,5*S*,8*S*,9*S*,*M*)-5-acetoxybicyclo[6.1.0]non-3-ene-9-carboxylate (12): A custom-made long-necked flask was charged with an aqueous solution of silver nitrate (2.69 g, 15.85 mmol) in water (3 mL). Next, a solution of ethyl (1*R*,3*Z*,5*S*,8*S*,9*S*)-5-acetoxybicyclo[6.1.0]non-3-ene-9-carboxylate (**10**, 2.00 g, 40 mmolar, 7.93 mmol) and methyl benzoate (400 µL, 3.17 mmol) in deoxygenated heptane (20 mL) was loaded into a UV irradiation setup (as described in Blanco-Ania *et al.*)[2] The continuous process ran for 43 h. The extraction vial was disconnected from the setup and residual solution in the photoreactor was collected by flushing the remaining system with heptane (150 mL). Next, the biphasic reaction mixture was loaded into a separation funnel and additional water (150 mL) was added before starting the extraction. After phase separation, the organic layer was washed with a solution of silver nitrate (1.5 g) in water (100 mL). The combined aqueous layers were then back extracted with heptane (100 mL) to remove residual starting material from the aqueous layers. Next, ammonium hydroxide (5.6 mL, 25 Wt. %, 35.67 mmol) was added to the aqueous layer to decomplex the product from the silver ions. The turbid aqueous solution was extracted with ethyl acetate (3 × 200 mL). The combined organic layers were washed with water (100 mL), dried with $MgSO_4$ and concentrated to afford a diastereomeric mixture of the axial and equatorial isomers with a ratio of 1:1 (**11**/**12**) as clear oil (610 mg, 31%). **HRMS** (m/z): $[M + Na]^+$ calcd. for $C_{14}H_{20}O_4Na$: 275.1259, found 275.1241.

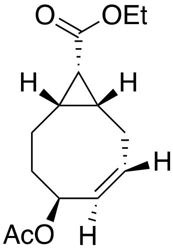

11 (axial): TLC (EtOAc/heptane, 1:1 v/v): R_F = 0.73. **^{1}H NMR** (500 MHz, $CDCl_3$) δ 5.86–5.80 (m, 2H), 5.06–5.00 (m, 1H), 4.16–4.09 (m, 2H), 2.68–2.60 (m, 1H), 2.51 (ddd, *J* = 11.7, 9.2, 1.8 Hz, 1H), 2.48–2.43 (m, 1H), 2.07 (s, 3H), 1.81 (t, *J* = 9.0 Hz, 1H), 1.74–1.71 (m, 2H), 1.62–1.59 (m, 1H), 1.58–1.54 (m, 1H), 1.29–1.25 (m, 3H), 1.25–1.22 (m, 1H). **^{13}C NMR** (126 MHz, $CDCl_3$) δ 171.7, 170.7, 134.0, 131.3, 69.9, 60.1, 38.8, 26.1, 26.0, 24.2, 22.0, 21.4, 17.4, 14.5.

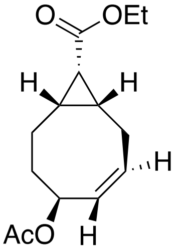

12 (equatorial): **TLC** (EtOAc/heptane, 1:1 v/v): R_F = 0.73. **^{1}H NMR** (500 MHz, $CDCl_3$) δ 6.32 (dddd, *J* = 16.8, 8.4, 6.8, 0.8 Hz, 1H), 5.62 (ddd, *J* = 16.8, 9.7, 1.5 Hz, 1H), 5.15 – 5.07 (m, 1H), 4.16–4.09 (m, 2H), 2.79–2.71 (m, 1H), 2.34 (ddd, *J* = 14.5, 6.7, 2.1 Hz, 1H), 2.23 (dt, *J* = 12.0, 6.2 Hz, 1H), 2.10–2.08 (m, 1H), 2.05 (s, 3H), 1.90–1.84 (m, 1H), 1.73 (d, *J* = 1.4 Hz, 1H), 1.50–1.41 (m, 2H), 1.27 (t, *J* = 0.9 Hz, 3H), 1.15–1.07 (m, 1H). **^{13}C NMR** (126 MHz, $CDCl_3$) δ 171.7, 170.7, 135.3, 134.3, 77.9, 60.1, 35.0, 34.8, 26.1, 25.4, 24.0, 24.0, 21.4, 14.5.

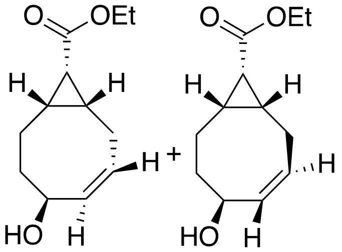

Ethyl (1*R*,3*E*,5*S*,8*S*,9*S*,*P*)-5-hydroxybicyclo[6.1.0]non-3-ene-9-carboxylate (13) and ethyl (1*R*,3*E*,5*S*,8*S*,9*S*,*M*)-5-hydroxybicyclo[6.1.0]non-3-ene-9-carboxylate (14): A solution of *P*- and *M*-isomers of ethyl (1*R*,3*E*,5*S*,8*S*,9*S*)-5-acetoxybicyclo[6.1.0]non-3-ene-9-carboxylate (**11** and **12**, 396 mg, 1.57 mmol) and potassium carbonate (434 mg, 3.14 mmol) in ethanol (5 mL, 67.5 mmol) was stirred at 21 °C for 18 h. The

flask was shielded from light with aluminum foil. LCMS indicated still a significant amount of starting material so the temperature was raised to 30 °C and the reaction was stirred for 4 h. Hereafter, acetic acid (377 µL, 6.59 mmol) and EtOAc (50 mL) were added to the reaction and the organic layer was separated from the aqueous layer. The organic layer was washed with aqueous sat. $NaHCO_3$ (25 mL) and brine (25 mL) and the combined organic layers were dried with $MgSO_4$, concentrated *in vacuo* and purified with silica gel column chromatography (40% EtOAc in pentane) to afford a diastereomeric mixture with a ratio of 1:1 (**13**/**14**) of two products as a clear oil (141 mg, 43%).

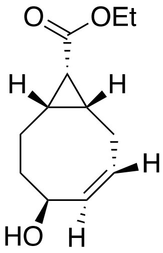

13 (axial): TLC (EtOAc/pentane, 1:1 v/v): R_F = 0.42. **^{1}H NMR** (400 MHz, $CDCl_3$) δ 5.98–5.82 (m, 2H), 4.29 (td, *J* = 7.5, 4.2 Hz, 1H), 4.16–4.09 (m, 2H), 2.68–2.61 (m, 1H), 2.53 (dd, *J* = 8.7, 3.1 Hz, 1H), 2.49–2.43 (m, 1H), 1.83–1.78 (m, 1H), 1.75–1.70 (m, 2H), 1.63–1.55 (m, 2H), 1.29–1.25 (m, 3H), 1.24–1.22 (m, 1H). **^{13}C NMR** (101 MHz, $CDCl_3$) δ 171.6, 133.8, 132.6, 67.5, 60.1, 38.4, 26.3, 26.1, 24.6, 23.8, 21.9, 21.1, 17.5, 14.5.

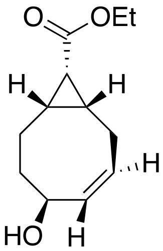

14 (equatorial): TLC (EtOAc/pentane, 1:1 v/v): R_F = 0.42. **^{1}H NMR** (400 MHz, $CDCl_3$) δ 6.22 (dddd, *J* = 16.8, 8.3, 6.6, 0.8 Hz, 1H), 5.61 (ddd, *J* = 16.8, 9.5, 1.6 Hz, 1H), 4.25–4.18 (m, 1H), 4.16–4.08 (m, 2H), 2.79–2.69 (m, 1H), 2.31 (ddd, *J* = 14.5, 6.7, 2.1 Hz, 1H), 2.21–2.13 (m, 1H), 2.12–2.06 (m, 1H), 1.88–1.82 (m, 1H), 1.73–1.68 (m, 1H), 1.50–1.41 (m, 2H), 1.29–1.25 (m, 3H), 1.16–1.07 (m, 1H). **^{13}C NMR** (101 MHz, $CDCl_3$) δ 171.6, 138.4, 135.7, 76.8, 60.1, 34.9, 32.7, 26.3, 25.4, 24.1, 23.9, 21.1, 14.5.

d. *exo* axial derivatives

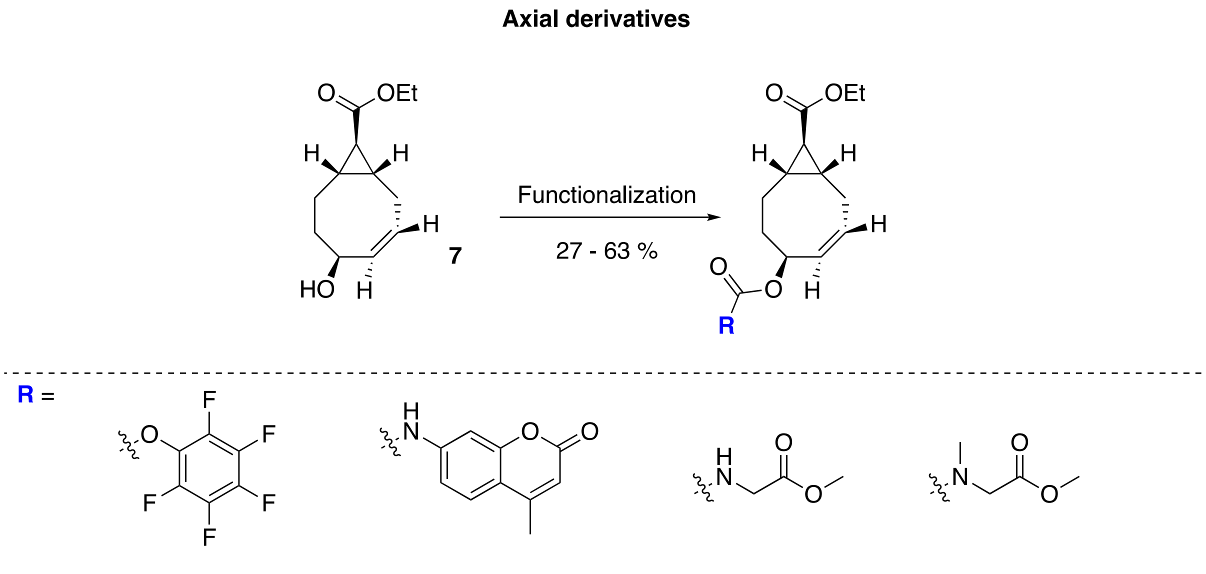

15 **21** **23** **25**

Scheme S2: 15. DIPEA (3.0 equiv), DPFPC (2.75 equiv), DCM, 21 °C, 18 h, 32%. **21**. 7-amino-4-methylcoumarin (1.25 equiv), triphosgene (1.25 equiv), DIPEA (4.6 equiv), DMAP (3.0 equiv), toluene, DCM, 21 °C, 18 h 63%. **23**. i. DPFPC (2.0 equiv), DIPEA (1.0 equiv), DMAP (0.1 equiv), MeCN. ii. glycine methyl ester hydrochloride (2.0 equiv), DIPEA (2.5 equiv), MeCN, 21 °C, 18 h, 27%. **25**. i. DPFPC (2.0 equiv), DIPEA (5.0 equiv), DMAP (0.1 equiv), MeCN. ii. sarcosine methyl ester hydrochloride (2.0 equiv), DIPEA (2.5 equiv), MeCN, 21 °C, 18 h 31%.

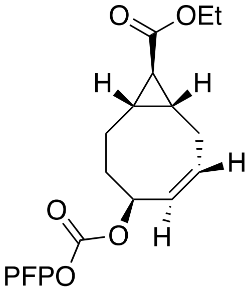

COOEt-*exo*-axial-TCO-PFP (15): DPFPC (196 mg, 497 µmol) was added at 0 °C to a stirred solution of ethyl (1*R*,3*E*,5*S*,8*S*,9*R*,*P*)-5-hydroxybicyclo[6.1.0]non-3-ene-9-carboxylate (**7**, 38.0 mg, 181 µmol) and DIPEA (94 µL, 542 µmol) in DCM (988 µL, 15.4 mmol). After the addition was complete, the mixture was covered from light and allowed to warm up to ambient temperature. After completion, water (1 mL) was added and subsequently the mixture was neutralized with acetic acid. The organic layer was washed with water (2 mL). The organic layer was dried with $MgSO_4$, concentrated *in vacuo* and purified with silica gel column chromatography (10 → 50% EtOAc in heptane) to afford **15** (24 mg, 32%). **TLC**

(EtOAc/heptane, 1:1 v/v): R_F = 0.55. **^{1}H NMR** (400 MHz, $CDCl_3$) δ 6.27 (ddd, *J* = 16.9, 10.9, 5.9 Hz, 1H), 5.57 (dd, *J* = 16.9, 3.3 Hz,1H), 5.17 (d, *J* = 3.1 Hz, 1H), 4.13 (q, *J* = 7.2 Hz, 2H), 2.76 (dt, *J* = 12.8, 6.3 Hz, 1H), 2.57–2.47 (m, 1H), 2.34–2.19 (m, 2H), 2.08 (s, 1H), 1.88 (t, *J* = 13.9 Hz, 1H), 1.64–1.54 (m, 2H), 1.53–1.46 (m, 1H), 1.26 (t, *J* = 7.1 Hz, 3H). **^{13}C NMR** (101 MHz, $CDCl_3$) δ 174.7, 150.3, 142.4, 141.0, 140.1, 139.1, 138.4, 136.6, 133.2, 127.5, 77.1, 60.6, 37.3, 31.2, 29.4, 26.4, 20.4, 14.1. **^{19}F NMR** (377 MHz, $CDCl_3$) δ −153.22 (d, *J* = 17.0 Hz, 2F), −157.50 (t, *J* = 21.7 Hz, 1F), −162.04 (dd, *J* = 21.8, 17.0 Hz, 2F).

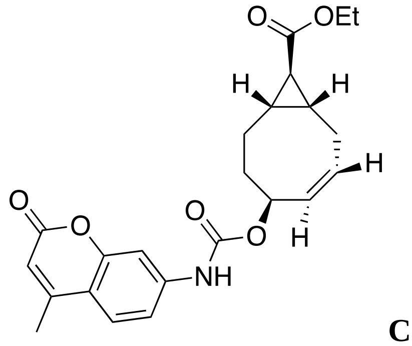

COOEt-*exo*-axial-TCO-AMC (21): DIPEA (442 μL, 2.54 mmol) and triphosgene (205 mg, 0.69 mmol) were added to a solution of 7-amino-4-methylcoumarin (121 mg, 0.69 mmol) in dry toluene (15 mL). The reaction mixture was stirred at 120 °C for 1 h and allowed to cool down to 21 °C. Ethyl (1*R*,3*E*,5*S*,8*S*,9*R*,*P*)-5-hydroxybicyclo[6.1.0]non-3-ene-9-carboxylate (**7**, 116 mg, 0.52 mmol) was dissolved in dry DCM (10 mL) and DMAP (202 mg, 1.66 mmol) was added. The cooled 7-amino-4-methylcoumarin solution was added dropwise to the TCO mixture in DCM on ice. The reaction mixture was stirred for 18 h in the dark. The reaction mixture was concentrated and suspended in EtOAc (80 mL). A solution of 1 M aqueous HCl (40 mL) was added and the aqueous phase was separated. The organic layer was washed with brine (30 mL). The combined aqueous layers were back extracted with EtOAc (2 × 20 mL). The combined organic layers were dried with $MgSO_4$, concentrated *in vacuo* and purified with silica gel column chromatography (5 → 50% EtOAc in pentane) to afford **21** (142.3 mg, 63%) as an off-white solid. **TLC** (EtOAc/pentane, 1:1 v/v): R_F = 0.54. **^{1}H NMR** (500 MHz, $CDCl_3$) δ 7.55 (d, *J* = 8.6 Hz, 1H), 7.48–7.43 (m, 2H), 7.15 (s, 1H), 6.25–6.16 (m, 1H), 5.63 (dd, *J* = 16.9, 3.3 Hz, 1H), 5.23 (d, *J* = 3.2 Hz, 1H), 4.17–4.11 (m, 2H), 2.72 (dt, *J* = 12.5, 6.0 Hz, 1H), 2.55–2.48 (m, 1H), 2.43 (d, *J* = 1.3 Hz, 3H), 2.30 – 2.14 (m, 2H), 2.04 (q, *J* = 6.5 Hz, 1H), 1.86 (t, *J* = 13.6 Hz, 1H), 1.60 (q, *J* = 8.7 Hz, 3H), 1.50 (dd, *J* = 9.1, 6.4 Hz, 1H), 1.30 (t, *J* = 7.1 Hz, 3H). **^{13}C NMR** (126 MHz, $CDCl_3$) δ 175.0, 161.2, 154.5, 152.4, 152.1, 141.6, 132.4, 129.4, 125.5, 115.6, 114.5, 113.3, 106.0, 72.2, 60.7,

37.6, 31.5, 29.5, 26.8, 21.1, 20.8, 18.7, 14.4. **HRMS** (m/z): [M + Na]$^+$ calcd. for $C_{23}H_{25}N_1O_6Na$: 434.1579, found 434.1575.

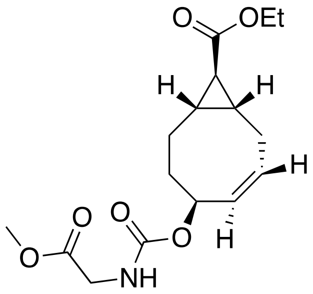

COOEt-*exo*-axial-TCO-glycine-OMe (23): DPFPC (172 mg, 438 µmol), DIPEA (191 µL, 1.09 mmol), and DMAP (2.7 mg, 21.9 µmol) were added at 0 °C to a stirred solution of ethyl (1*R*,3*E*,5*S*,8*S*,9*R*,*P*)-5-hydroxybicyclo[6.1.0]non-3-ene-9-carboxylate (**7**, 46 mg, 219 µmol) in dry acetonitrile (3 mL). After the addition was complete, the mixture was shielded from light and allowed to warm up to ambient temperature for 16 h. After completion, the reaction mixture was diluted with diethyl ether (10 mL) and washed with water (2 × 5 mL). The organic layer was dried with $MgSO_4$ and concentrated *in vacuo*. Without further purification, the product was dissolved in dry acetonitrile (2.5 mL) and to this a solution of glycine methyl ester hydrochloride (55 mg, 438 µmol) and DIPEA (95.4 µL, 547 µmol) in dry acetonitrile (2.5 mL) was added. The reaction was shielded from light. Upon completion, the reaction was diluted with EtOAc (10 mL) and extracted with water (2 × 5 mL). The organic layers were dried with $MgSO_4$, concentrated *in vacuo* and purified with silica gel column chromatography (5% acetone in toluene) to afford **23** (19 mg, 27%) as a yellow oil. **TLC** (acetone/toluene, 1:9 v/v): R_F = 0.21. **^{1}H NMR** (500 MHz, $CDCl_3$) δ 6.13 (ddd, *J* = 16.9, 11.0, 5.9, 1H), 5.56 (dd, *J* = 16.9, 3.3, 1H), 5.22 (t, *J* = 5.6, 1H), 5.08 (q, *J* = 3.1, 1H), 4.11 (qd, *J* = 7.2, 2.6, 2H), 3.97 (dd, *J* = 5.6, 3.1, 2H), 3.76 (s, 3H), 2.67 (dt, *J* = 9.8, 6.0, 1H), 2.46 (t, *J* = 11.9, 1H), 2.24–2.17 (m, 1H), 2.10 (ddd, *J* = 15.2, 6.7, 2.8, 1H), 2.01–1.89 (m, 1H), 1.76 (t, *J* = 13.8, 1H), 1.65–1.50 (m, 2H), 1.48-1.41 (m, 1H), 1.26 (t, *J* = 7.1, 3H). **^{13}C NMR** (126 MHz, $CDCl_3$) δ 175.1, 170.6, 155.5, 131.8, 129.9, 71.8, 60.7, 52.5, 42.7, 31.6, 29.4, 27.1, 20.6, 14.4. **HRMS** (m/z): [M + Na]$^+$ calcd. for $C_{16}H_{23}NO_6Na$: 348.1423, found 348.1413.

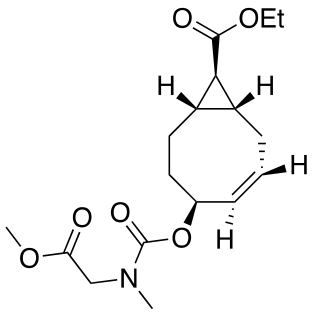

COOEt-*exo*-axial-TCO-sarcosine-OMe (25): DPFPC (191 mg, 485 µmol), DIPEA (208 µL, 1.2 mmol), and DMAP (3.6 mg, 29 µmol) were added at 0 °C to a stirred solution of ethyl (1*R*,3*E*,5*S*,8*S*,9*R*,*P*)-5-hydroxybicyclo[6.1.0]non-3-ene-9-carboxylate (**7**, 50.3 mg, 239 µmol) in dry acetonitrile (3 mL), was added. After the addition was complete, the mixture was shielded from light and allowed to warm up to ambient temperature for 16 h. After completion, the reaction mixture was diluted with diethyl ether (10 mL) and washed with water (2 × 5 mL). The organic layer was dried with $MgSO_4$ and concentrated *in vacuo*. Without further purification, the product was dissolved in dry acetonitrile (2.5 mL) and to this a solution of sarcosine methyl ester hydrochloride (66.4 mg, 476 µmol) and DIPEA (104 µL, 595 µmol) in dry acetonitrile (2.5 mL) was added. The reaction was shielded from light. Upon completion, the reaction mixture was diluted with EtOAc (10 mL) and it was washed with water (2 × 5 mL). The organic layer was dried with $MgSO_4$, concentrated *in vacuo* and purified with silica gel column chromatography (5% acetone in toluene) to afford **25** as a colorless oil (31 mg, 31%). **TLC** (acetone/toluene, 1:9 v/v) R_F = 0.32. **^{1}H NMR** (500 MHz, $CDCl_3$) **rotamer 1:** δ 6.12 (ddd, *J* = 16.8, 10.9, 5.9, 1H), 5.56 (td, *J* = 16.7, 3.3, 1H), 5.11 (dd, *J* = 5.9, 3.0, 1H), 4.11 (dtd, *J* = 10.1, 7.1, 2.8, 2H), 4.06−3.95 (m, 2H), 3.73 (s, 3H), 3.03 (s, 3H), 2.67 (tt, *J* = 12.4, 6.2, 1H), 2.52−2.42 (m, 1H) 2.22 (dd, *J* = 16.2, 11.8, 1H), 2.15-2.10 (m, 1H), 2.10−2.02 (m, 1H), 1.81−1.68 (m, 1H), 1.55 (td, *J* = 11.3, 10.4, 5.8, 2H), 1.45 (ddd, *J* = 15.0, 9.9, 6.1, 1H), 1.26 (td, *J* = 7.1, 2.1, 3H), **rotamer 2:** δ 5.99 (ddd, *J* = 16.8, 10.9, 5.9, 1H), 5.56 (td, *J* = 16.7, 3.3, 1H), 5.11 (dd, *J* = 5.9, 3.0, 1H), 4.11 (dtd, *J* = 10.1, 7.1, 2.8, 2H), 4.06-3.95 (m, 2H), 3.77 (s, 3H), 2.99 (s, 3H), 2.67 (tt, *J* = 12.4, 6.2, 1H), 2.52−2.42 (m, 1H) 2.22 (dd, *J* = 16.2, 11.8, 1H), 2.15−2.10 (m, 1H), 2.10−2.02 (m, 1H), 1.81-1.68 (m, 1H), 1.55 (td, *J* = 11.3, 10.4, 5.8, 2H), 1.45 (ddd, *J* = 15.0, 9.9, 6.1, 1H), 1.26 (td, *J* = 7.1, 2.1, 3H) **^{13}C NMR** (126 MHz, $CDCl_3$) **rotamer 1:** δ 175.1, 170.1, 155.9, 131.5, 130.1, 72.3, 60.7, 52.2, 50.6, 37.6, 35.3, 31.6, 29.4, 26.9, 20.7, 14.4, **rotamer 2:** δ 175.2, 170.2, 155.2, 131.6, 129.9, 72.2, 60.7, 52.3, 50.7, 37.6, 36.2, 31.6, 29.4, 26.9, 20.6, 14.43. **HRMS** (m/z): $[M + Na]^+$ calcd. for $C_{17}H_{25}NO_6Na$: 362.1579, found 362.1598.

e. exo equatorial derivatives

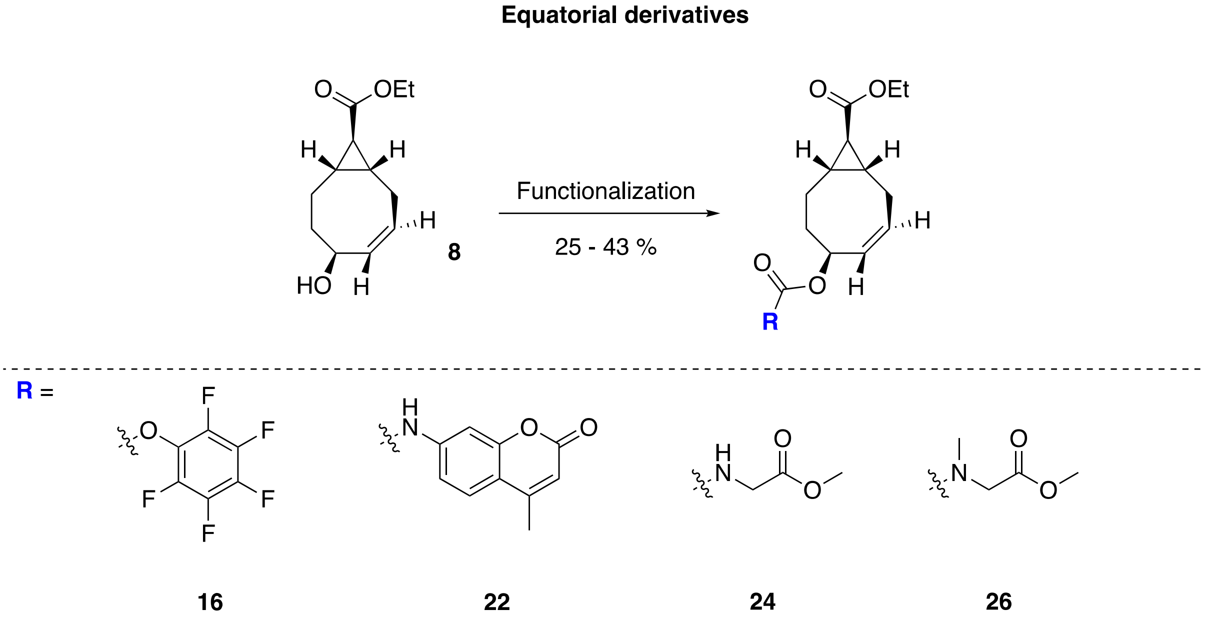

Scheme S3: 16. DIPEA (3.0 equiv), DPFPC (2.75 equiv), DCM, 21 °C, 18 h, 43%. **22**. 7-amino-4-methylcoumarin (1.25 equiv), triphosgene (1.25 equiv), DIPEA (4.6 equiv), DMAP (3.0 equiv), toluene, DCM, 21 °C, 18 h, 42%. **24**. i. DPFPC (2.0 equiv), DIPEA (5.0 equiv), DMAP (5 equiv), MeCN. ii. glycine methyl ester hydrochloride (5.0 equiv), DIPEA (2.5 equiv), MeCN, 21 °C, 18 h, 42%. **26**. i. DPFPC (2.0 equiv), DIPEA (5.0 equiv), DMAP (0.1 equiv), MeCN. ii. sarcosine methyl ester hydrochloride (2.0 equiv), DIPEA (2.5 equiv), MeCN, 21 °C, 18 h, 25%.

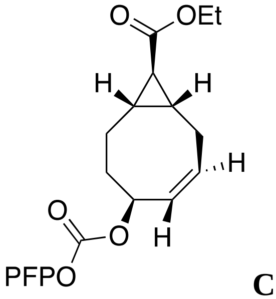

COOEt-*exo*-equatorial-TCO-PFP (16): DPFPC (268 mg, 680 µmol) was added at 0 °C to a stirred solution of ethyl (1*R*,3*E*,5*S*,8*S*,9*R*,*M*)-5-hydroxybicyclo[6.1.0]non-3-ene-9-carboxylate (**8**, 52.0 mg, 247 µmol) and DIPEA (129 µL, 742 µmol) in DCM (1.35 mL, 21 mmol). After the addition was complete, the mixture was covered from light and allowed to warm up to ambient temperature. After completion, water (1 mL) was added and subsequently the mixture was neutralized with acidic acid. The organic layer was washed with water (2 mL). The organic layer was dried with $MgSO_4$, concentrated *in vacuo* and purified with silica gel column chromatography (10 → 50% EtOAc in heptane) to afford **16** (45 mg, 43%). **TLC**

(EtOAc/heptane, 1:1 v/v): R_F = 0.57. **^{1}H NMR** (400 MHz, $CDCl_3$) δ 6.01 (ddd, *J* = 16.0, 9.0, 6.5 Hz, 1H), 5.79 (ddd, *J* = 16.6, 9.6, 1.4 Hz, 1H), 5.03 (td, *J* = 9.9, 5.3 Hz, 1H), 4.10 (q, *J* = 7.1 Hz, 2H), 2.99–2.86 (m, 1H), 2.49–2.31 (m, 3H), 2.26–2.18 (m, 1H), 1.70 (dt, *J* = 12.9, 10.7 Hz, 1H), 1.39 (t, *J* = 5.6 Hz, 1H), 1.30–1.19 (m, 1H), 1.25 (t, *J* = 7.1 Hz, 3H), 0.78 (dt, *J* = 15.1, 11.3 Hz, 1H). **^{13}C NMR** (101 MHz, $CDCl_3$) δ 174.1, 150.6, 142.5, 140.9, 140.1, 139.1, 138.4, 136.4, 134.5, 132.9, 83.5, 60.6, 37.5, 34.3, 30.0, 28.7, 28.7, 28.4, 14.2. **^{19}F NMR** (377 MHz, $CDCl_3$) δ −153.16 (d, *J* = 16.9 Hz), −157.66 (t, *J* = 21.7 Hz), −162.15 (dd, *J* = 21.7, 17.0 Hz).

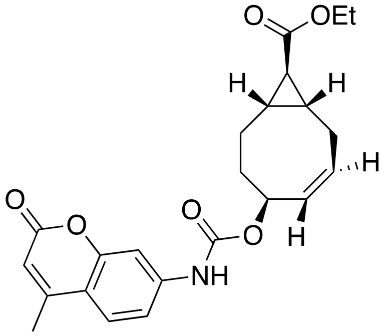

COOEt-*exo*-equatorial-TCO-AMC (22): DIPEA (55 µL, 317 µmol) and triphosgene (25 mg, 86 µmol) were added to a solution of 7-amino-4-methylcoumarin (15 mg, 86 µmol) in dry toluene (2 mL). The reaction mixture was stirred at 120 °C for 1 h and allowed to cool down to 21 °C. Ethyl (1*R*,3*E*,5*S*,8*S*,9*R*,*M*)-5-hydroxybicyclo[6.1.0]non-3-ene-9-carboxylate (**8**, 14 mg, 67 µmol) was dissolved in dry DCM (5 mL) and DMAP (26 mg, 214 µmol) was added. The cooled 7-amino-4-methylcoumarin solution was added dropwise to the TCO mixture in DCM on ice. The reaction mixture was stirred for 18 h in the dark. The reaction mixture was concentrated *in vacuo* and purified with silica gel column chromatography (10 → 60% EtOAc in pentane) to afford **22** (14 mg, 42%) as an off-white solid. **TLC** (EtOAc/pentane, 1:1 v/v): R_F = 0.40. **^{1}H NMR** (500 MHz, DMSO) δ 10.20 (s, 1H), 7.69 (d, *J* = 8.7 Hz, 1H), 7.54 (d, *J* = 2.1 Hz, 1H), 7.41 (dd, *J* = 8.7, 2.1 Hz, 1H), 6.23 (d, *J* = 1.4 Hz, 1H), 6.12 – 6.03 (m, 1H), 5.67 (ddd, *J* = 16.6, 9.7, 1.3 Hz, 1H), 5.02 (td, *J* = 9.9, 5.1 Hz, 1H), 4.01 (q, *J* = 7.0 Hz, 2H), 2.83 (dt, *J* = 14.4, 9.3 Hz, 1H), 2.38 (d, *J* = 1.2 Hz, 3H), 2.32–2.24 (m, 2H), 2.20 (dt, *J* = 12.6, 6.2 Hz, 1H), 2.08–2.00 (m, 1H), 1.56–1.47 (m, 2H), 1.17 (t, *J* = 7.1 Hz, 3H), 1.14–1.07 (m, 1H), 0.94 (q, *J* = 12.1 Hz, 1H). **^{13}C NMR** (126 MHz, DMSO) δ 173.5, 160.0, 153.8, 153.2, 152.7, 142.8, 135.6, 131.8, 114.3, 114.2, 111.8, 104.4, 77.8, 59.9, 37.1, 34.6, 28.5, 28.0, 17.9, 14.1. **HRMS** (m/z): $[M + Na]^+$ calcd. for $C_{23}H_{25}N_1O_6Na$: 434.1579, found 434.1578.

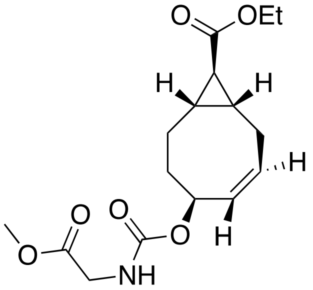

COOEt-*exo*-equatorial-TCO-glycine-OMe (24): A solution of ethyl (1*R*,3*E*,5*S*,8*S*,9*R*,*M*)-5-hydroxybicyclo[6.1.0]non-3-ene-9-carboxylate (**8**, 95 mg, 452 µmol) and DIPEA (393 uL, 2.26 mmol) in DMF (1 mL) was added to a stirred solution of DPFPC (356 mg, 904 µmol) and DMAP (276 mg, 2.26 mmol) in dry DMF (1 mL). After the addition was complete, the mixture was covered from light. After overnight stirring, half of the reaction (1 mL) was transferred into a new, flame dried flask. Glycine methyl ester hydrochloride (30.2 mg, 339 µmol) was added to this solution. After 2.5 h, 2 additional equivalents of glycine methyl ester hydrochloride and 2.5 additional equivalents of DIPEA were added. When the reaction was complete, the reaction mixture was diluted with DCM and washed with 5% $NaHCO_3$ (5 mL), aqueous sat. NH_4Cl (5 mL) and brine (5 mL). The organic layer was dried with $MgSO_4$ and concentrated *in vacuo* and purified with silica gel column chromatography (30% EtOAc in heptane) to afford **24** (20.2 mg, 42 %). **TLC** (EtOAc/heptane, 1:1 v/v) R_F = 0.33. **^{1}H NMR** (500 MHz, $CDCl_3$) δ 5.91 (ddd, *J* = 16.0, 9.2, 6.5 Hz, 1H), 5.67 (ddd, *J* = 16.5, 9.6, 1.5 Hz, 1H), 5.15 (s, 1H), 4.95 (td, *J* = 9.3, 8.7, 4.9, 1H), 4.09 (q, *J* = 7.1, 2H), 3.97 (dd, *J* = 5.6, 2.9, 2H), 3.76 (s, 3H), 2.91-2.81 (m, 1H), 2.40–2.28 (m, 2H), 2.22 (dt, *J* = 12.8, 6.3, 1H), 2.16 (q, *J* = 7.7, 1H), 1.55–1.45 (m, 1H), 1.36 (t, *J* = 5.6, 1H), 1.26 (t, *J* = 7.1, 3H), 1.22–1.17 (m, 1H), 0.82–0.72 (m, 1H). **^{13}C NMR** (126 MHz, $CDCl_3$) δ 174.5, 170.6, 156.0, 136.5, 131.1, 81.1, 60.6, 52.4, 42.6, 37.8, 35.0, 30.3, 29.2, 28.6, 26.5, 14.4. **HRMS** (m/z): $[M + Na]^+$ calcd. for $C_{16}H_{23}NO_6Na$: 348.1423, found 348.1417.

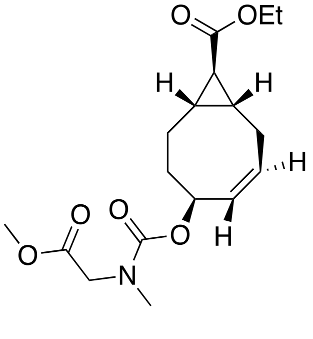

COOEt-*exo*-equatorial-TCO-sarcosine-OMe (26): DPFPC (140 mg, 355 µmol), DIPEA (77.2 µL, 443 µmol), and DMAP (2.17 mg, 18 µmol) were added at 0 °C to a stirred solution of ethyl (1*R*,3*E*,5*S*,8*S*,9*R*,*M*)-5-hydroxybicyclo[6.1.0]non-3-ene-9-carboxylate (**8**, 37 mg, 177 µmol) in dry acetonitrile (1.5 mL). After the addition was complete,

the mixture was shielded from light and allowed to warm up to ambient temperature overnight. After completion, the reaction mixture was diluted with diethyl ether (5 mL) and washed with water (2 × 5 mL). The organic layer was dried with $MgSO_4$ and concentrated *in vacuo*. Without further purification, the product was dissolved in dry acetonitrile (2.5 mL) and to this a solution of sarcosine methyl ester hydrochloride (49.5 mg, 355 µmol) and DIPEA (77.2 µL, 444 µmol) in dry acetonitrile (2.5 mL) was added. The reaction was shielded from light. Upon completion, the reaction mixture was diluted with EtOAc (10 mL) and washed with water (2 × 5 mL). The organic layer was dried with $MgSO_4$, concentrated *in vacuo* and purified with silica gel column chromatography (2 → 5% acetone in toluene) yielding **26** (15.1 mg, 25%) as an oil. **TLC** (acetone/toluene, 1:9 v/v) R_F = 0.34. **^{1}H NMR** (500 MHz, $CDCl_3$) **rotamer 1:** δ 5.96-5.86 (m, 1H), 5.72 (ddd, *J* = 16.5, 9.6, 1.4, 1H), 4.96 (td, *J* = 9.8, 5.3, 1H), 4.09 (q, *J* = 7.3, 2H), 4.05–3.91 (m, 2H), 3.75 (s, 3H), 2.97 (s, 3H), 2.86 (dq, *J* = 17.8, 9.1, 1H), 2.42–2.29 (m, 2H), 2.28–2.20 (m, 1H), 2.20–2.11 (m, 1H), 1.58–1.38 (m, 1H), 1.38–1.32 (m, 1H), 1.24 (t, *J* = 7.2, 3H), 1.22–1.12 (m, 1H), 0.85-0.72 (m, 1H), **rotamer 2:** δ 5.96–5.86 (m, 1H), 5.63 (ddd, *J* = 16.5, 9.7, 1.4, 1H), 4.96 (td, *J* = 9.8, 5.3, 1H), 4.09 (q, *J* = 7.3, 2H), 4.05–3.91 (m, 2H), 3.73 (s, 3H), 2.98 (s, 3H), 2.86 (dq, *J* = 17.8, 9.1, 1H), 2.42–2.29 (m, 2H), 2.28–2.20 (m, 1H), 2.20–2.11 (m, 1H), 1.58–1.38 (m, 1H), 1.38–1.32 (m, 1H), 1.24 (t, *J* = 7.2, 3H), 1.22–1.12 (m, 1H), 0.85–0.72 (m, 1H). **^{13}C NMR** (126 MHz, $CDCl_3$) **rotamer 1:** δ 174.5, 170.2, 155.8, 136.8, 131.6, 78.8, 60.6, 52.2, 50.6, 37.8, 35.3, 35.0, 30.3 29.3, 29.2, 28.6, 14.4, **rotamer 2:** δ 174.5, 170.2, 156.6, 136.6, 131.6, 79.0, 60.6, 52.2, 50.6, 37.8, 35.9, 35.0, 30.3 29.3, 29.2, 28.6, 14.4. **HRMS** (m/z): $[M + Na]^+$ calcd. for $C_{17}H_{25}NO_6Na$: 362.1579, found 362.1565.

f. *endo* derivatives

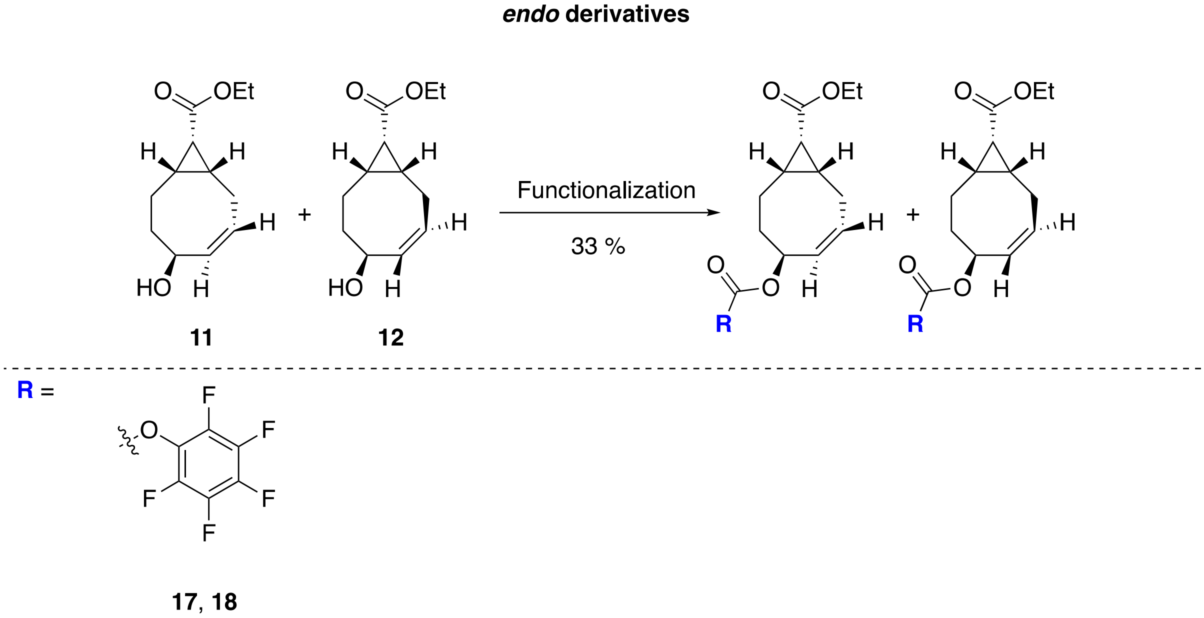

Scheme S4: 17/18. DIPEA (3.0 equiv), DPFPC (2.75 equiv), DCM, 21 °C, 18 h, 33%.

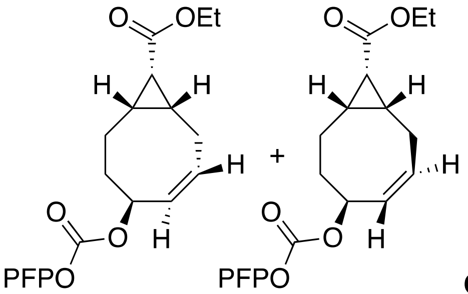

COOEt-*endo*-TCO-PFP (17 and 18) DIPEA (119 µL, 685 µmol) was added to a solution of the diastereomeric mixture **17/18** (48 mg, 228 µmol) in dry DCM (5 mL). The reaction was cooled to 0 °C before DPFPC (247 mg, 628 µmol) was added. After the addition was complete, the mixture was covered from light and allowed to warm up to ambient temperature overnight. After completion, water (10 mL) and DCM (15 mL) were added. The organic layer was separated and washed with brine (5 mL), dried with $MgSO_4$, concentrated *in vacuo* and purified with silica gel column chromatography (0 → 25% EtOAc in pentane) to afford a diastereomeric mixture of the axial and equatorial isomers with a ratio of 2.5:1 (**17/18**) as a clear oil (32 mg, 33%).

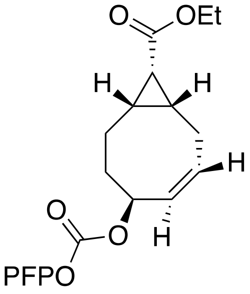

17 (axial): TLC (EtOAc/pentane, 1:1 v/v): R_F = 0.82. **^{1}H NMR** (400 MHz, $CDCl_3$) δ 5.96 (ddd, *J* = 16.9, 11.2, 3.5 Hz, 1H), 5.82 (dd, *J* = 16.8, 4.7 Hz, 1H), 5.12–5.06 (m, 1H), 4.17–4.10 (m, 2H), 2.73–2.64 (m, 1H), 2.62–2.52 (m, 2H), 1.85 (t, *J* = 9.0 Hz, 1H), 1.77–1.74 (m, 2H), 1.64–1.60 (m, 2H), 1.30–1.25 (m, 4H). **^{13}C NMR** (101 MHz, $CDCl_3$) δ 171.6, 150.7, 135.4, 128.9, 76.5, 60.2, 38.4, 31.0, 26.1, 25.6, 21.8, 17.1, 14.5. **^{19}F NMR** (377 MHz, $CDCl_3$) δ -153.07 (d, *J* = 17.0 Hz), -157.75 (t, *J* = 21.7 Hz), -162.02 – -162.27 (m).

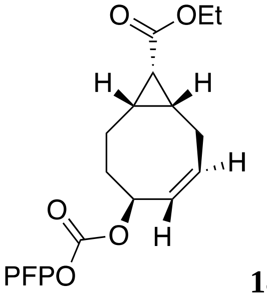

18 (equatorial): TLC (EtOAc/pentane, 1:1 v/v): R_F = 0.82. **^{1}H NMR** (400 MHz, $CDCl_3$) δ 6.47 – 6.37 (m, 1H), 5.73 (ddd, *J* = 16.9, 9.8, 1.5 Hz, 1H), 5.16 – 5.08 (m, 1H), 4.17 – 4.10 (m, 3H), 2.86 – 2.75 (m, 1H), 2.37 (ddd, *J* = 14.7, 6.8, 2.1 Hz, 2H), 2.16 – 2.07 (m, 1H), 1.77 (t, *J* = 9.2 Hz, 1H), 1.44 (dt, *J* = 15.3, 11.7 Hz, 1H), 1.27 (t, *J* = 7.1 Hz, 4H), 1.15 (dtd, *J* = 11.6, 9.1, 4.5 Hz, 1H). **^{13}C NMR** (101 MHz, $CDCl_3$) δ 171.6, 150.7, 137.0, 132.7, 84.1, 60.2, 34.7, 34.6, 25.8, 25.5, 23.9, 23.8, 14.5. **^{19}F NMR** (377 MHz, $CDCl_3$) δ −153.07 (d, *J* = 17.0 Hz), −157.75 (t, *J* = 21.7 Hz), −162.02 to −162.27 (m).

g. Doxorubicin releasing *exo* derivative

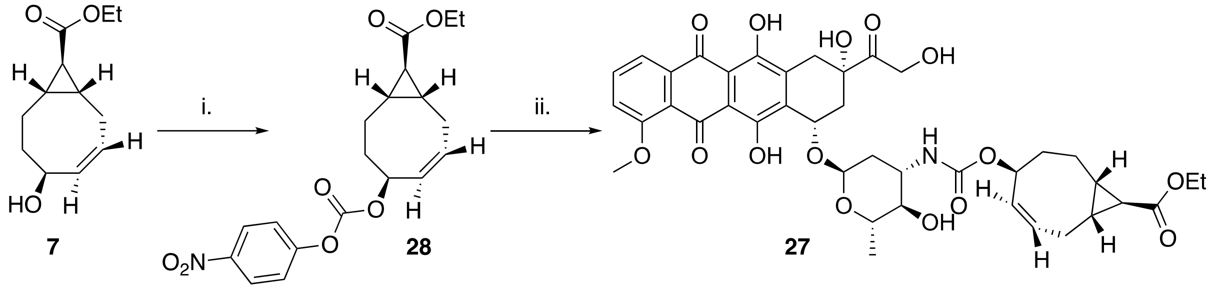

Scheme S5: i. 4-nitrophenyl chloroformate (1.5 equiv), pyridine (2.5 equiv), DCM, 21 °C, 4 h, 70%. ii. doxorubicin hydrochloride. (0.9 equiv), TEA (1.1 eq), DMF, 40 °C, 48 h, 30%.

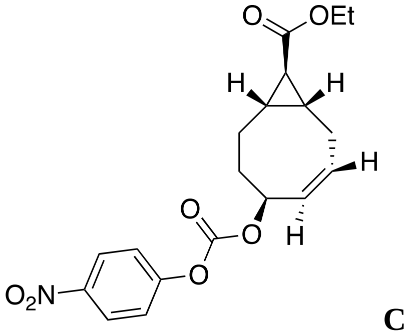

COOEt-*exo*-axial-TCO-PNP (28): Ethyl (1*R*,3*E*,5*S*,8*S*,9*R*,*P*)-5-hydroxybicyclo[6.1.0]non-3-ene-9-carboxylate (**7**, 99 mg, 471 µmol) was dissolved in dry DCM (10 mL) and pyridine (95 µL, 1.18 mmol) was added. A solution of 4-nitrophenyl chloroformate (85 mg, 424 µmol) in dry DCM (3 mL) was added. The reaction mixture was stirred for 4 h before it was quenched with aqueous sat. NH_4Cl (10 mL). The phases were separated and the aqueous phase was extracted with DCM (2 × 15 mL) and the combined organic layers were dried with $MgSO_4$, concentrated *in vacuo* and purified with silica gel column chromatography (0 → 10% EtOAc in pentane) to afford **28** (65 mg, 70%) as an inseparable mixture of the product and starting 4-nitrophenyl chloroformate. **TLC** (EtOAc/pentane, 1:9 v/v): R_F = 0.27. **^{1}H NMR** (400 MHz, $CDCl_3$) δ 8.30–8.24 (m, 2H), 7.41–7.35 (m, 2H), 6.27 (ddd, *J* = 16.9, 10.9, 5.9 Hz, 1H), 5.59 (dd, *J* = 17.0, 3.3 Hz, 1H), 5.18 (q, *J* = 3.0 Hz, 1H), 4.12 (qd, *J* = 7.2, 1.6 Hz, 2H), 2.74 (dt, *J* = 12.7, 6.2 Hz, 1H), 2.56–2.46 (m, 1H), 2.33–2.19 (m, 2H), 2.09–2.00 (m, 1H), 1.88 (t, *J* = 13.8 Hz, 1H), 1.63–1.55 (m, 2H), 1.53–1.47 (m, 1H), 1.27 (t, *J* = 7.1 Hz, 3H). **^{13}C NMR** (101 MHz, $CDCl_3$) δ 174.9, 155.6, 150.1, 146.0, 133.3, 128.2, 125.4, 121.8, 75.9, 60.8, 37.5, 31.4, 29.5, 26.6, 20.7, 14.4. **HRMS** (m/z): $[M + Na]^+$ calcd. for $C_{19}H_{21}N_1O_7Na$: 398.1215, found 398.1228.

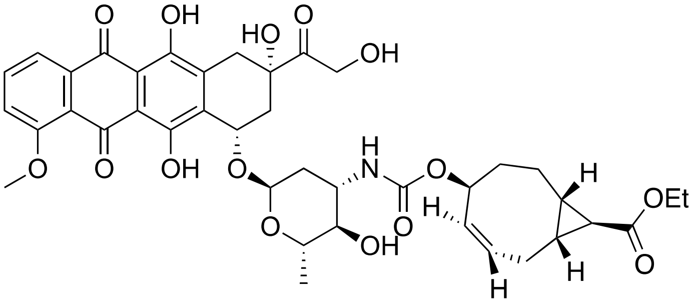

COOEt-*exo*-axial-TCO-Doxorubicine (27): COOEt-R-TCO-PNP (**28**, 6.5 mg, 17 µmol) was dissolved in dry DMF (2 mL) and triethylamine (2.6 µL, 19 µmol) and doxorubicin hydrochloride (9.0 mg, 16 µmol) were added. The reaction mixture was stirred in the dark overnight. LCMS indicated still a significant amount of starting material so the temperature was raised to 40 °C and the reaction was stirred for another night. The reaction mixture was concentrated *in vacuo* and the crude product was purified using reversed-phase preparative HPLC (0 → 100 % MeCN (0.1% formic acid) in MiliQ (0.1% formic acid)) and lyophilized to afford **27** (3.8 mg, 30%). **TLC** (DCM/MeOH, 9:1 v/v): R_F = 0.07. **^{1}H NMR** (500 MHz, $CDCl_3$) δ 14.02 (s, 1H), 13.29 (s, 1H), 8.07 (d, *J* = 7.7 Hz, 1H), 7.81 (td, *J* = 8.1, 2.1 Hz, 1H), 7.44–7.39 (m, 1H), 6.16–5.99 (m, 1H), 5.58–5.48 (m, 2H), 5.37–5.30 (m, 1H), 5.18–5.11 (m, 1H), 5.03–4.97 (m, 1H), 4.61–4.49 (m, 1H), 4.21–4.14 (m, 2H), 4.11 (s, 3H), 4.09–4.03 (m, 1H), 3.92–3.83 (m, 1H), 3.73–3.66 (m, 1H), 3.35–3.25 (m, 1H), 3.11–3.03 (m, 1H), 2.47–2.32 (m, 2H), 2.09–2.02 (m, 2H), 1.93–1.86 (m, 2H), 1.73 (d, 7H), 1.28–1.26 (m, 6H). **^{13}C NMR** (126 MHz, $CDCl_3$) δ 214.0, 187.3, 186.9, 175.1, 161.2, 156.3, 155.8, 154.9, 135.9, 135.7, 133.7, 131.5, 129.9, 129.7, 121.0, 120.0, 118.6, 111.7, 111.6, 100.8, 71.4, 69.7, 67.4, 65.6, 60.6, 56.8, 50.9, 47.0, 46.0, 37.5, 35.8, 34.9, 32.0, 31.0, 29.8, 26.9, 22.8, 21.0, 17.0, 14.2. **HRMS** (m/z): $[M + Na]^+$ calcd. for $C_{40}H_{45}N_1O_{15}Na$: 802.2686, found 802.2710.

3. Stability assays

a. Stability test in PBS

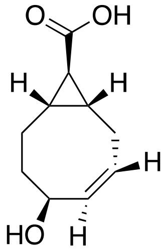

(1*R*,3*E*,5*S*,8*S*,9*R*,*P*)-5-hydroxybicyclo[6.1.0]non-3-ene-9-carboxylic acid (29): LiOH (172 mg, 7.20 mmol) was added to a stirred solution of ethyl (1*R*,3*E*,5*S*,8*S*,9*R*,*P*)-5-hydroxybicyclo[6.1.0]non-3-ene-9-carboxylate (**7**, 500 mg, 2.40 mmol) in THF (8 mL) and water (4 mL). The mixture was stirred at 50 °C in the dark (flask covered with aluminum foil). After completion was seen on TLC, EtOAc (5 mL) and 1 M aqueous HCl (5 mL) were added to the mixture. The organic layer was washed with brine (5 mL) and both aqueous layers were back-extracted with EtOAc (2 × 5 mL). The combined organic layers were dried with $MgSO_4$ and concentrated *in vacuo* to afford **29** (376 mg, 87%). **TLC** (MeOH/DCM, 1:9 v/v): R_F = 0.23. **^{1}H NMR** (400 MHz, D_2O) δ 6.18 (ddd, *J* = 16.9, 10.8, 5.8 Hz, 1H), 5.63 (dd, *J* = 17.1, 3.4 Hz, 1H), 4.35 (q, *J* = 3.0 Hz, 1H), 2.66 (dt, *J* = 12.8, 6.4 Hz, 1H), 2.43 (t, *J* = 11.8 Hz, 1H), 2.14 (dd, *J* = 15.3, 11.4 Hz, 1H), 1.90–1.74 (m, 3H), 1.44 (t, *J* = 5.9 Hz, 1H), 1.38–1.30 (m, 1H), 1.28–1.21 (m, 1H). **^{13}C NMR** (101 MHz, D_2O) δ 186.8, 134.8, 133.1, 70.4, 41.1, 32.1, 31.0, 27.6, 25.4, 21.3.

<u>Procedure</u>

An NMR tube was charged with a solution of **29** (41 mg) in deuterated PBS buffer (500 µL). The NMR sample was placed in a water bath at 37 °C while being covered from light by aluminum foil. The sample showed no changes in the ^{1}H NMR spectrum after 7 days (see Figure S1).

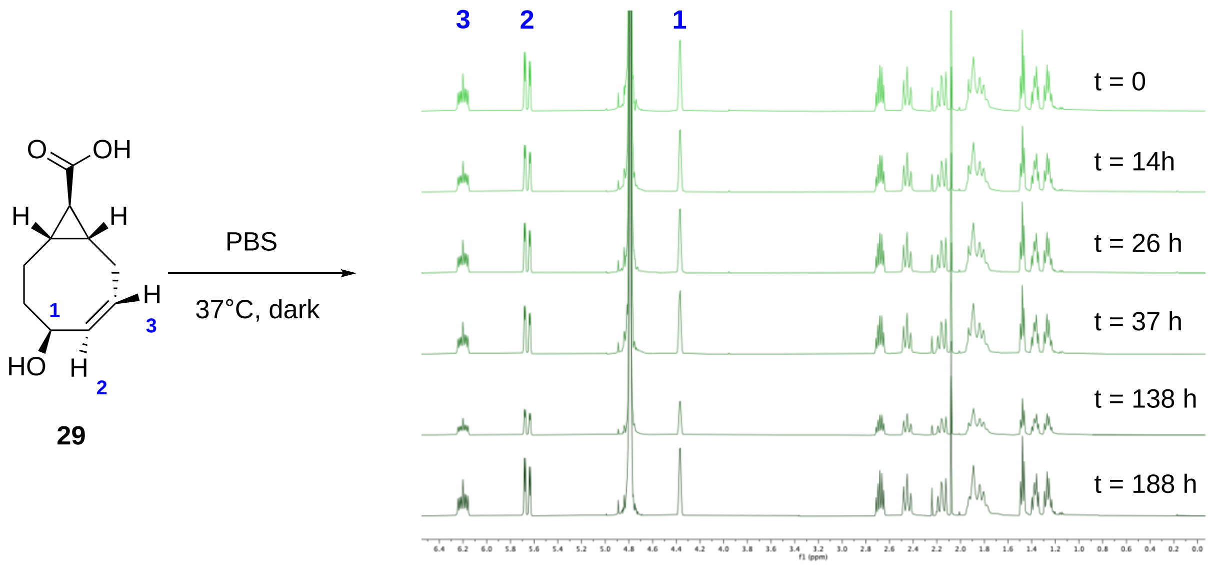

Figure S1. ^{1}H NMR stability assay of the difunctionalized TCO **29** in deuterated PBS buffer at 37 °C.

b. Stability test in mouse serum

Procedure

Stock solution (in DMSO)

- **Stock TCO: 7** (9.9 mg, 500 mmolar)

10 µL of **Stock TCO** was added to 1 mL of mouse serum (Invitrogen):PBS (pH = 7.2) 1:1. The mixture was incubated at 37 °C under mixing (300 rpm). At certain time points after the addition 200 µL was aliquoted and this was extracted with EtOAc (2 × 200 µL), the combined organic layers were concentrated *in vacuo*, dissolved in $CDCl_3$ (500 µL) and a ^{1}H NMR spectrum was recorded (see Figure S2).

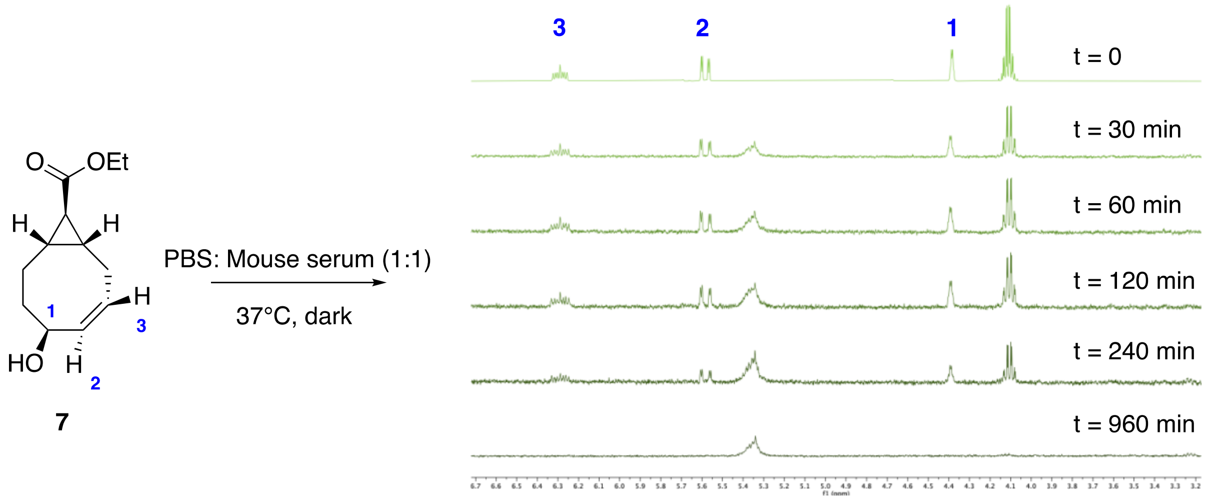

Figure S2. ^{1}H NMR stability assay of the difunctionalized TCO **7** in PBS (pH = 7.2):mouse serum at 37 °C.

4. Kinetics study of the click and release reaction

a. Click reaction kinetics

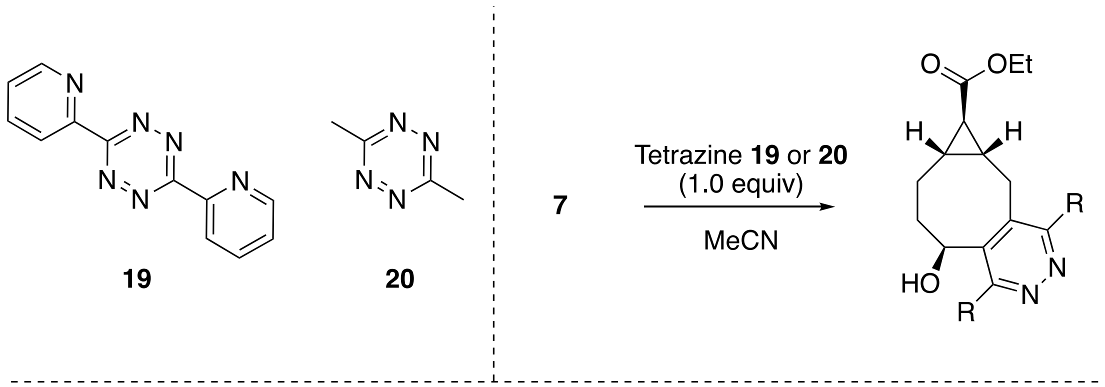

Scheme S6. Second-order rate constants for reaction of TCO 7 with tetrazines **19** and **20**. Values were determined using UV/Vis spectroscopy at 540 nm (specific for tetrazine) in acetonitrile at room temperature.

Procedure

The reaction rate of the TCO tetrazine click reaction was measured under similar conditions as described in Versteegen et al.[3] The second-order reaction constant of the reaction between **7** and 3,6-di-2-pyridyl-1,2,4,5-tetrazine (**19**) or 3,6-dimethyl-1,2,4,5-tetrazine (**20**) was determined in MeCN at 20 °C by UV/Vis-spectroscopy at 540 nm (specific for tetrazine moiety). A cuvette was filled with MeCN (3.0 mL) and equilibrated at 20 °C. A stock solution of tetrazine **19** or **20** in DMSO (50 or 100 μL) was added to the cuvette. Hereafter, A stock solution of TCO **7** (50 or 100 μL) was added and briefly mixed by pipetting (50 μL for 97 μM and 100 μL for 188 μM final concentration). The absorption at 540 nm was measured for 5 minutes (for tetrazine **19**) or 30 minutes (for tetrazine **20**). From this absorption at 540 nm, the concentration of tetrazine was calculated using a molar absorption coefficient of $\varepsilon = 430\ M^{-1}cm^{-1}$ and $l = 1.0$ cm. The second-order rate constant k_2 was obtained from the slope of a plot of $(1/c - 1/c_0)$ versus time plotted in GraphPad Prism (version 9.0). All experiments were performed in triplicate.

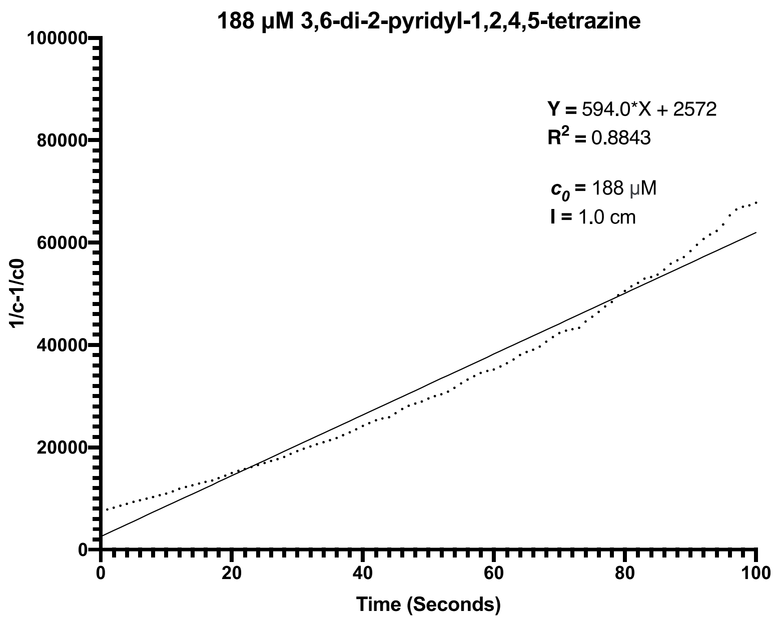

Figure S3. Kinetic plot of the reaction of TCO **7** with tetrazine **19** at 20 °C at 188 μM. K_2 = 594.0 $M^{-1}S^{-1}$ ± 12.38.

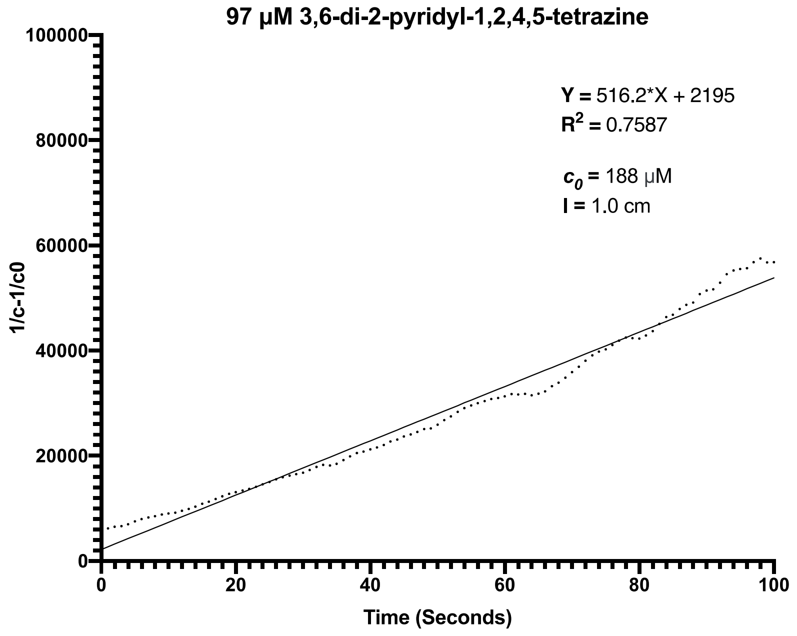

Figure S4. Kinetic plot of the reaction of TCO **7** with tetrazine **19** at 20 °C at 97 μM. K_2 = 516.2 $M^{-1}S^{-1}$ ± 16.78.

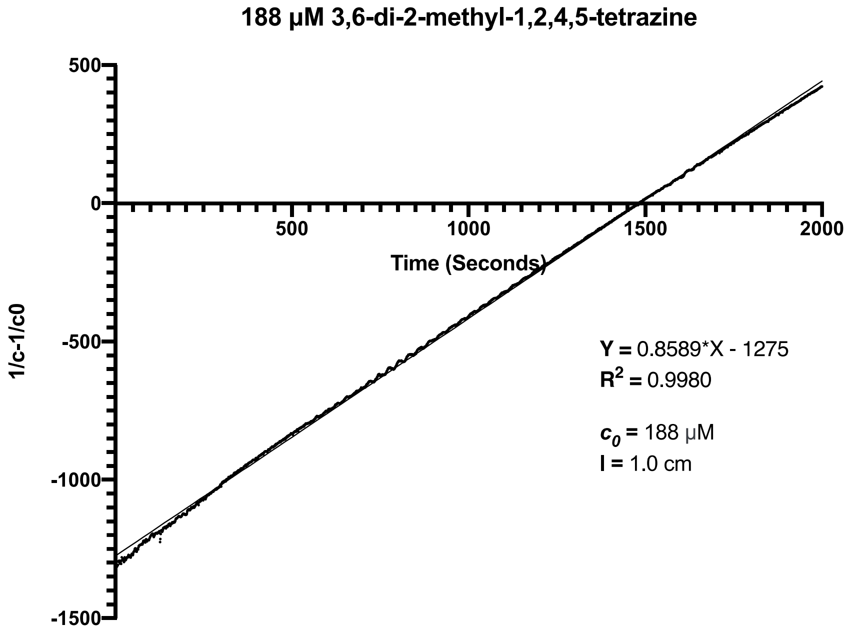

Figure S5. Kinetic plot of the reaction of TCO **7** with tetrazine **20** at 20 °C at 188 μM. K_2 = 0.8589 $M^{-1}S^{-1}$ ± 0.001.

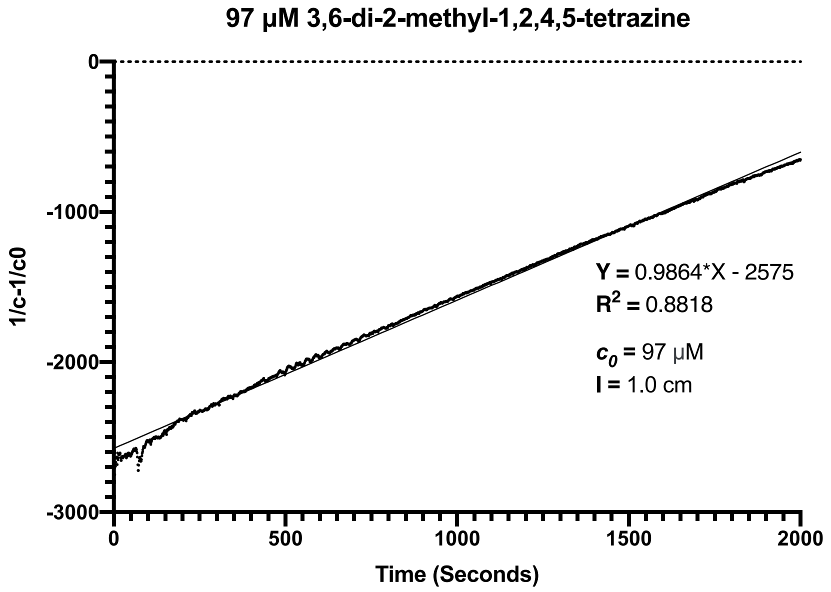

Figure S6. Kinetic plot of the reaction of TCO **7** with tetrazine **20** at 20 °C at 97 μM. K_2 = 0.9864 $M^{-1}S^{-1}$ ± 0.004.

b. PFP carbonate release study with ^{19}F NMR

$$\textbf{15, 16, 17, 18} \xrightarrow[\text{20°, CDCl}_3]{\text{DPTZ}}$$

Scheme S7. ^{19}F NMR click reaction kinetics study of the axial-TCOs **15** and **17** and equatorial-TCOs **16** and **18** by reacting them with DPTZ (**19**, 3,6-di-2-pyridyl-1,2,4,5-tetrazine) in $CDCl_3$.

Procedure

The ^{19}F NMR (377 MHz) spectrum of an NMR-tube filled with a solution of **15**, **16**, **17**, or **18** (5 mg, 11.9 μmol) in $CDCl_3$ (500 μL) was measured. Next, a solution of 3,6-di(pyridin-2-yl)-1,2,4,5-tetrazine (**19**, 3.1 mg, 13 μmol) in $CDCl_3$ (100 μL) was added to the NMR tube. The tube was vigorously shaken and placed back in the NMR apparatus. The sample was measured within two minutes after the addition of the second compound. Every 48 seconds a measurement (number of scans: 4, relaxation delay: 5.8 sec, range: −147.0 to −178.5 ppm) was taken of the sample. Almost complete release of PFP was seen after 20 minutes. With 20 mM TCO **15** and 3,6-di(pyridin-2-yl)-1,2,4,5-tetrazine (**19**) at 20 °C, complete release of PFP was observed after 20 minutes upon the click reaction (Figure S7). When using **16** instead, release was found to be several factors slower. Further investigation of the **15** click and release reaction at lower temperatures showed that the release was instantaneous upon tautomerization from the click product (Figure S8). ^{19}F NMR kinetic study of the click reaction of 40 mM with tetrazine **19** was performed at controlled temperatures. At −20 °C the click conjugate and tetrazine **19** could be observed and did not show any formation of PFP. Only after elevating the temperature to +10 °C, the PFP carbonate was released from the conjugated product. Release of payload from compounds was not only found to be fast, but also complete. Versteegen *et al.* tested various payloads on the allylic position of TCO compounds known from the prior art and observed only a maximum of 60% release under conditions that were comparable to those used in our ^{19}F NMR studies.[4] The fastest payload release observed with their compounds afforded less than 20% release over 250 minutes. Compounds in this paper showed near complete release within 20 minutes.

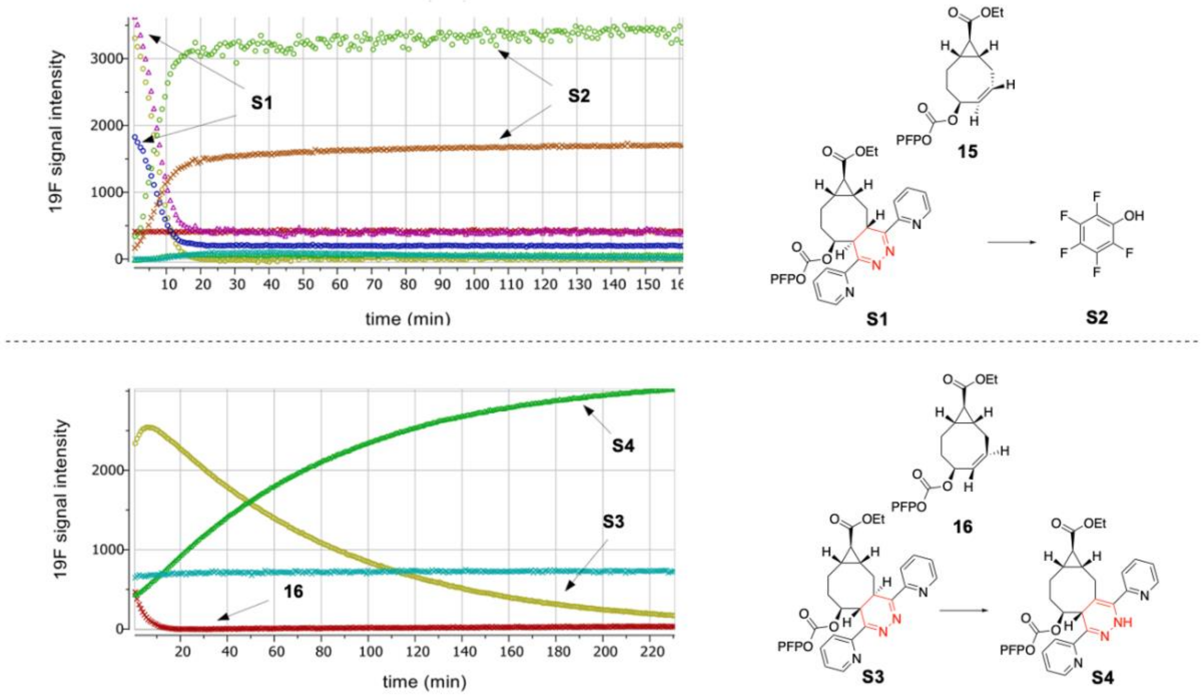

Figure S7. ^{19}F NMR kinetic study of the click reaction of 200 mM *exo* axial (top) and *exo* equatorial (bottom) TCOs **15** and **16** with dipyridyl tetrazine (**19**) at 20 °C. Axial TCO **15** showed complete release of pentafluorophenol (**S2**) upon the click reaction with tetrazine **19** after 20 minutes. Only the click conjugate **S1** was observed without the intermediate tautomerization product. Equatorial TCO **16** did not show release of pentafluorophenol (**S2**). Only click conjugate **S3** and the tautomerization product **S4** were observed.

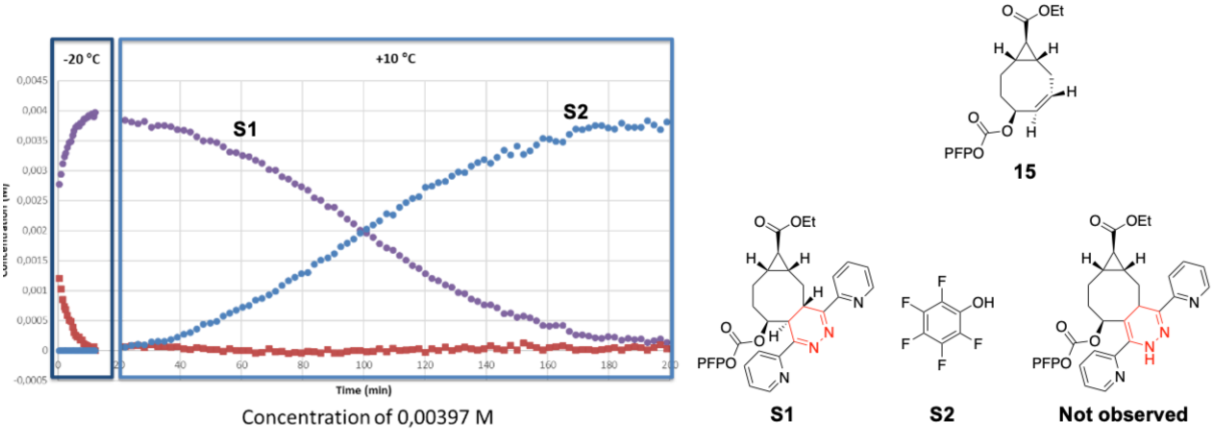

Figure S8. ^{19}F NMR kinetic study of the click reaction of 40 mM axial TCO **15** with tetrazine **19** at −20 °C and +10 °C. At −20 °C the click conjugate (**S1**) could be observed and did not show any formation of pentafluorophenol (**S2**). Only after elevating the temperature to +10 °C, the pentafluorophenol (**S2**) was released from the conjugated product.

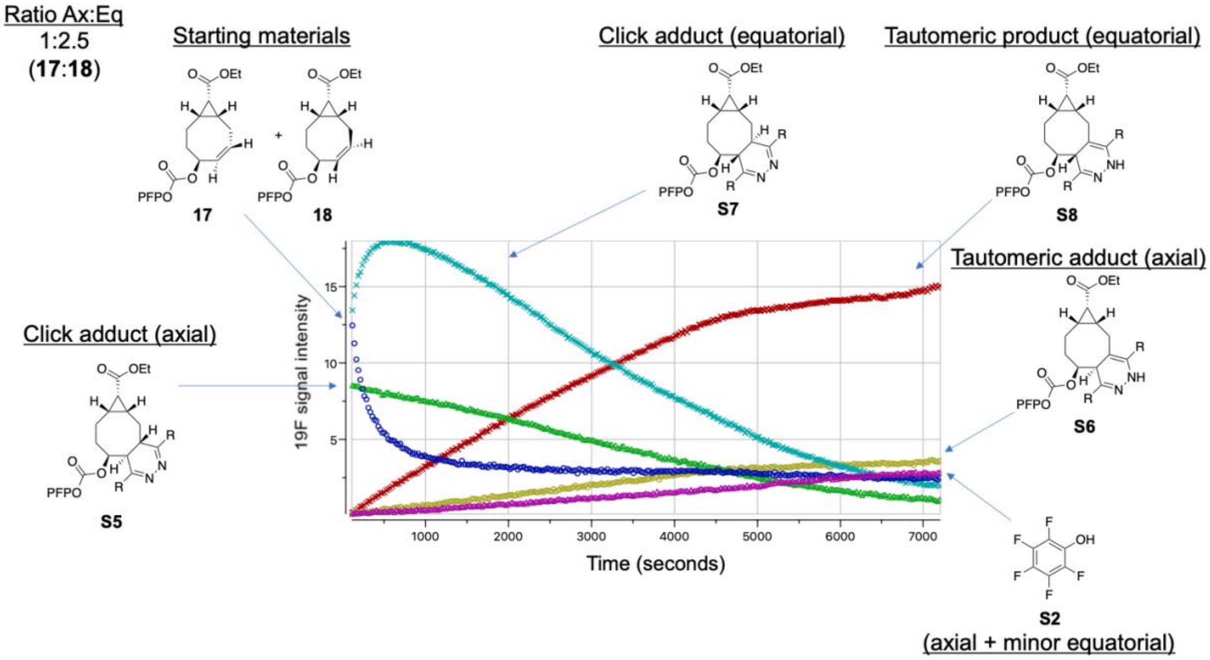

Figure S9. ^{19}F NMR kinetic study of the click reaction of 200 mM *endo* axial and equatorial (mix, ratio 1:2.5 respectively) TCO **17** and **18** with tetrazine **19** at 20 °C. The axial TCO **17** showed release of pentafluorophenol (**S2**) upon the click reaction with dipyridyl tetrazine (**19**) but also the pre-tautomeric adduct (**S6**). Equatorial TCO **18** did not show release of pentafluorophenol (**S2**). Only the click conjugate (**S7**) and the tautomerization product (**S8**) were observed.

c. AMC carbamate release study with fluorescence spectroscopy

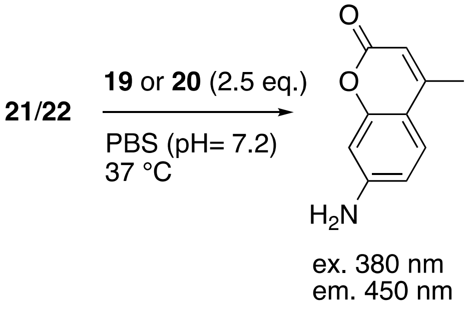

Scheme S8. Study conditions, either **21** or **22** was incubated with tetrazine **19** or **20** and the fluorescence (excitation: 380 nm, emission: 240 nm) was measured at certain time points after the addition of the tetrazine.

Stock solutions (all in DMSO)

- **Stock A (R): 21** (10 mmolar)
- **Stock A (NR): 22** (10 mmolar)
- **Stock B (BP): 19** (20 mmolar)
- **Stock B (DM): 20** (20 mmolar)
- **Stock PC:** 7-amino-4-methylcoumarin (10 mmolar)

Procedure[5]

2.5 µL of the tetrazine **stock** (**DM** or **BP**) was mixed with 395.5 µL of PBS (pH = 7.2). 2 µL of the TCO stock (**A (R)** or **A (NR)**) was added to this. The reaction mixtures were incubated at 37 °C under mixing (300 rpm). Subsequently, the fluorescence (**F1**) was measured (ex: 380 nm, em. 450 nm) at certain time points after the addition of the TCO by aliquoting 30 µL in a 384 wells plate (Greiner, black non-binding flat bottom) and directly measuring with a Tecan Spark plate-reader. As control experiment the maximum amount of released AMC was measured (**F2**, **Stock PC** without tetrazine or TCO). Negative control experiments revealed no significant fluorescent signal for either exclusively the tetrazine or the TCO's. The release efficiency (%) was calculated: **F1**/**F2** × 100%, as similar to reported previously in the literature.[5] All experiments were executed in triplicate and the data was processed using GraphPad Prism (version 9.0). The $K_{elim.}$ values were determined by first-order exponential one phase decay approximations.

d. Glycine/sarcosine release study with ^{1}H NMR

Procedure

The reaction of TCOs (**23** - **26**) with 3,6-dimethyl-1,2,4,5-tetrazine (**20**, 5 mM, 1:1 ratio) in deuterated PBS/MeOD (1:1 v/v) were followed via ^{1}H NMR. 3-(trimethylsilyl)-1-propanesulfonic acid-d_6 sodium salt (0.2 mM) was used as internal standard to determine the ratio of conversion. Upon sample preparation, the sample was measured at the indicated time points. In between these measurements, the sample was removed from the machine and present bubbles were removed by tapping. The signal intensity was normalized using the internal standard. The protons of the -C**H**$_3$ groups of the tetrazine were followed for both the click- (■) and release product (●).

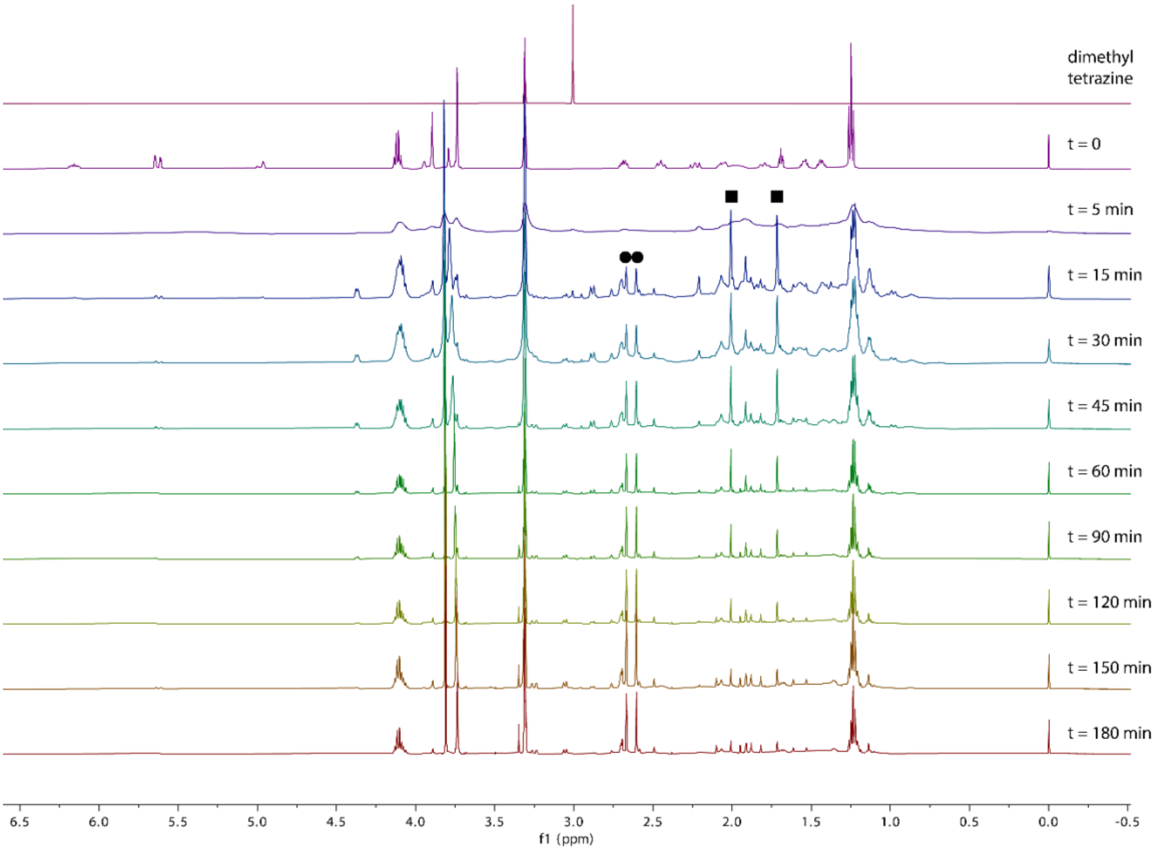

Figure S10. ^{1}H NMR study of 5 mM **23** triggered by 3,6-dimethyl-1,2,4,5-tetrazine (**20**) in deuterated PBS/MeOD (1:1 v/v) at 26 °C. Immediately after combination of the compounds, intense bubble formation prevented proper shimming of the instrument. After 15 minutes both the click product (■) and release product (●) were observed. The signal intensity of the click product was at a maximum after 15 minutes, thereafter it decreased and nearly disappeared. The signal intensity of the release product kept increasing for 3 h.

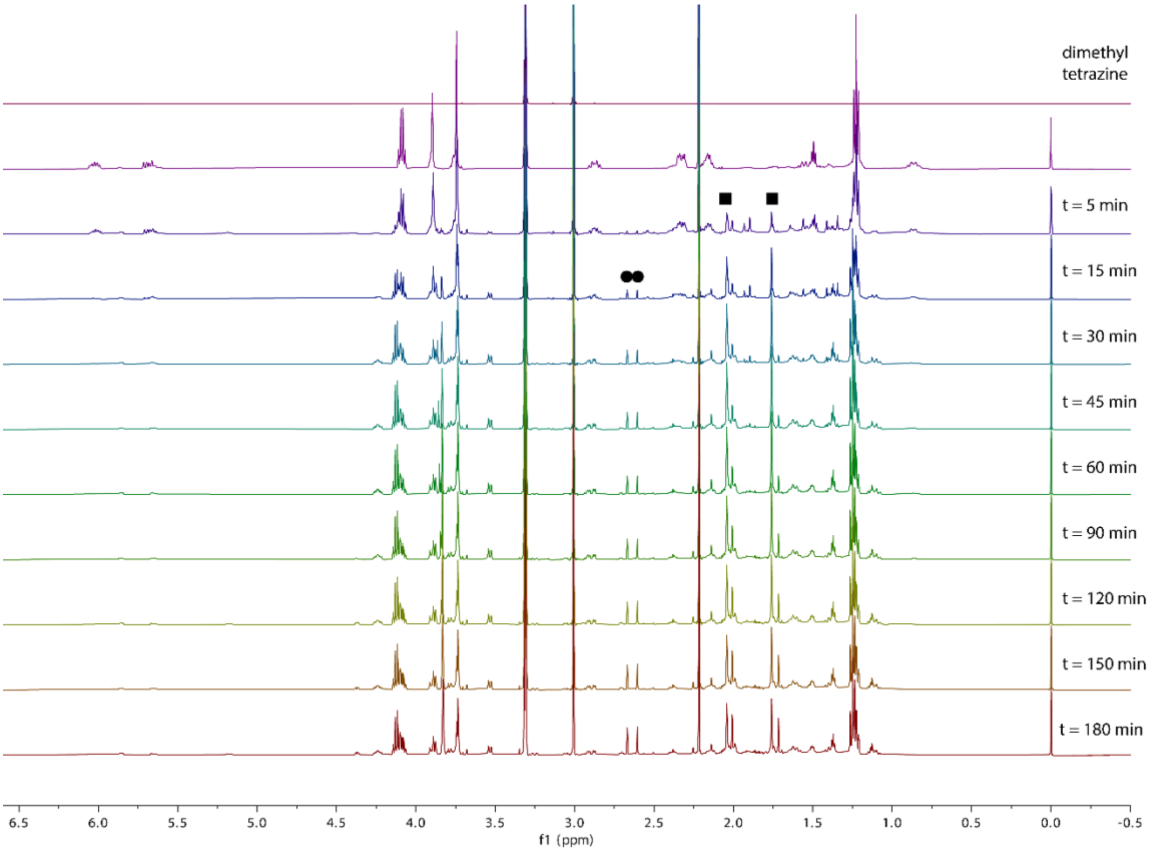

Figure S11. ^{1}H NMR study of 5 mM **24**, triggered by 3,6-dimethyl-1,2,4,5-tetrazine (**20**) in deuterated PBS/MeOD (1:1 v/v) at 26 °C. After 5 minutes the click product (■) and traces of the release product (●) were observed. The signal intensity of the click product reached a plateau after 45 minutes. The signal intensity of the release product was much lower than that of the click-product, but also plateaued after 45 minutes.

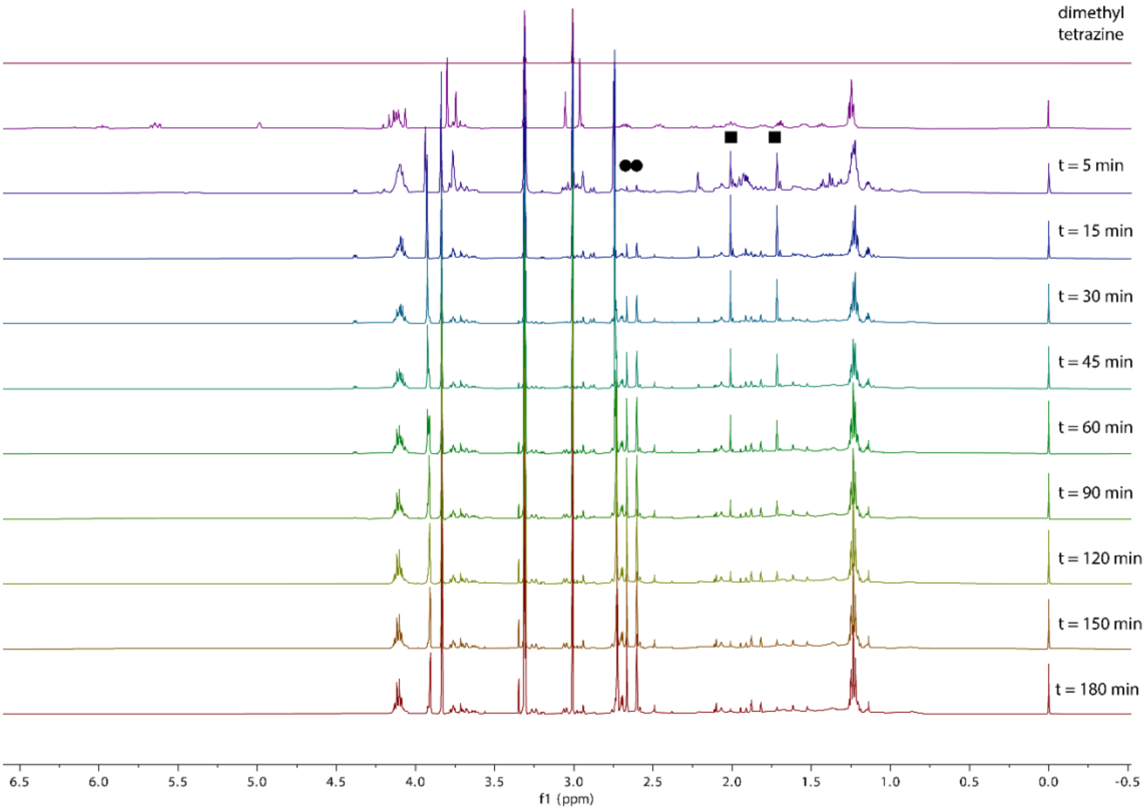

Figure S12. ^{1}H NMR study of 5 mM **25**, triggered by 3,6-dimethyl-1,2,4,5-tetrazine (**20**) in deuterated PBS/MeOD (1:1 v/v) at 26 °C. After 5 minutes both the click product (■) and release product (●) were observed. The signal intensity of the click-product increased in the first 15 minutes, after which it decreased and nearly disappeared. The signal intensity of the release product kept increasing for 3 h.

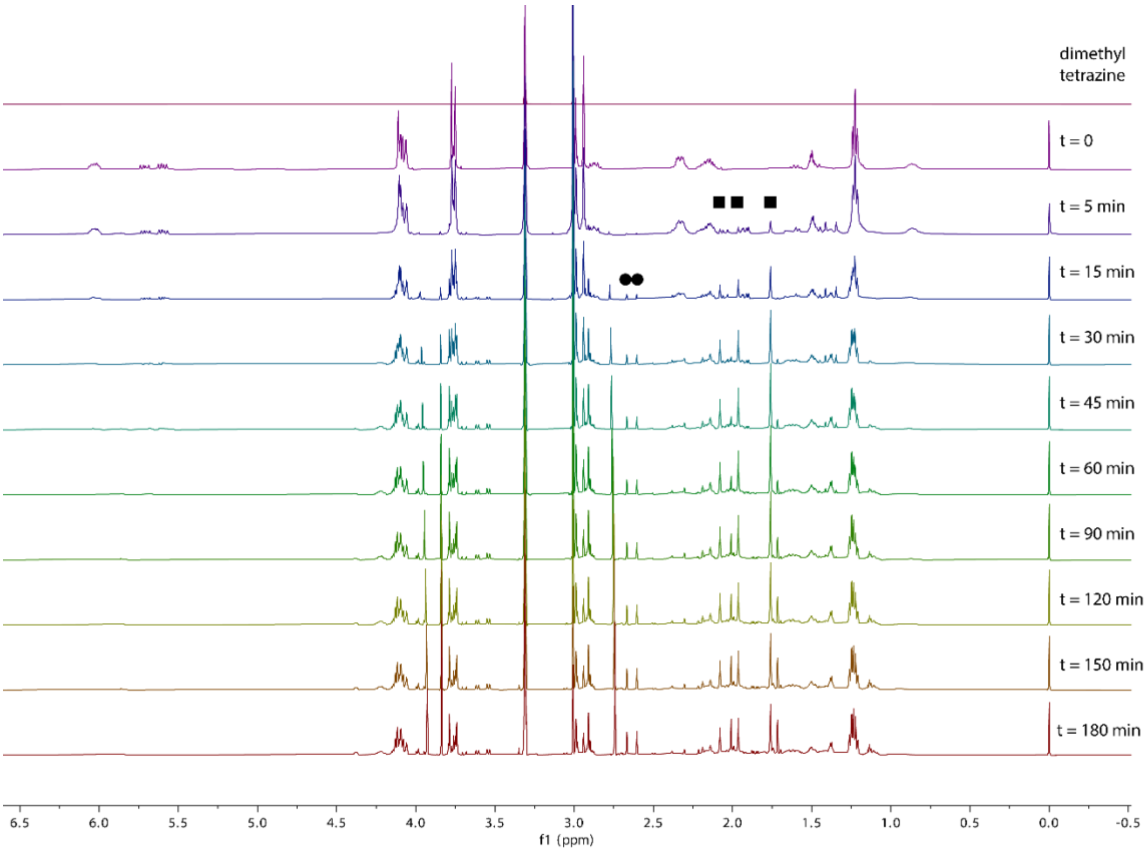

Figure S13. ^{1}H NMR study of 5 mM **26**, triggered by 3,6-dimethyl-1,2,4,5-tetrazine (**20**) in deuterated PBS/MeOD (1:1 v/v) at 26 °C. After 5 minutes both the click-product (■) and traces of the release product (●) were observed. Three signals were observed for the click-product, these can be explained by the rotameric properties of the TCO-Sarcosine. The signal intensity of the click product plateaued after 45 minutes. The signal intensity of the release product was lower than that of the click product, but similarly plateaued after 60 minutes.

e. Cell viability assay

Cell culture

HeLa cells were maintained in DMEM medium, supplemented with 10% fetal bovine serum, 100 units/mL penicillin and 100 µg/mL streptomycin (all purchased at Life Technologies). All cells were incubated at 37 °C and 5% CO_2 passaged every 3−4 days.

EC_{50} assay

The toxicity of several compounds was assessed with a cell proliferation test using Cell Counting Kit – 8 (CCK-8, Sigma-Aldrich). Cells were plated on a 96-wells plate at a density of 1,000 cells per well. After 1 day of attachment, indicated concentrations of doxorubicin (DOX), TCO-DOX (**27**) and tetrazine (**20**) were added to the first columns of wells and continuously diluted. After the cells were incubated for 72 h, the medium was removed and 100 μL of growth medium containing 10% CCK-8 was added. Upon 1 h of incubation, the absorbance was measured at 450 nm using a micro plate reader (Spark M10, Tecan). Background absorbance of the growth medium containing 10% CCK-8 was subtracted from the measured values. The cell viability was normalized to the absorbance of the cells that were incubated with only supplemented growth medium. All conditions were measured six-fold, mean values with SD are shown. EC_{50} values were calculated using the nonlinear regression (curve fit) log(agonist) vs. response -- variable slope (four parameters) equation in GraphPad Prism (version 9.0).

f. Doxorubicin release study

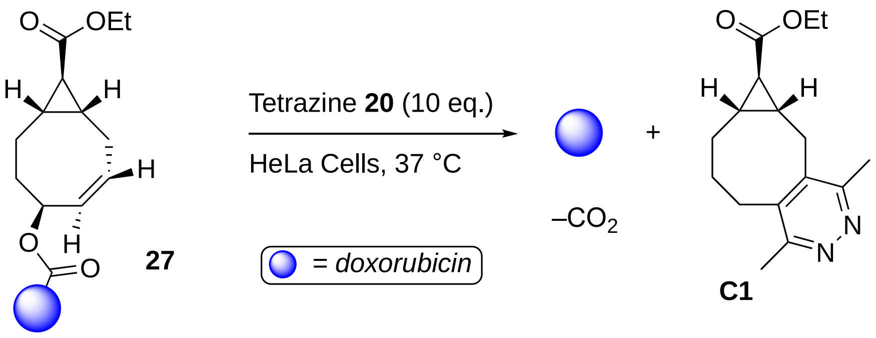

Scheme S9. Doxorubicin release study of **27** induced by the click reaction with tetrazine **20**.

Stock solutions (all in DMSO)

- **Stock TCO-Dox: 27** (10 mmolar)
- **Stock Ttz: 20** (20 mmolar)

Procedure

100 µL of the TCO-Dox stock was added to 900 µL of PBS (pH = 7.2) and HPLC analysis was done which only indicated starting material (t = 13.08 min). Hereafter the reaction was incubated at 37 °C under mixing (300 rpm) and 500 µL of the Ttz stock was added. At certain time points after the addition of the Ttz the reaction mixture was analyzed by aliquoting 200 µL and analysis by HPLC and LCMS. Directly after the addition of the tetrazine (**20**, t = 6.11 min) almost instant release of doxorubicin (t = 7.57 min) and formation of the elimination product (**C1**, t = 7.19 min) was observed (Figure S14).

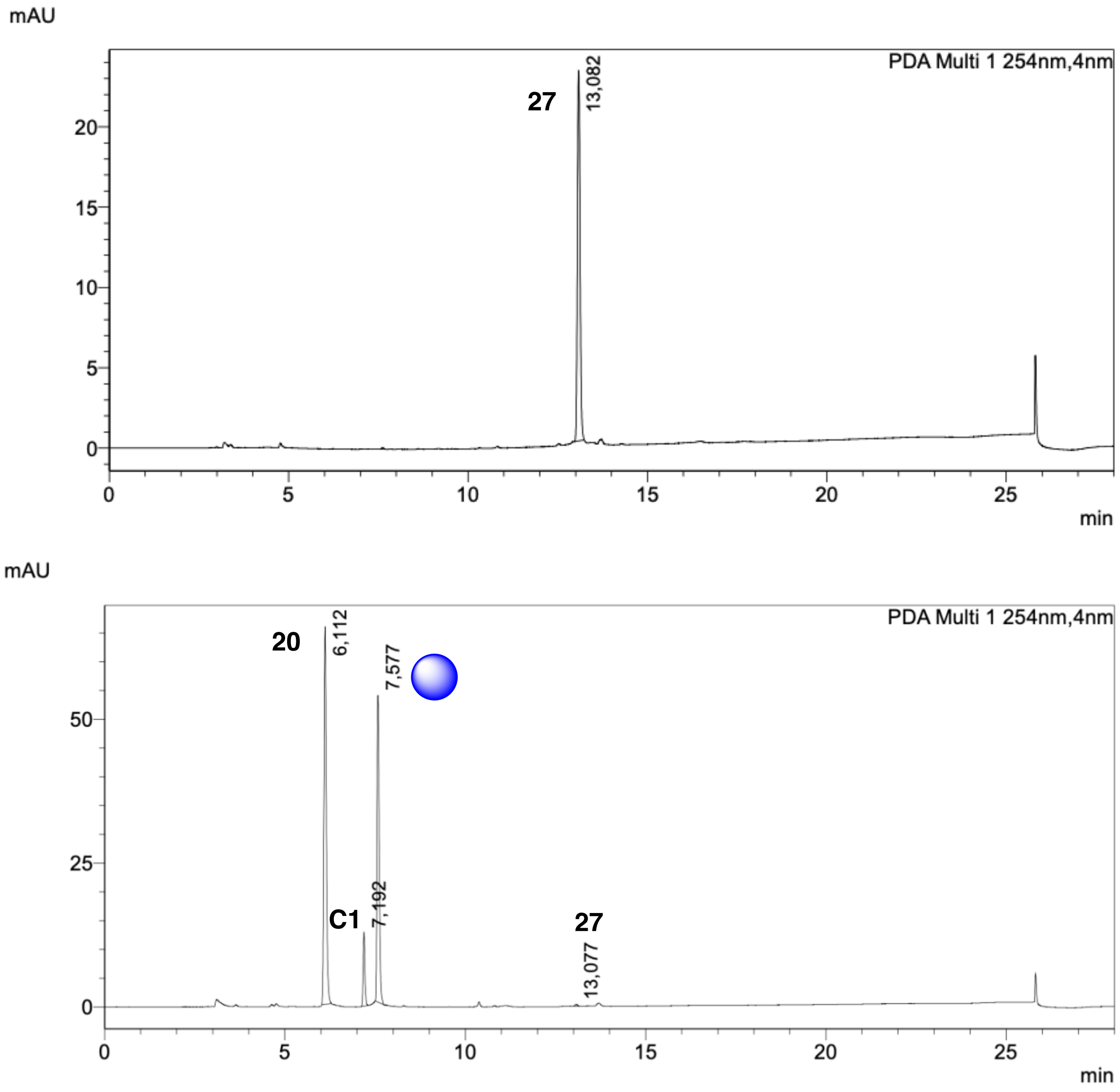

Figure S14. Doxorubicin release study of **27** induced by the click reaction with tetrazine **20**. The HPLC trace (254 nm) was measured before the addition of **20** (top spectrum) and directly after the addition (bottom spectrum). Addition of **20** revealed almost instant release of doxorubicin (blue dot) and formation of the elimination product **C1**.

5. Rotamer determination

To prove that compounds **25** and **26** are both rotamers, they were subjected to ^{1}H NMR measurements at elevated temperatures to observe coalescence. Additionally, EXSY measurements with mix times of 100 ms and 1 s were performed to confirm that increased conformational rotation led to coalescence.

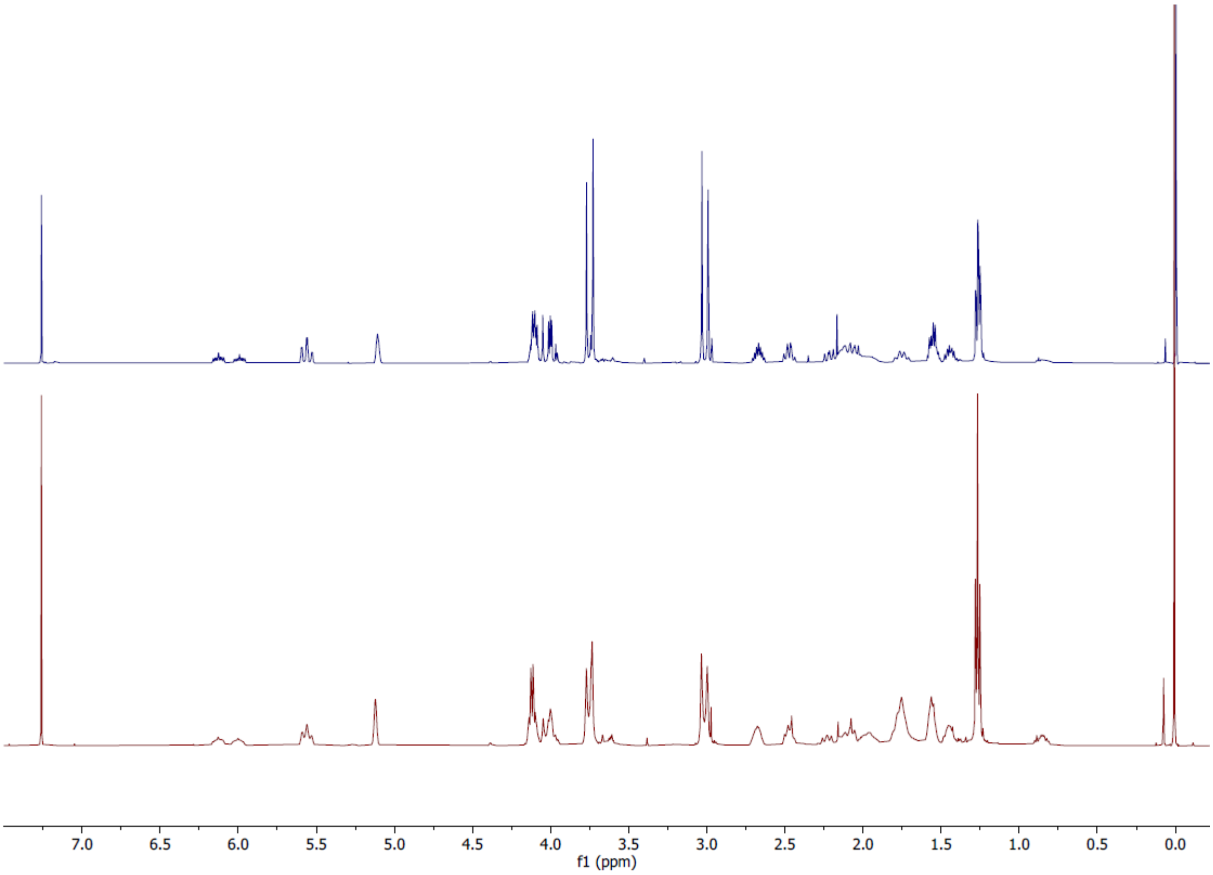

Figure S15. Heat induced coalescence of **25** (axial sarcosine) The ^{1}H spectra were measured at 26 °C (top, navy) and 50 °C (bottom, maroon).

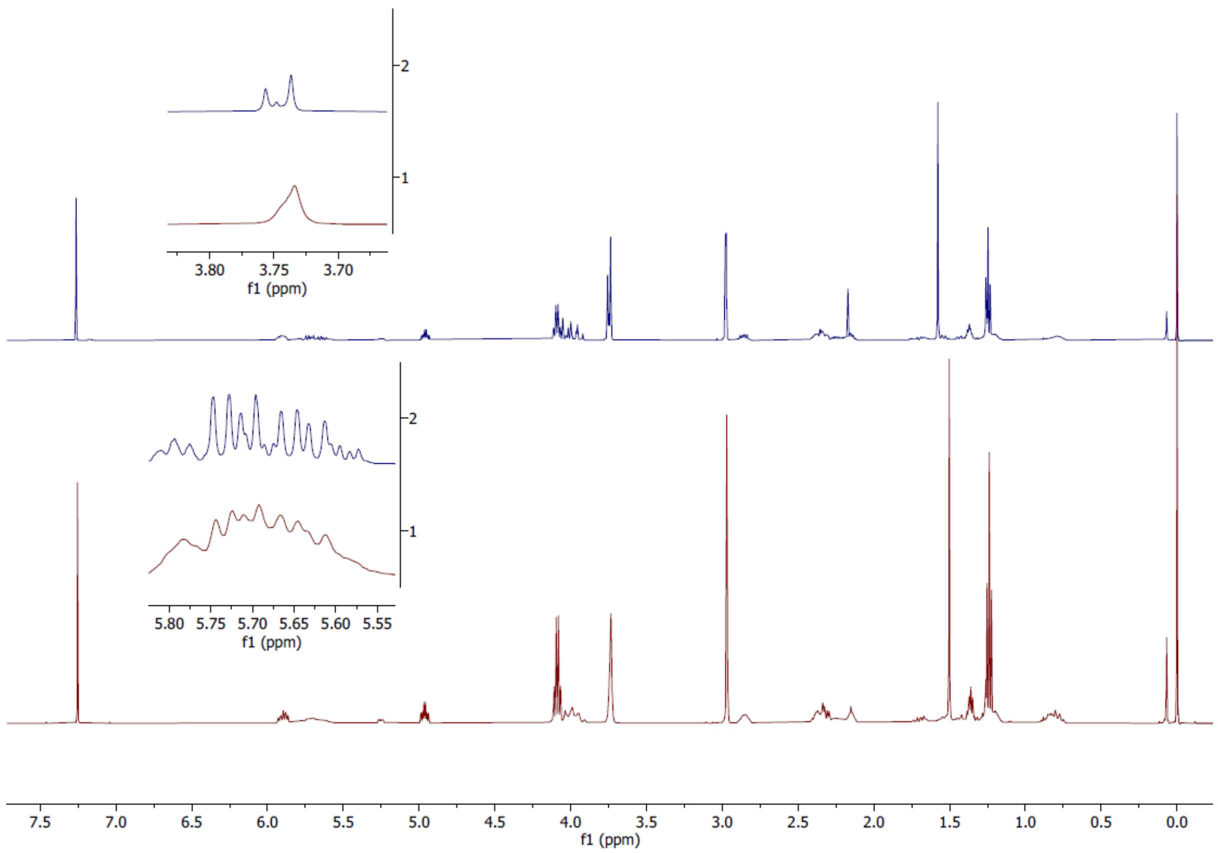

Figure S16. Heat induced coalescence of **26** (equatorial sarcosine) The ^{1}H spectra were measured at 26 °C (top, navy) and 50 °C (bottom, maroon).

6. NMR spectra

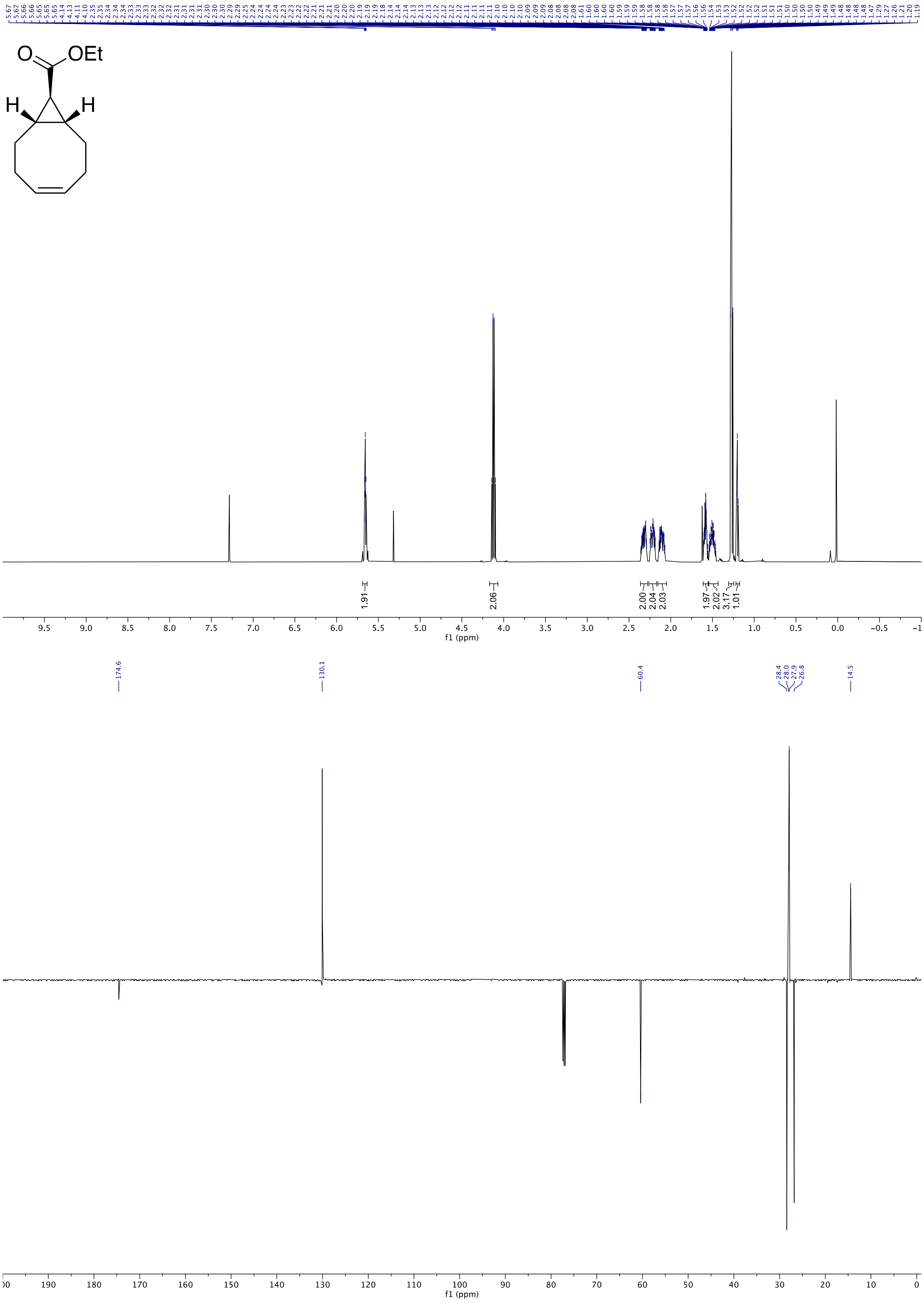

Figure S17. ^{1}H and ^{13}C NMR spectrum of compound **2** (*exo*).

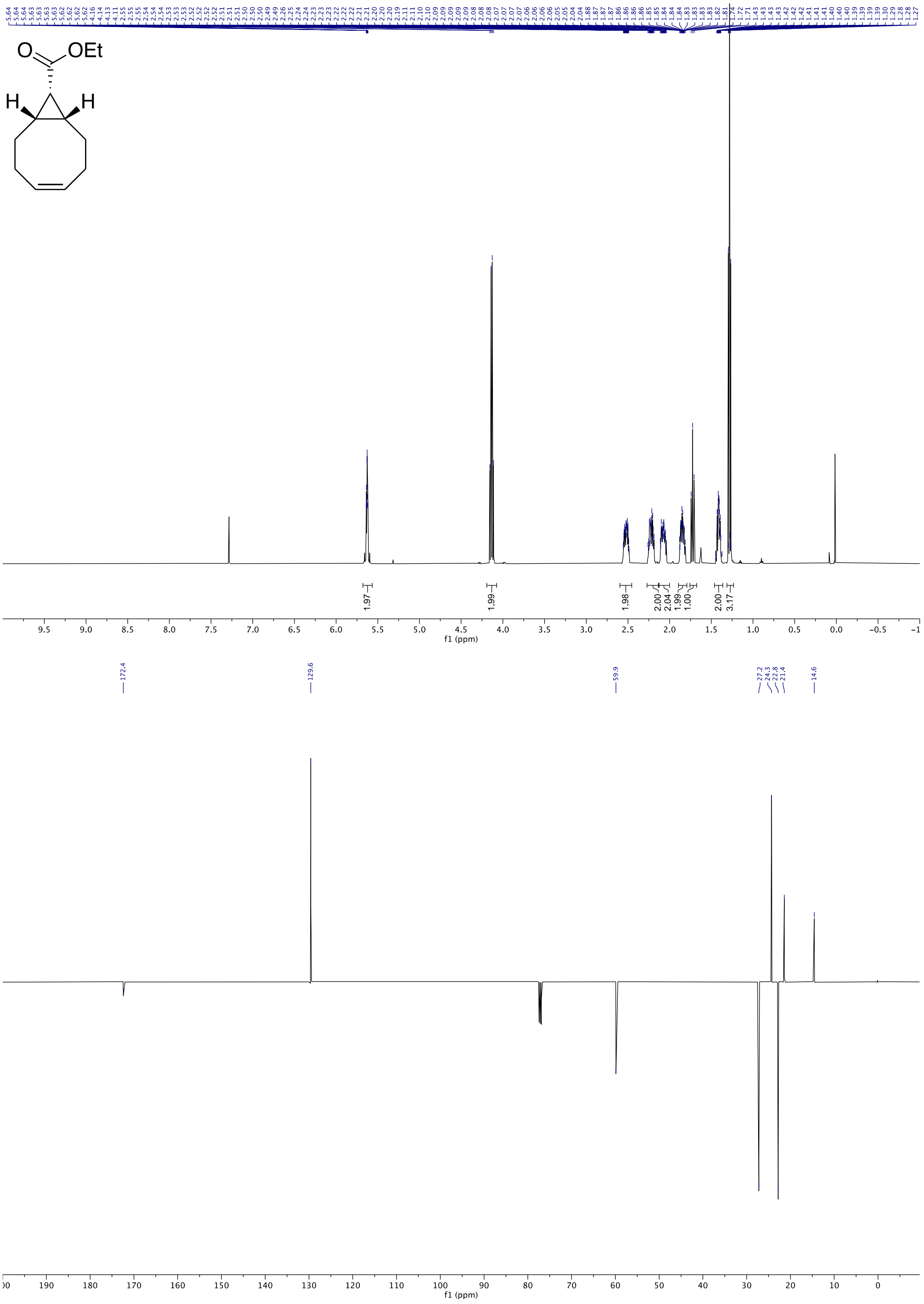

Figure S18. ^{1}H and ^{13}C NMR spectrum of compound **2** (*endo*).

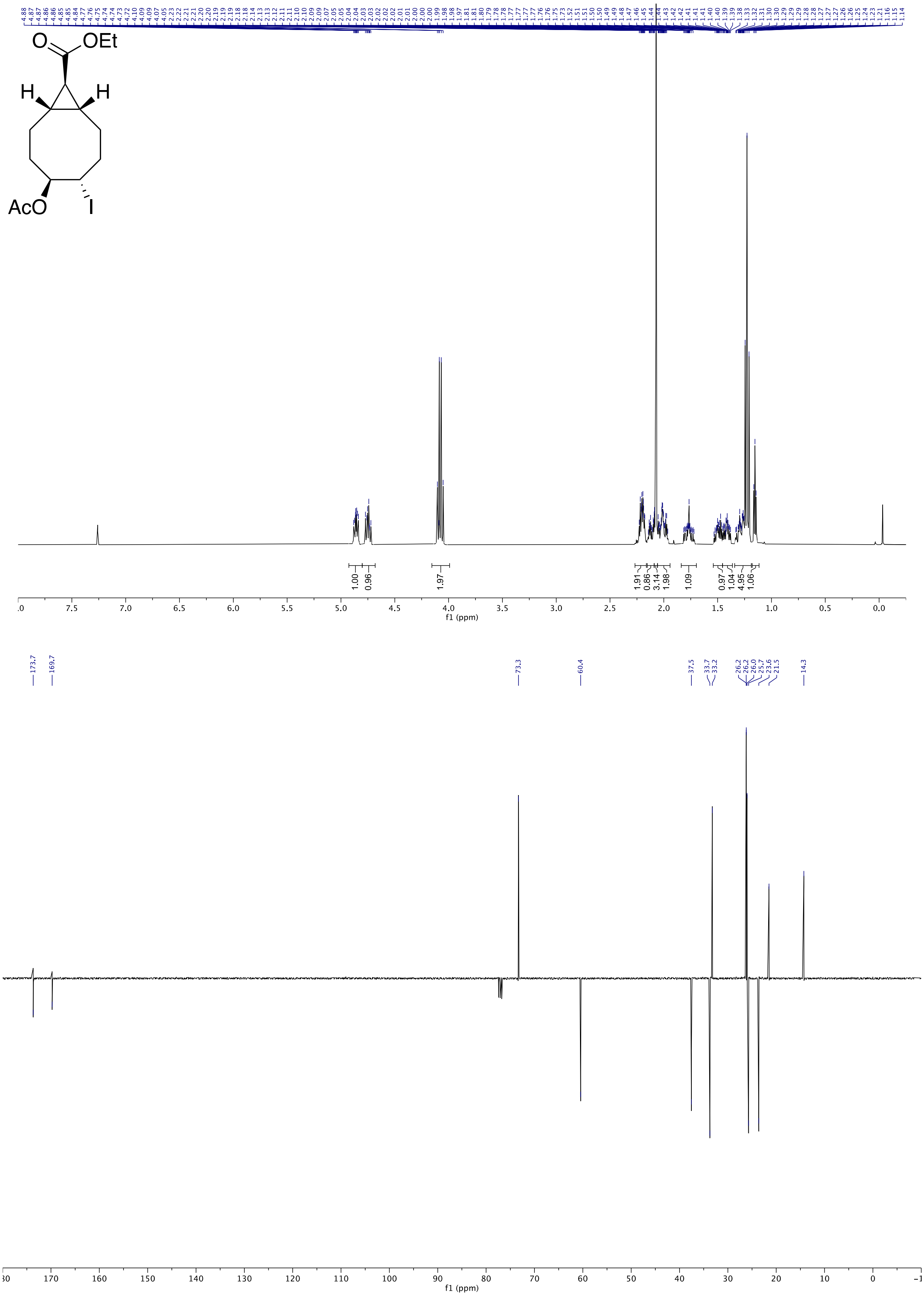

Figure S19. ^{1}H and ^{13}C NMR spectrum of compound **3**.

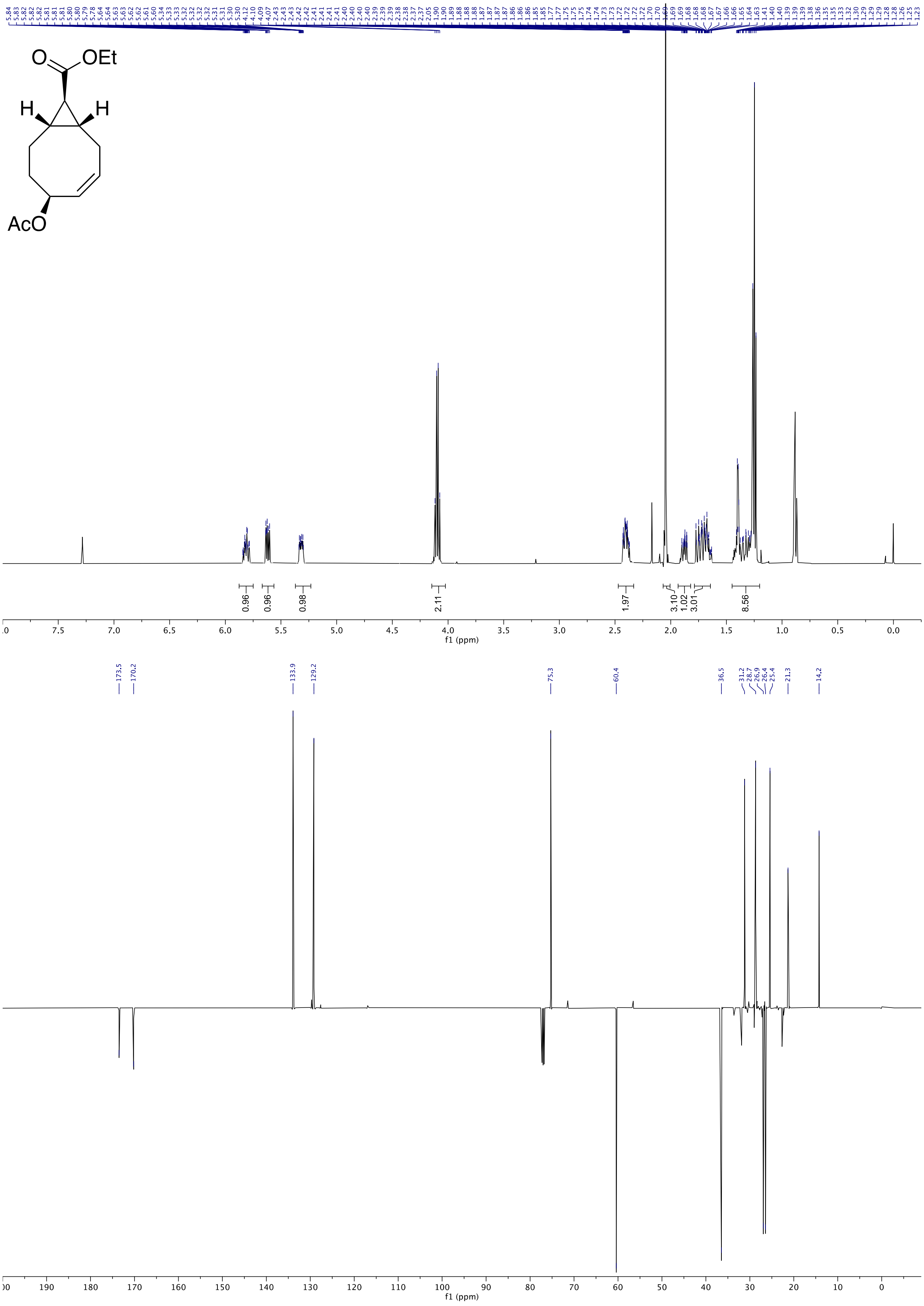

Figure S20. ^{1}H and ^{13}C NMR spectrum of compound **4**.

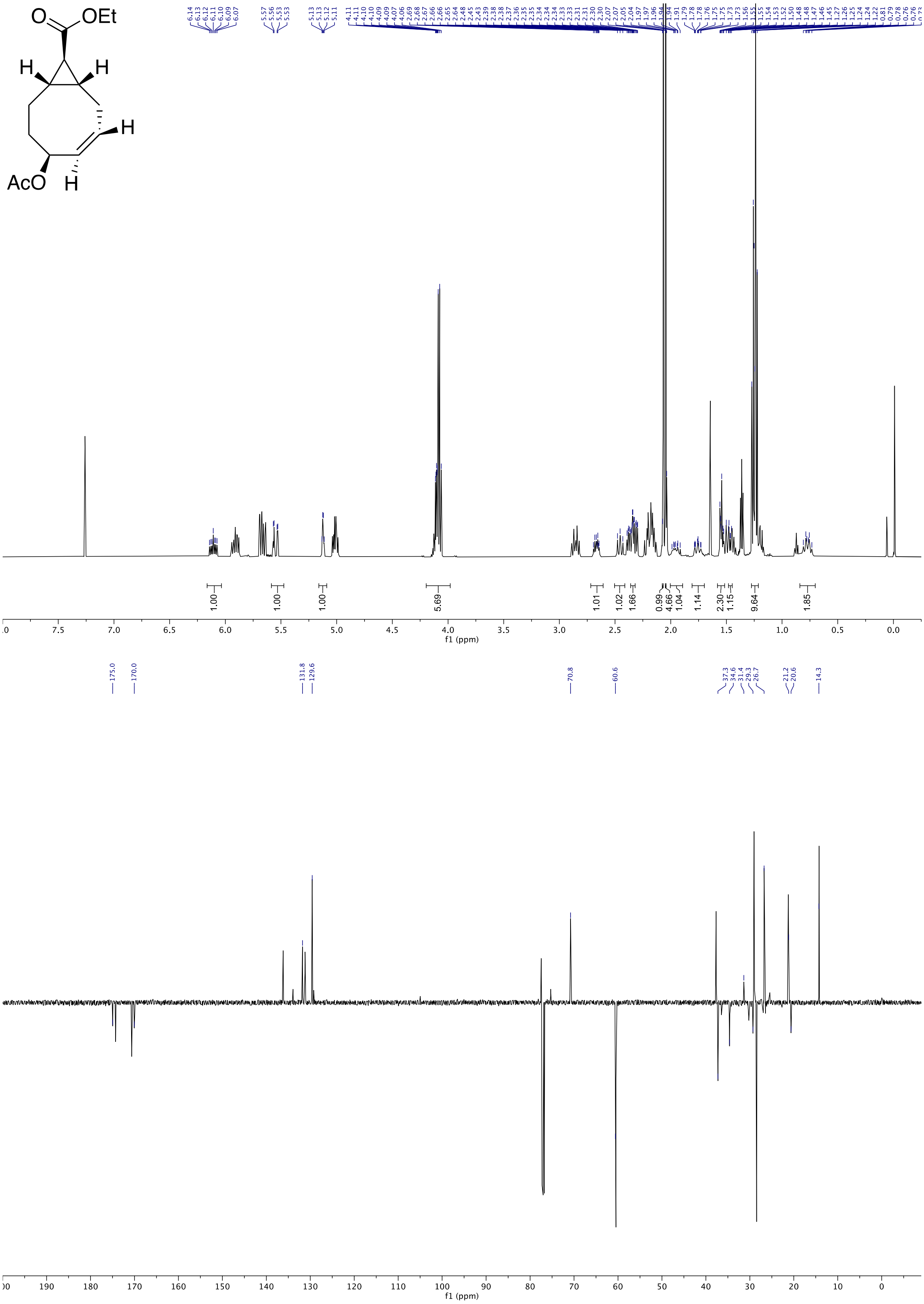

Figure S21. ^{1}H and ^{13}C NMR spectrum of compound **5**.

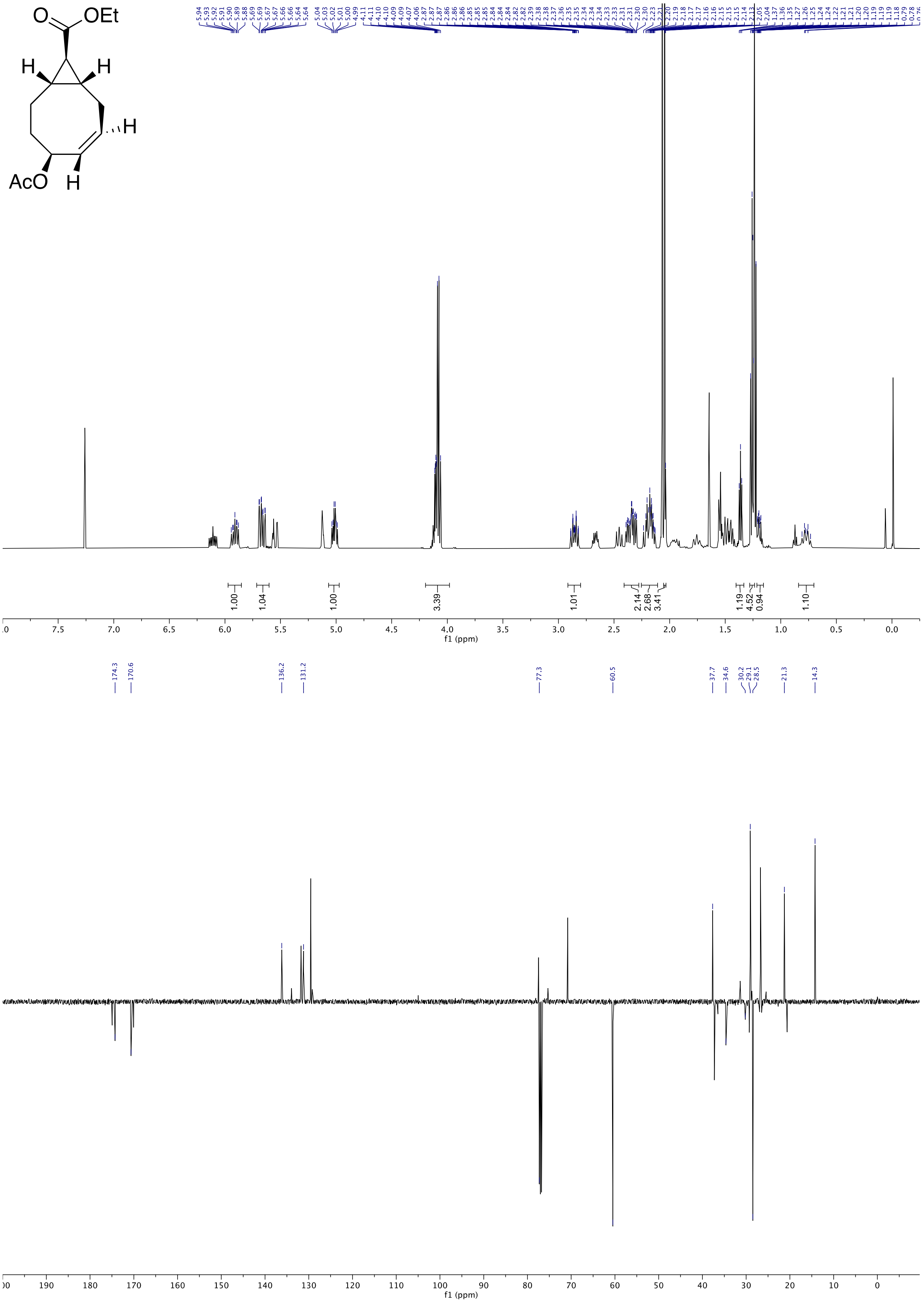

Figure S22. ^{1}H and ^{13}C NMR spectrum of compound **6**.

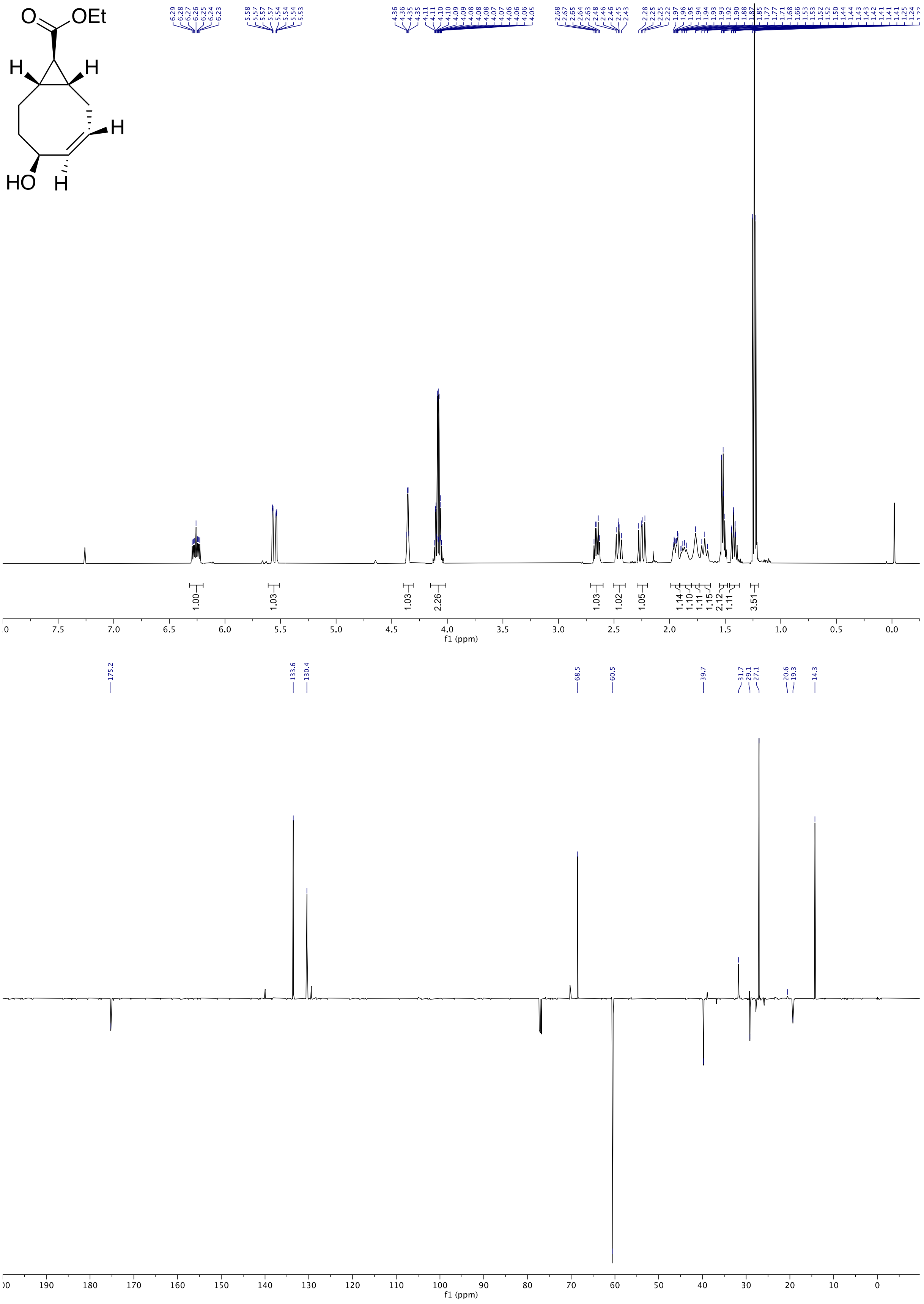

Figure S23. ^{1}H and ^{13}C NMR spectrum of compound **7**.

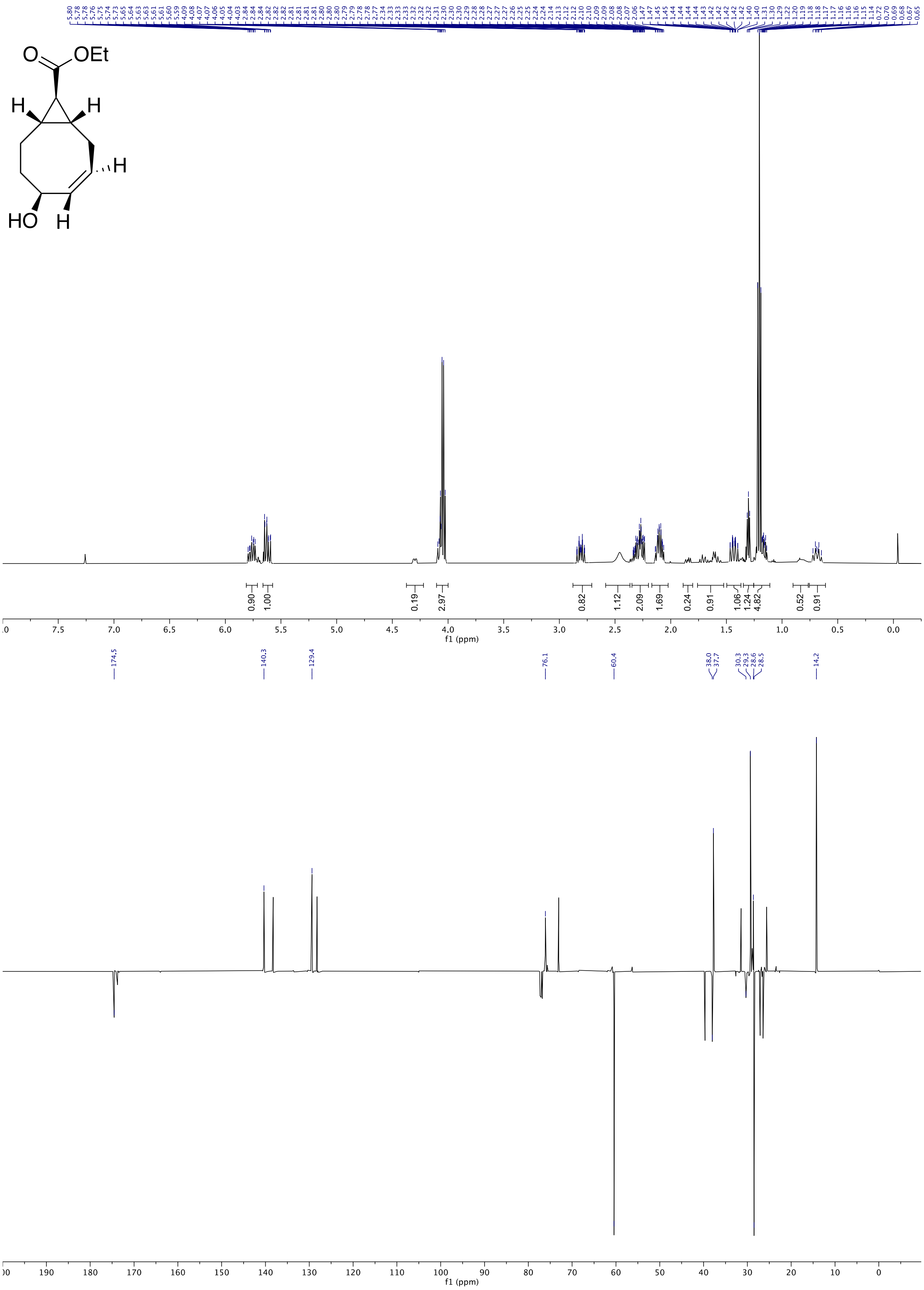

Figure S24. ^{1}H and ^{13}C NMR spectrum of compound **8**.

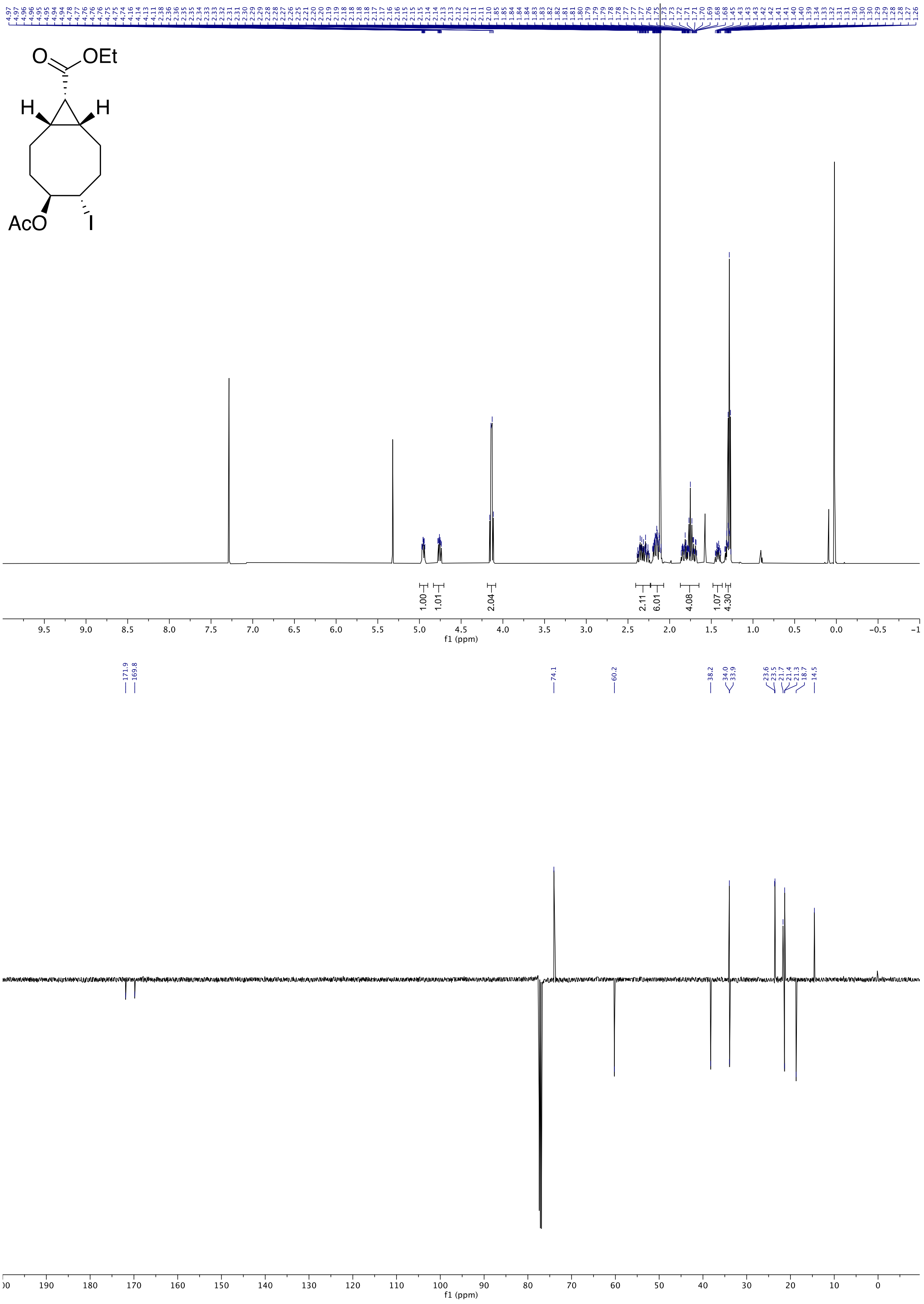

Figure S25. ^{1}H and ^{13}C NMR spectrum of compound **9**.

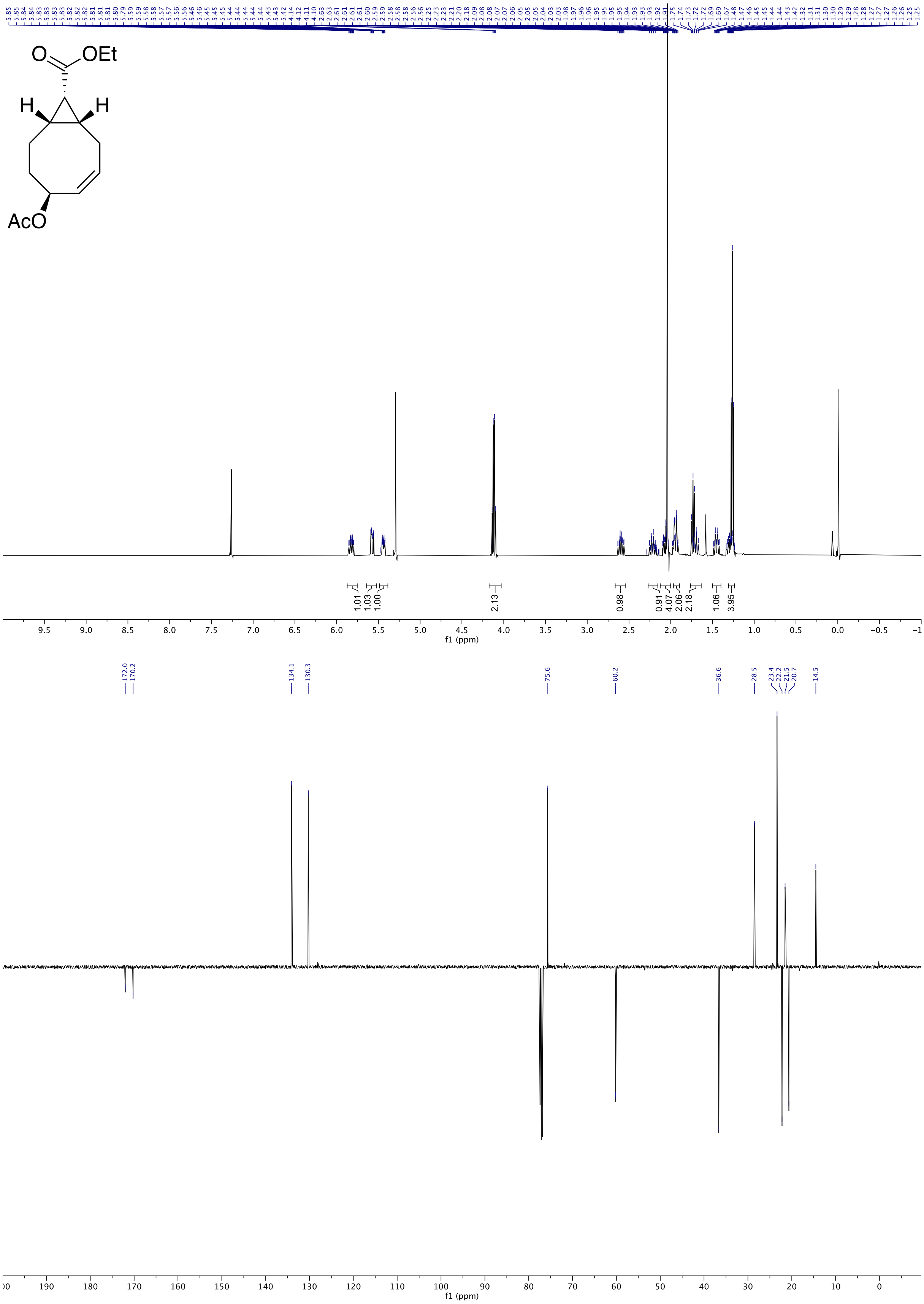

Figure S26. ^{1}H and ^{13}C NMR spectrum of compound **10**.

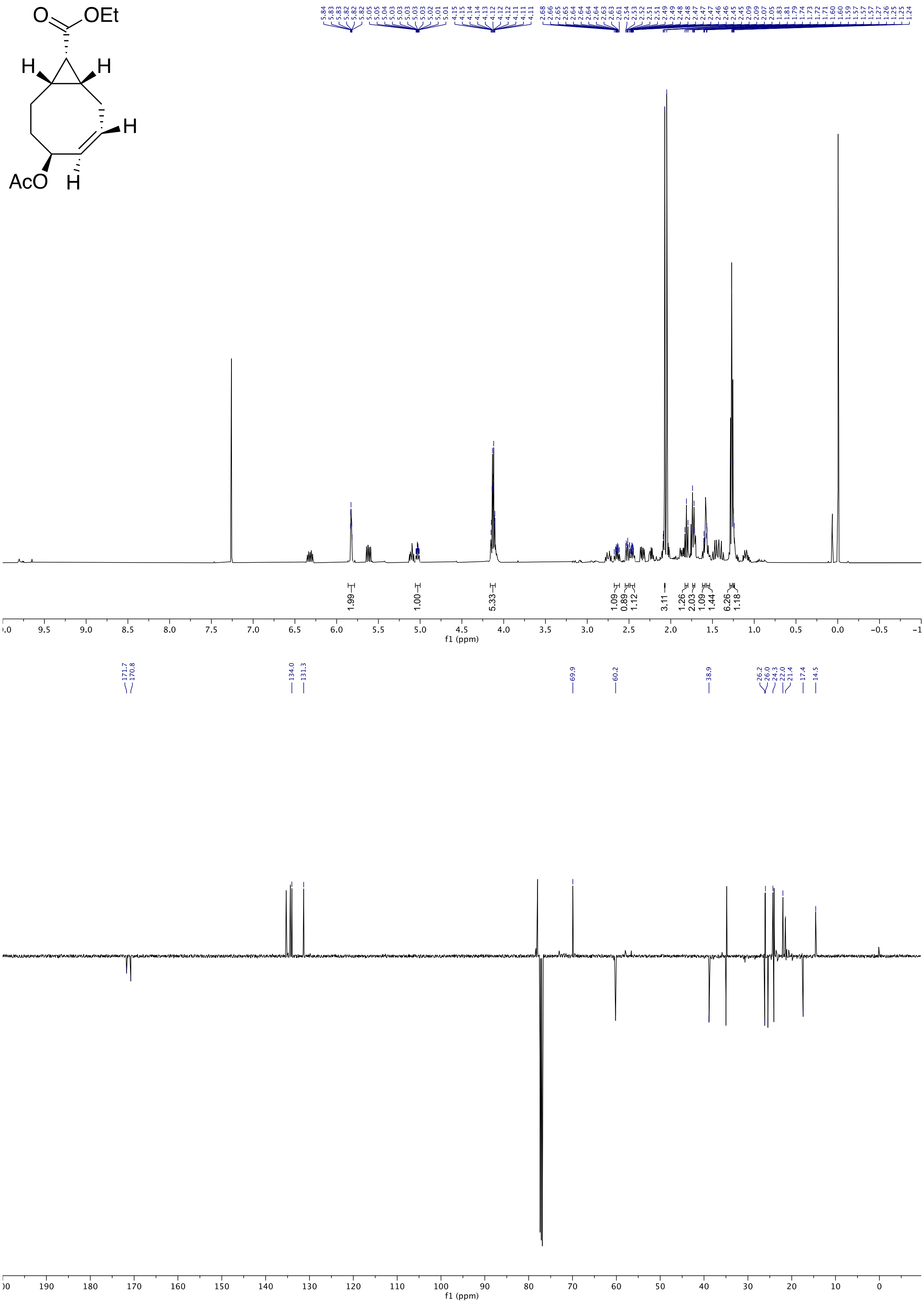

Figure S27. ^{1}H and ^{13}C NMR spectrum of compound **11**.

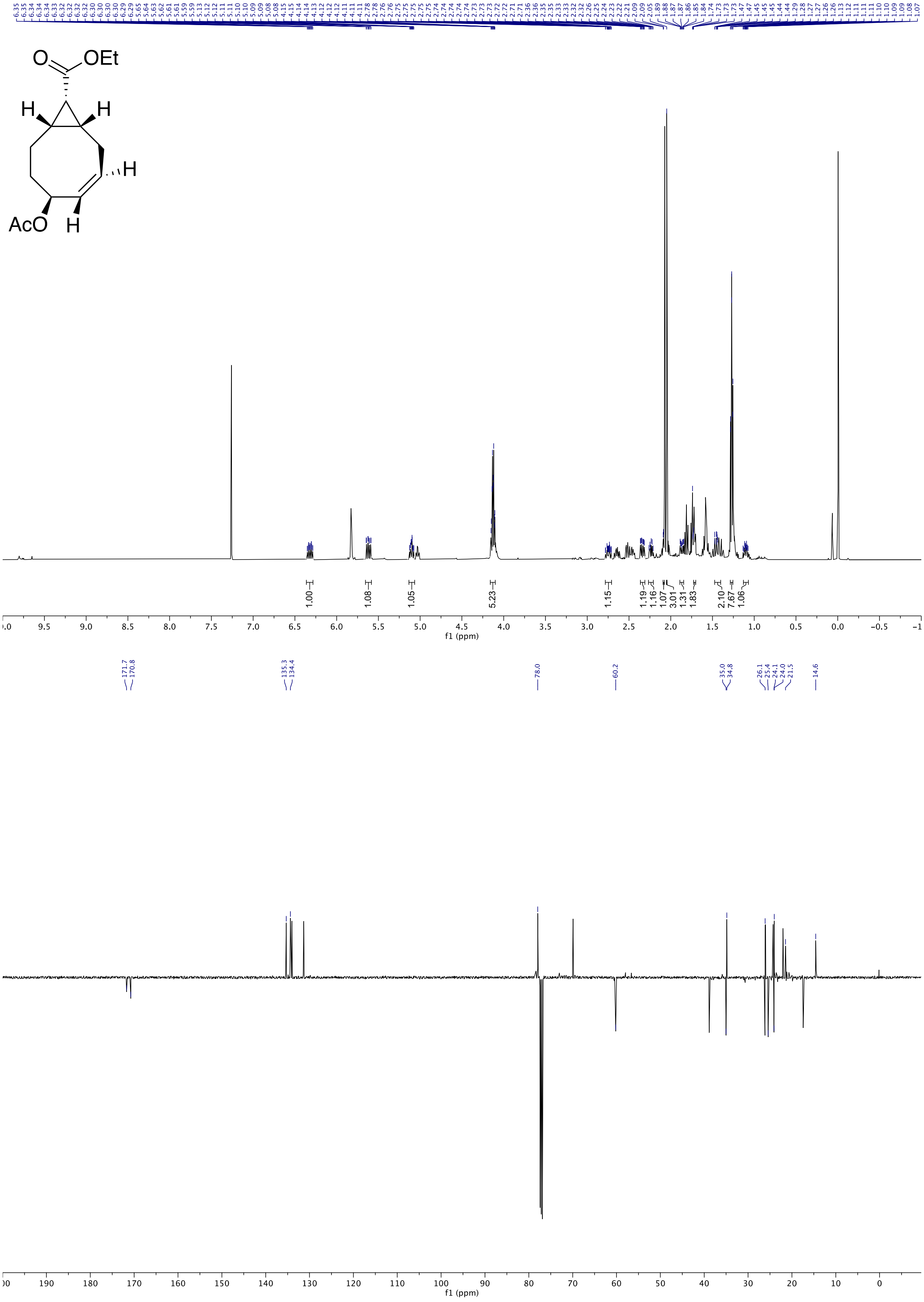

Figure S28. ^{1}H and ^{13}C NMR spectrum of compound **12**.

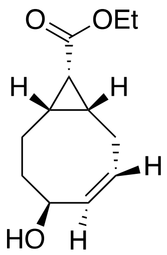

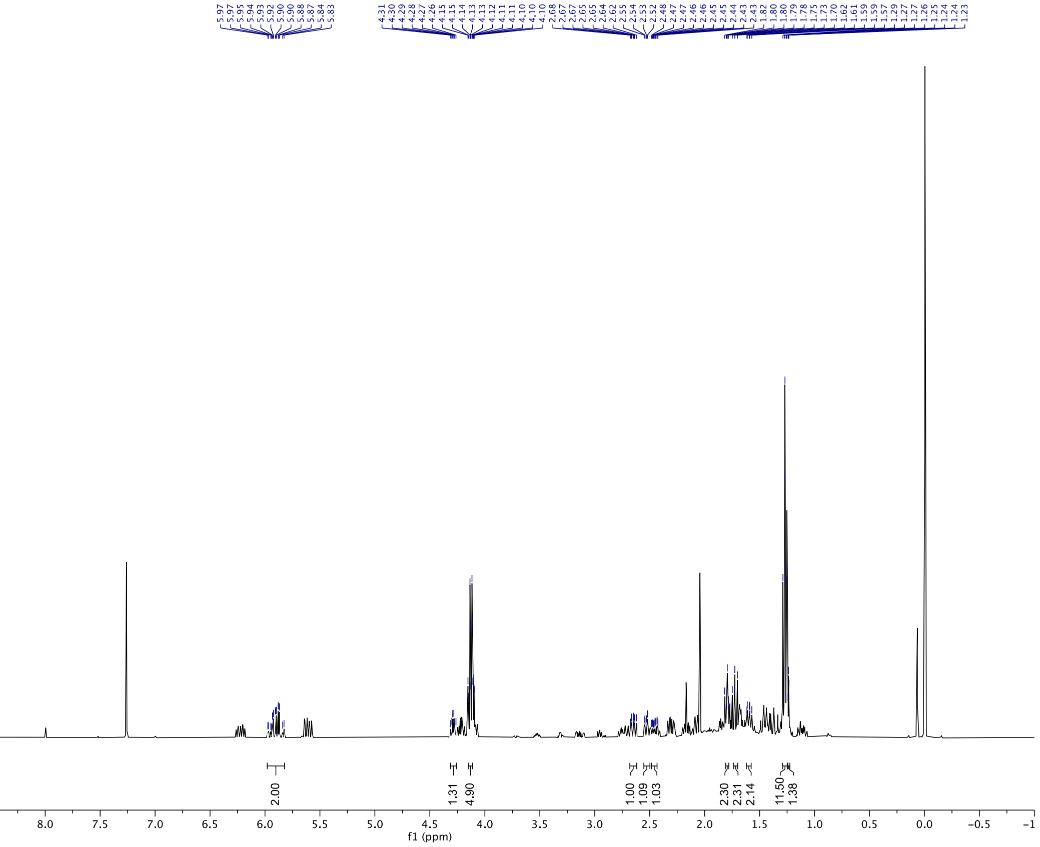

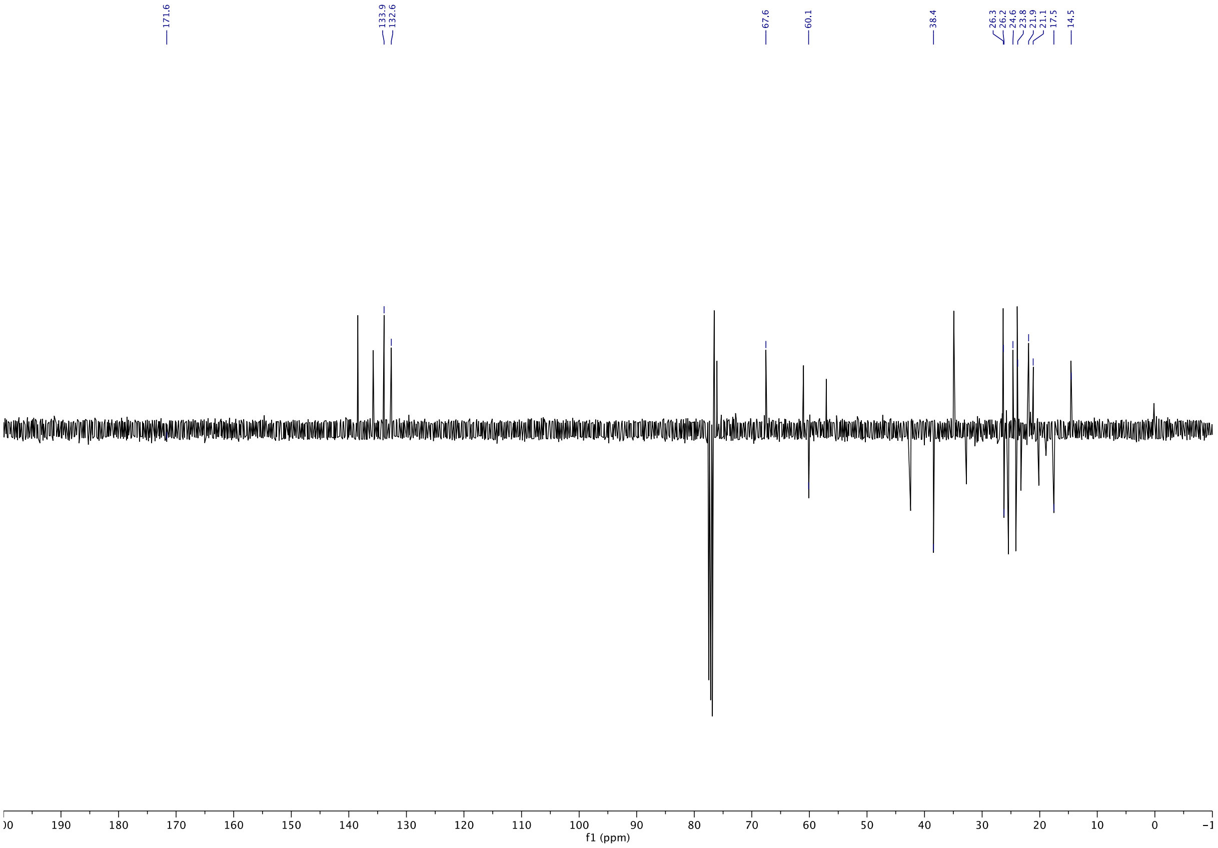

Figure S29. ^{1}H and ^{13}C NMR spectrum of compound **13**.

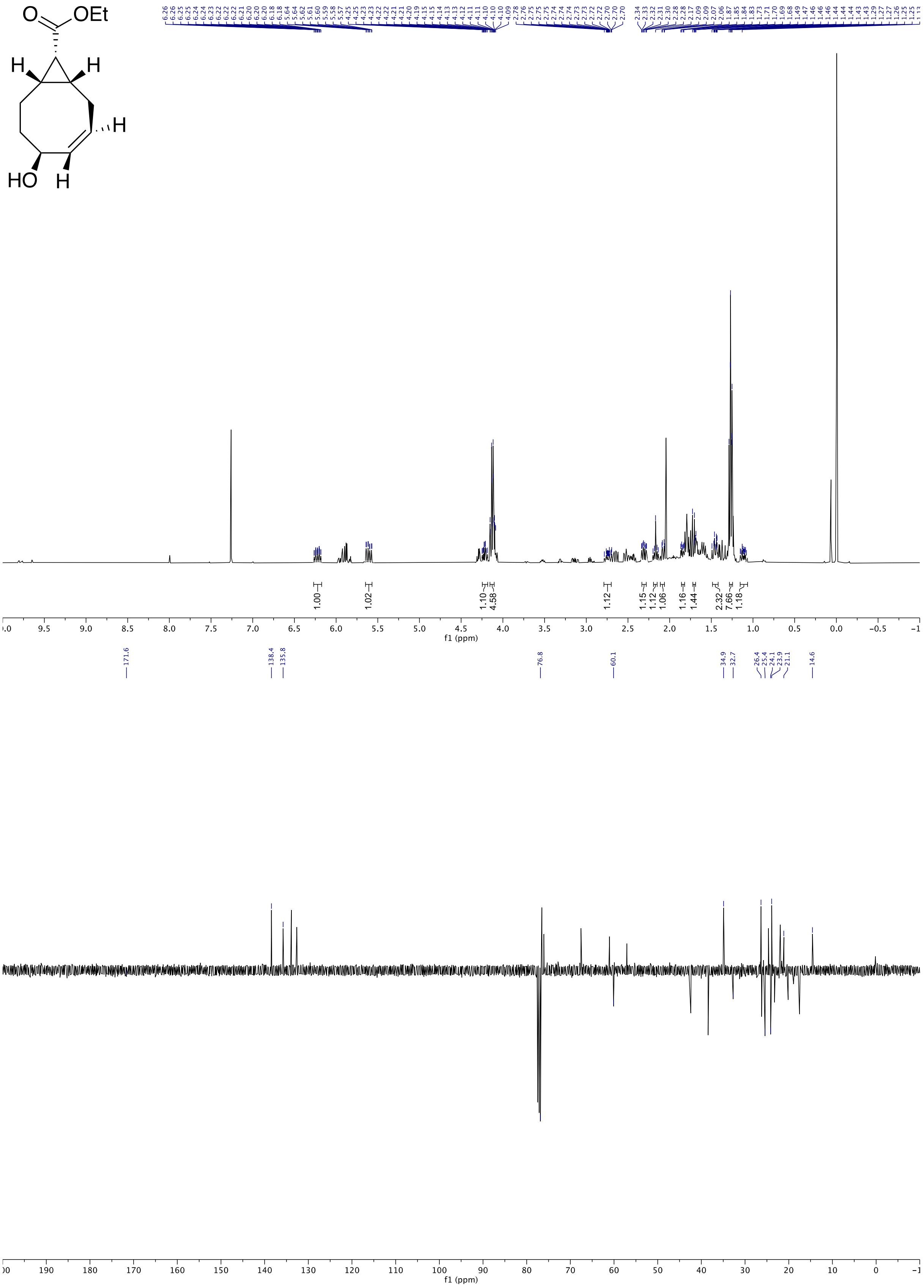

Figure S30. ^{1}H and ^{13}C NMR spectrum of compound **14**.

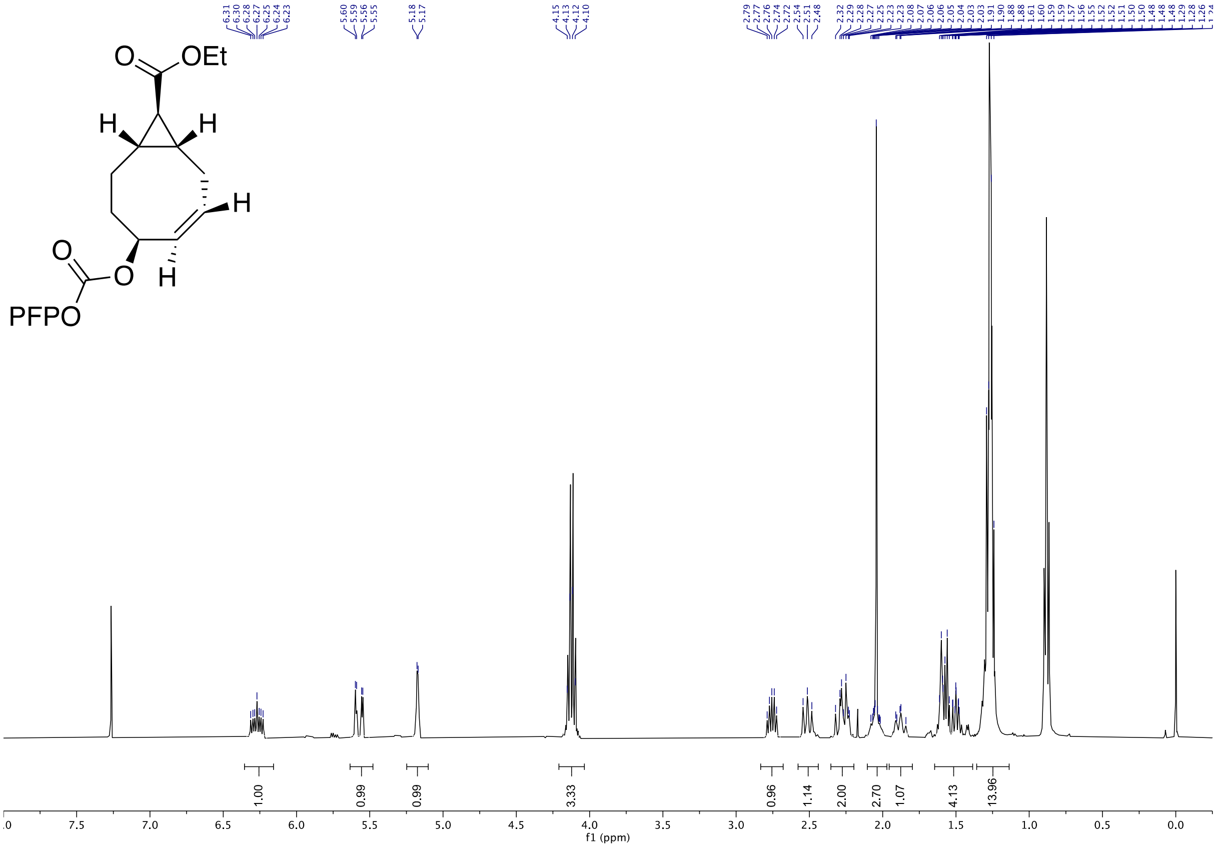
O
OEt
H
H
H
O
O
H
PFPO
1.00
0.99
0.99
3.33
0.96
1.14
2.00
2.70
1.07
4.13
13.96
f1 (ppm)

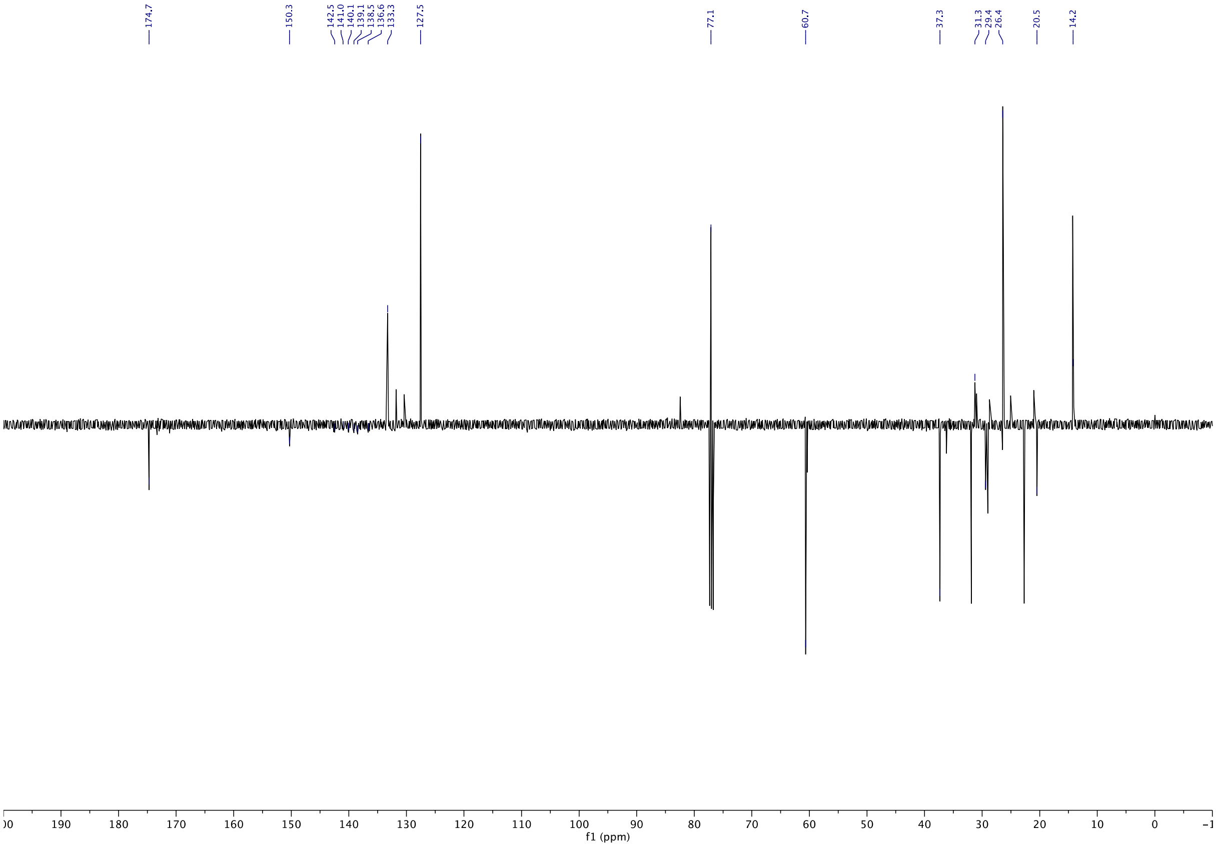
174.7
150.3
142.5
141.0
140.1
139.1
138.5
136.6
133.3
127.5
77.1
60.7
37.3
31.3
29.4
26.4
20.5
14.2
f1 (ppm)

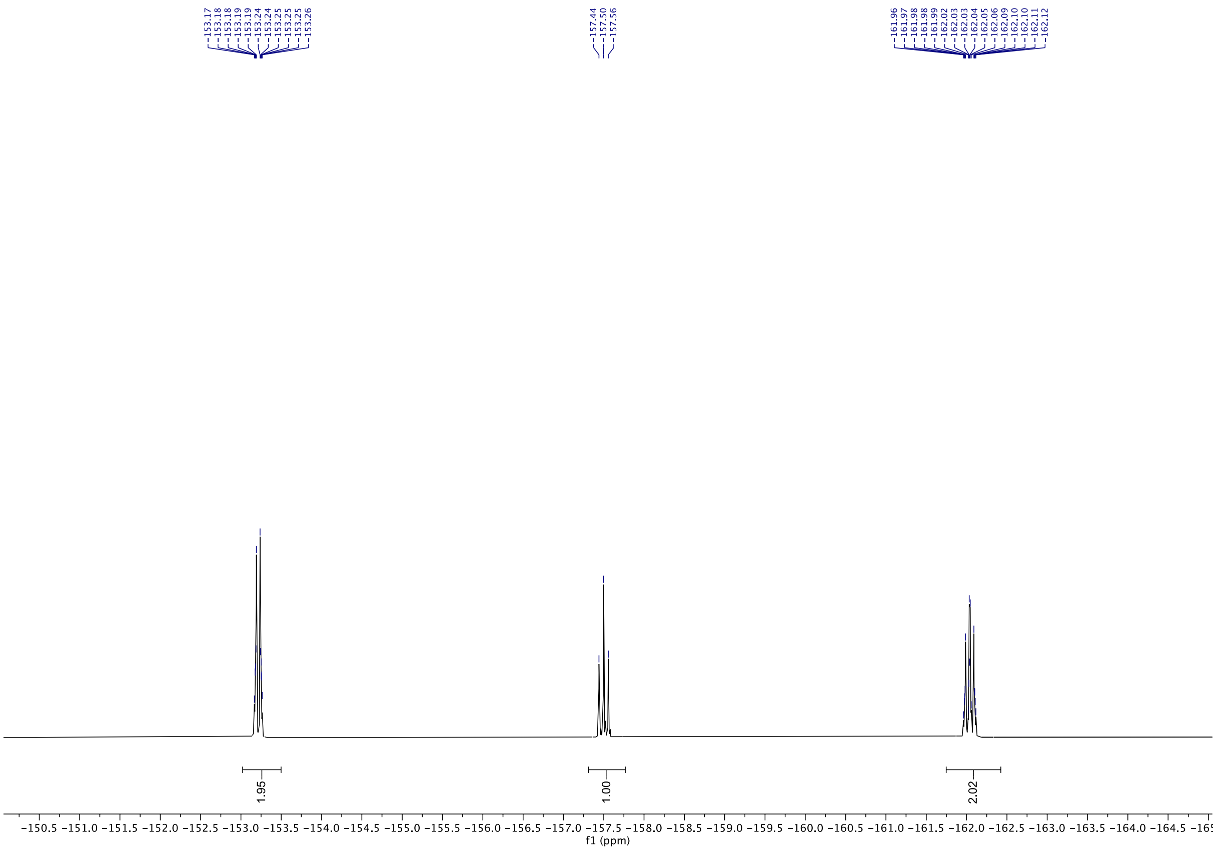

Figure S31. ^{1}H,^{13}C and ^{19}F NMR spectrum of compound **15**.

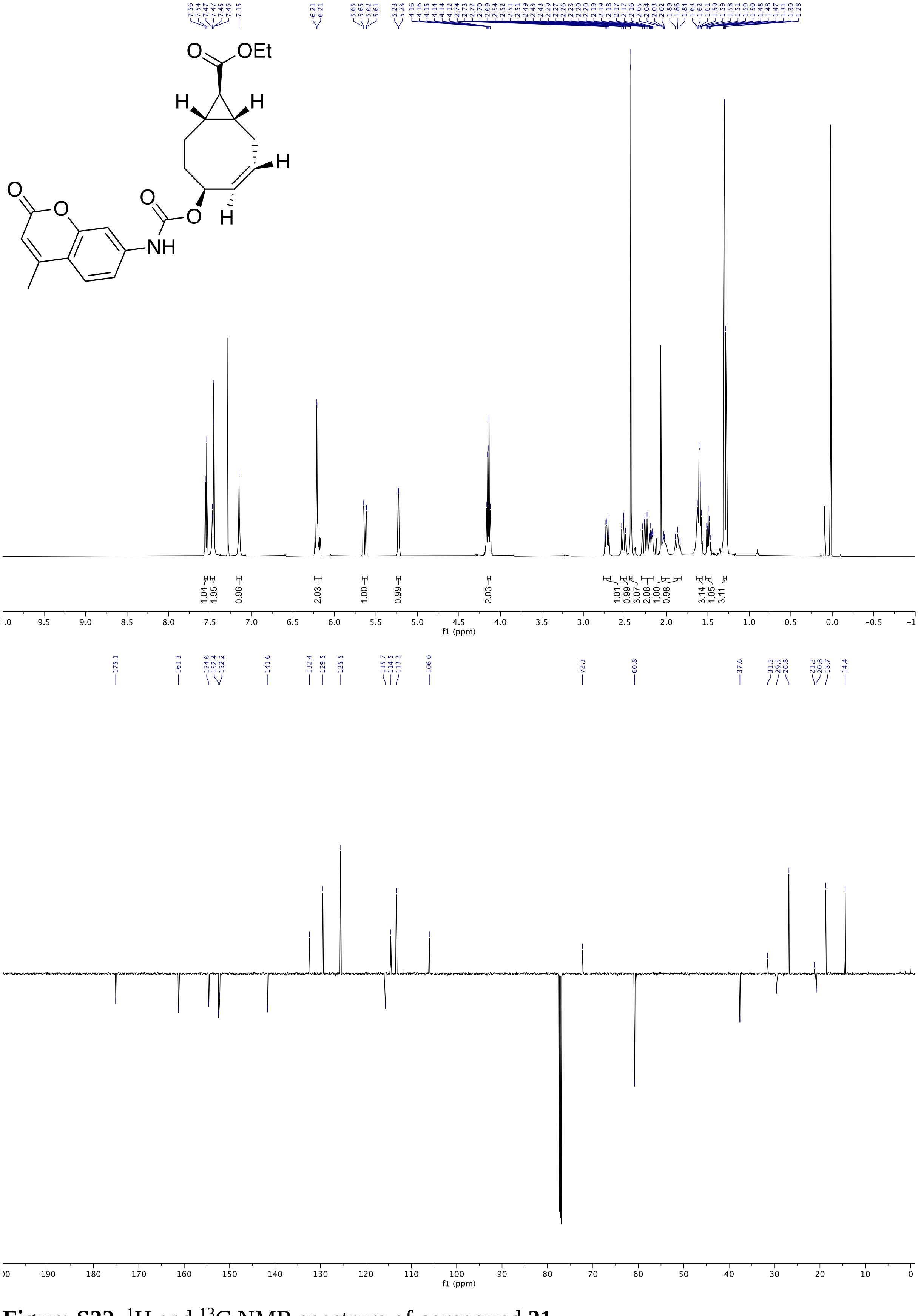

Figure S32. ^{1}H and ^{13}C NMR spectrum of compound **21**.

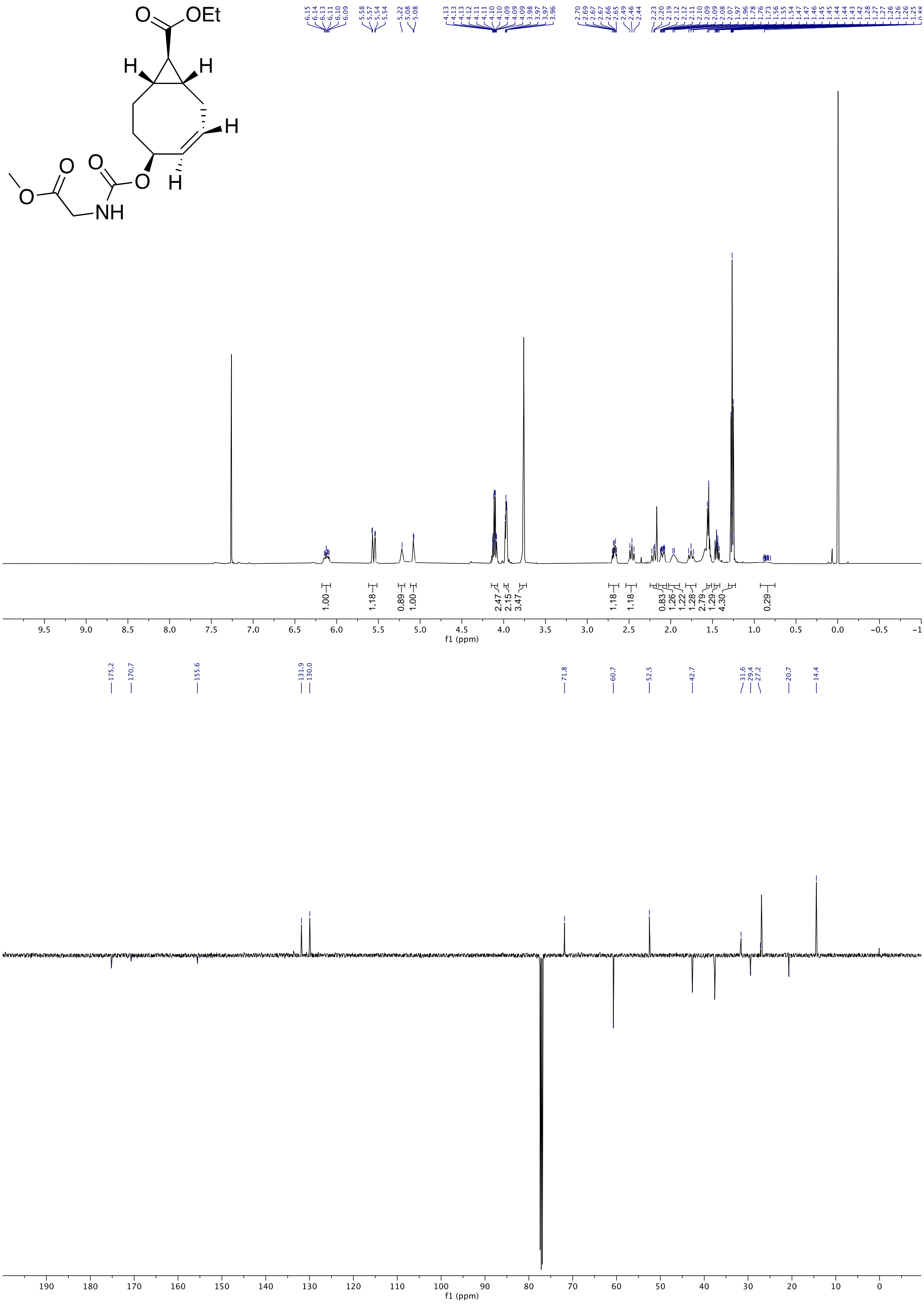

Figure S33. ^{1}H and ^{13}C NMR spectrum of compound **23**.

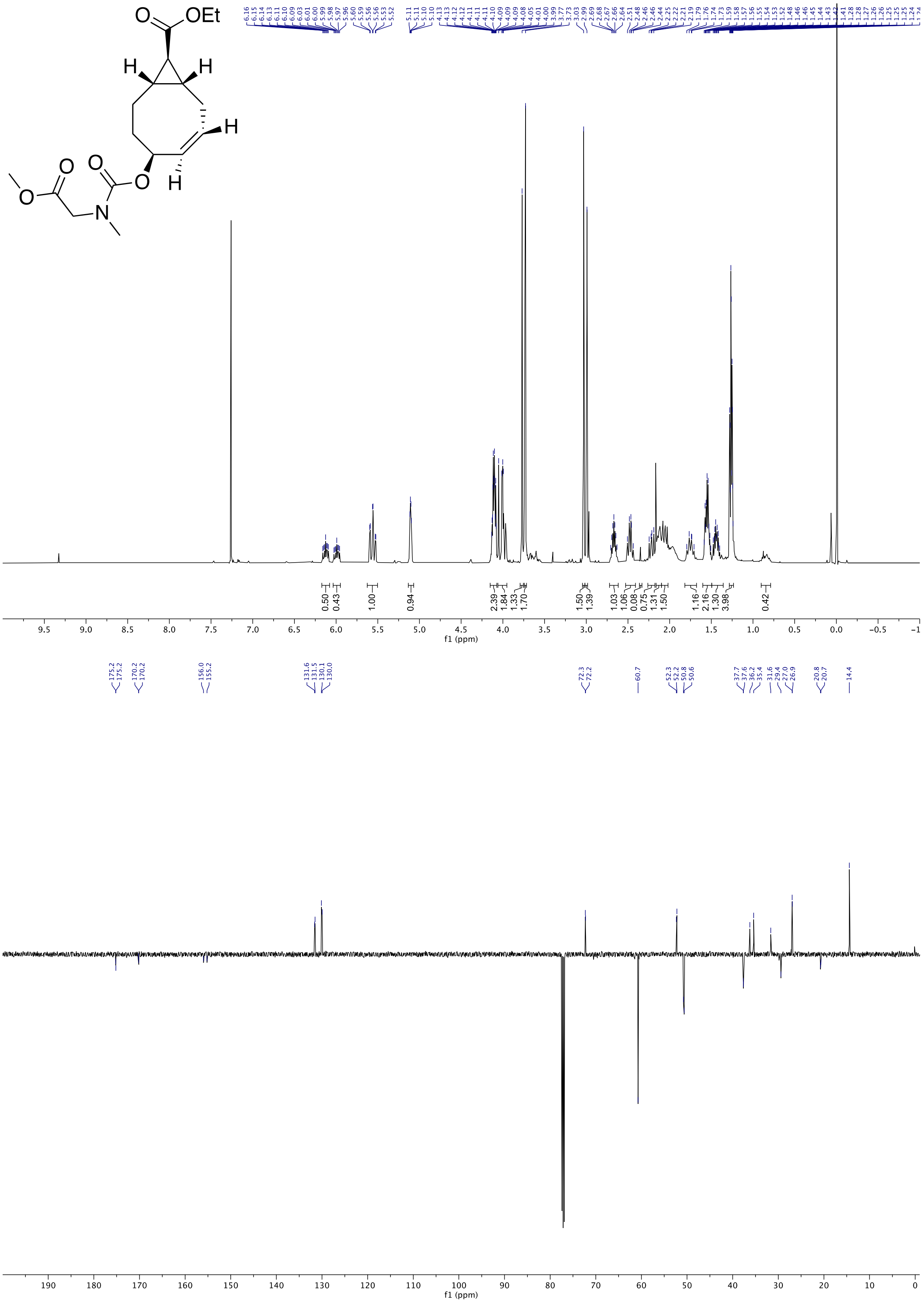

Figure S34. ^{1}H and ^{13}C NMR spectrum of compound **25**.

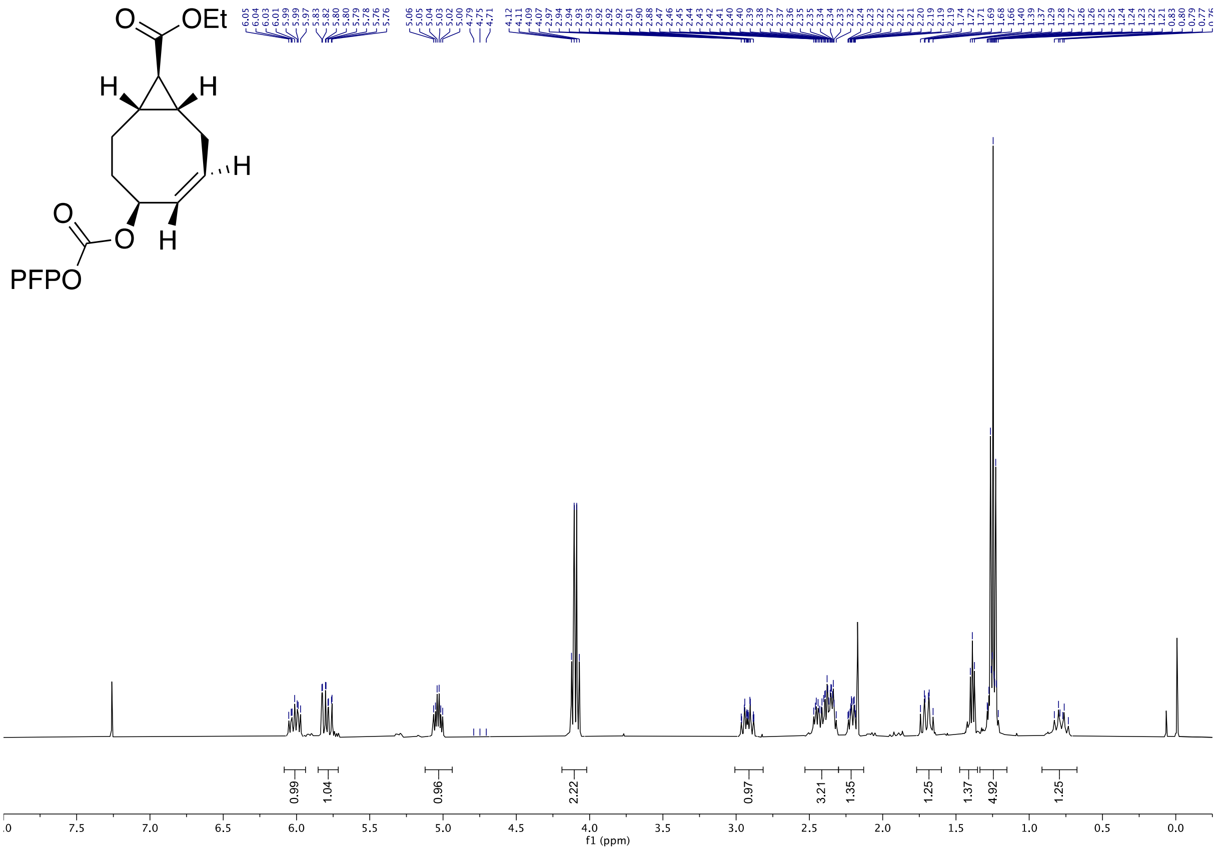
OEt
PFPO
0.99
1.04
0.96
2.22
0.97
3.21
1.35
1.25
1.37
4.92
1.25
f1 (ppm)

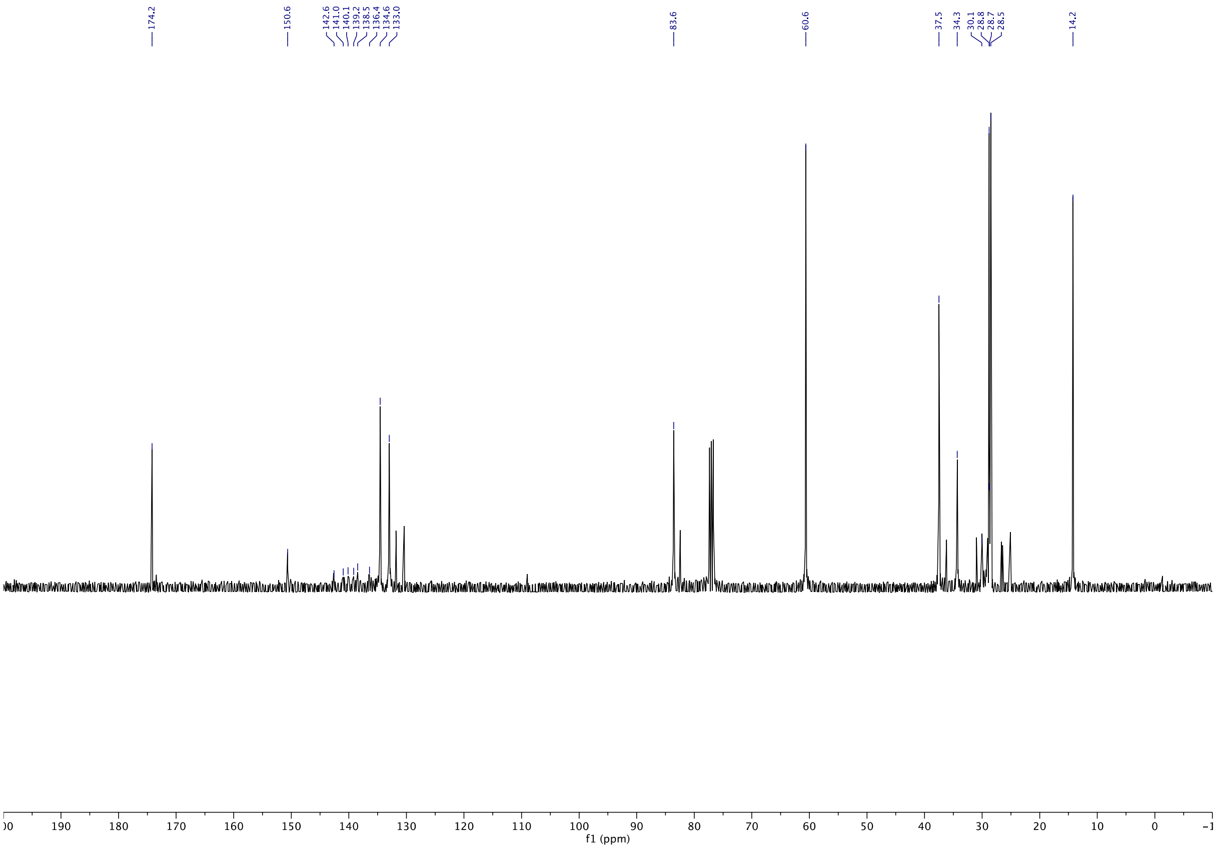
174.2
150.6
142.6
141.0
140.1
139.2
138.5
136.4
134.6
133.0
83.6
60.6
37.5
34.3
30.1
28.8
28.7
28.5
14.2
f1 (ppm)

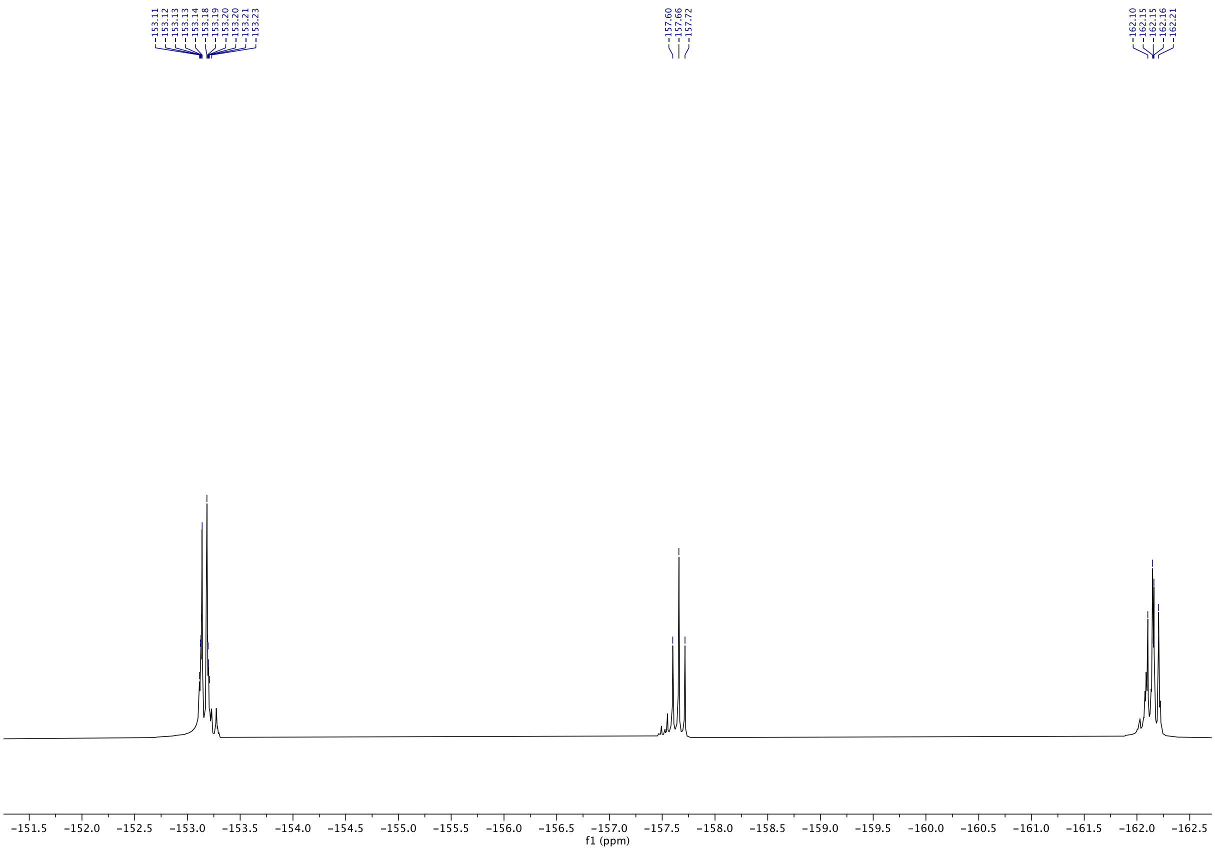

Figure S35. ^{1}H, ^{13}C and ^{19}F NMR spectrum of compound **16**.

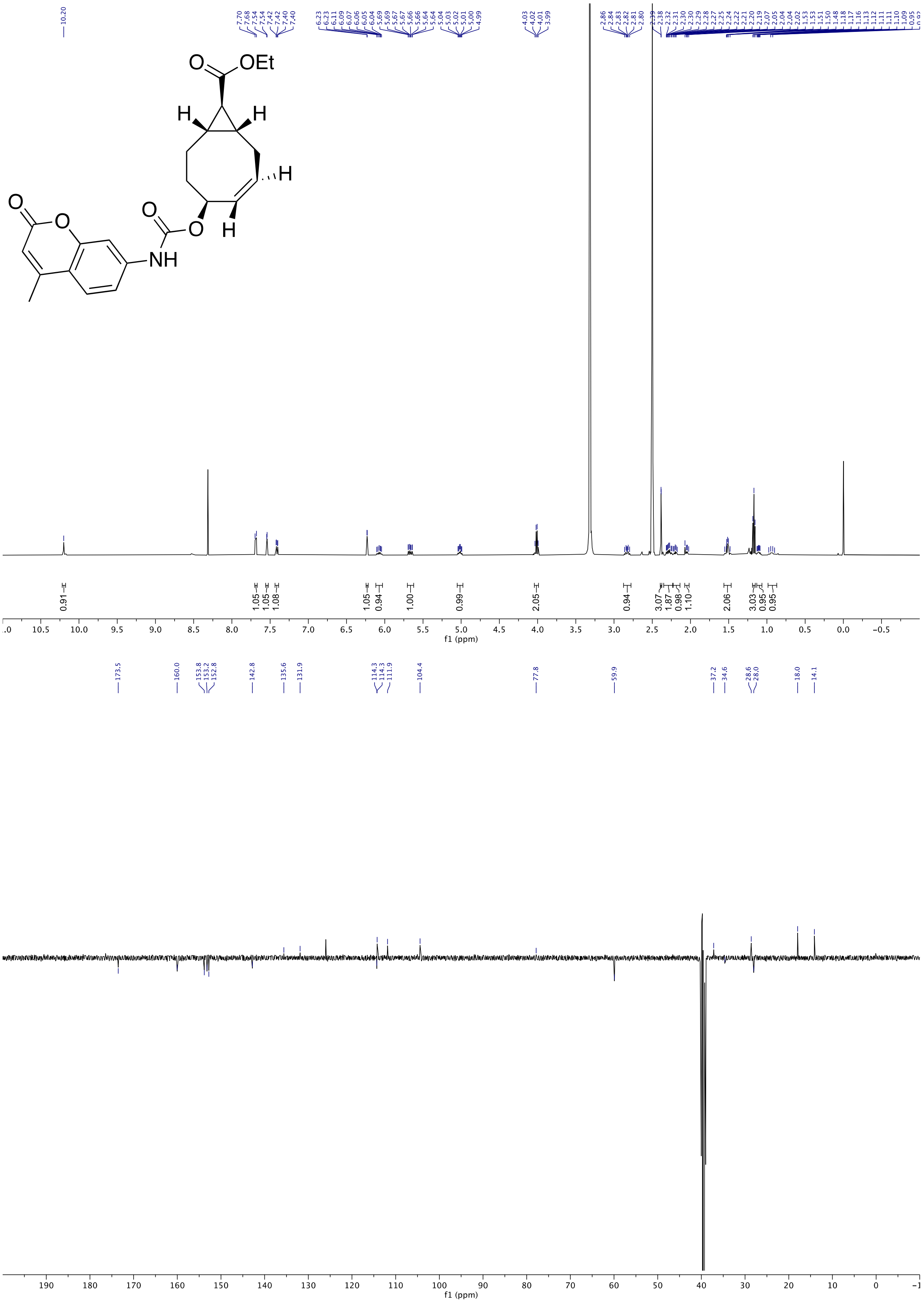

Figure S36. ^{1}H and ^{13}C NMR spectrum of compound **22**.

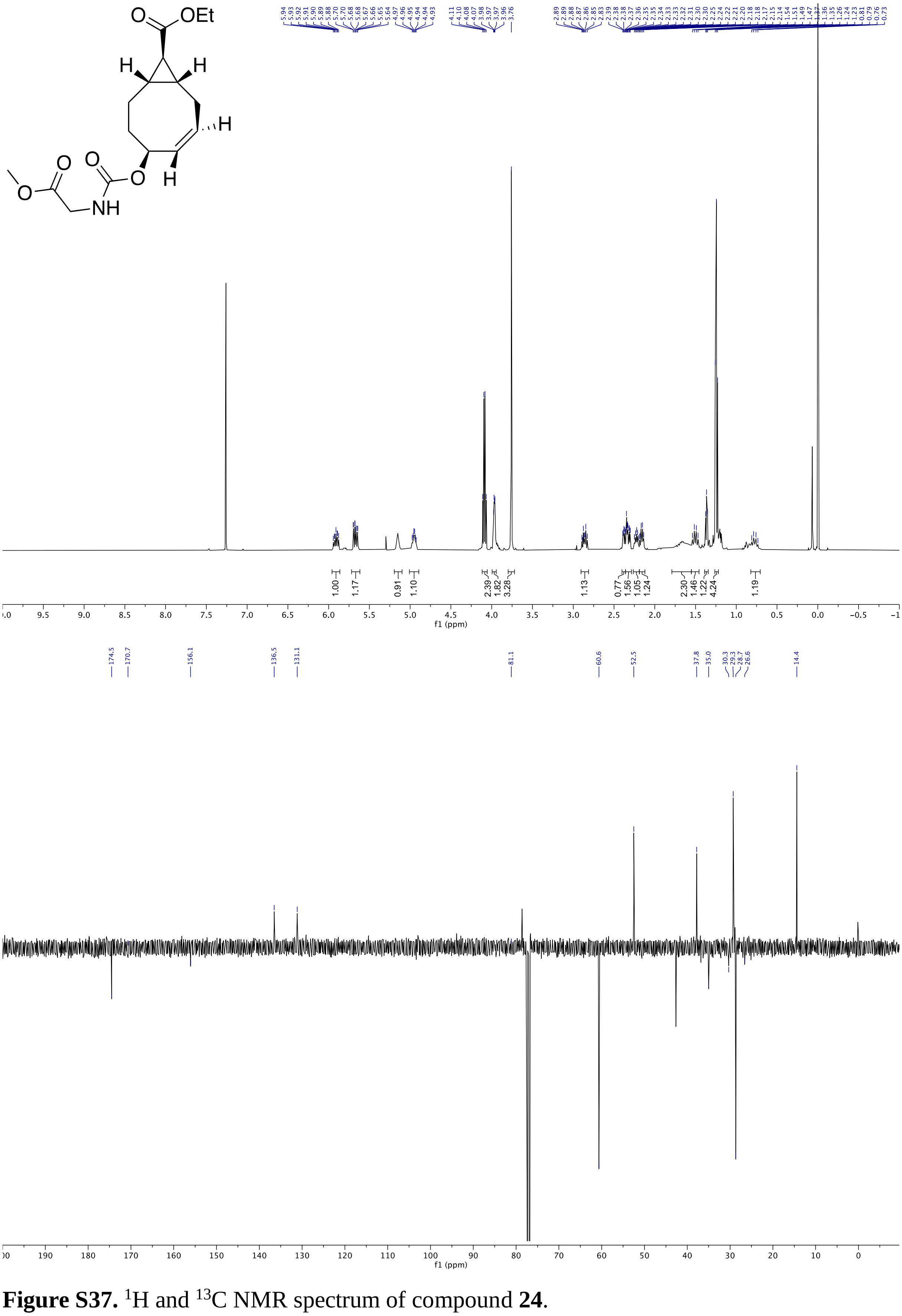

Figure S37. ^{1}H and ^{13}C NMR spectrum of compound **24**.

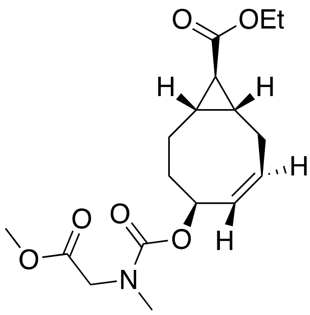

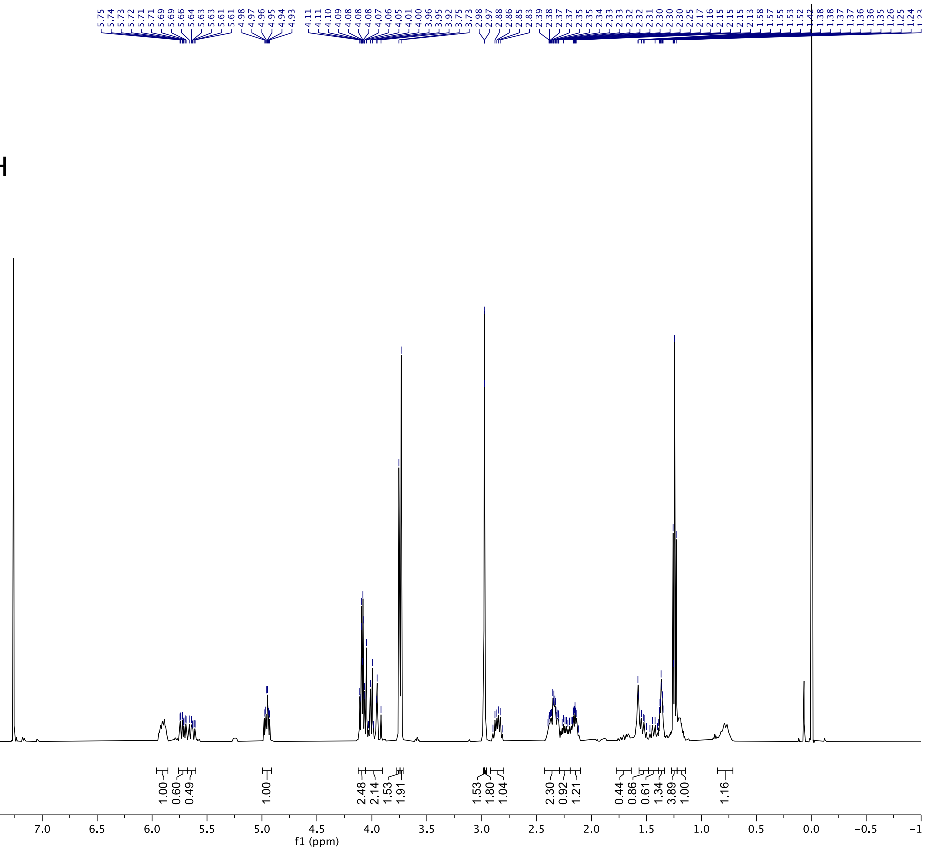

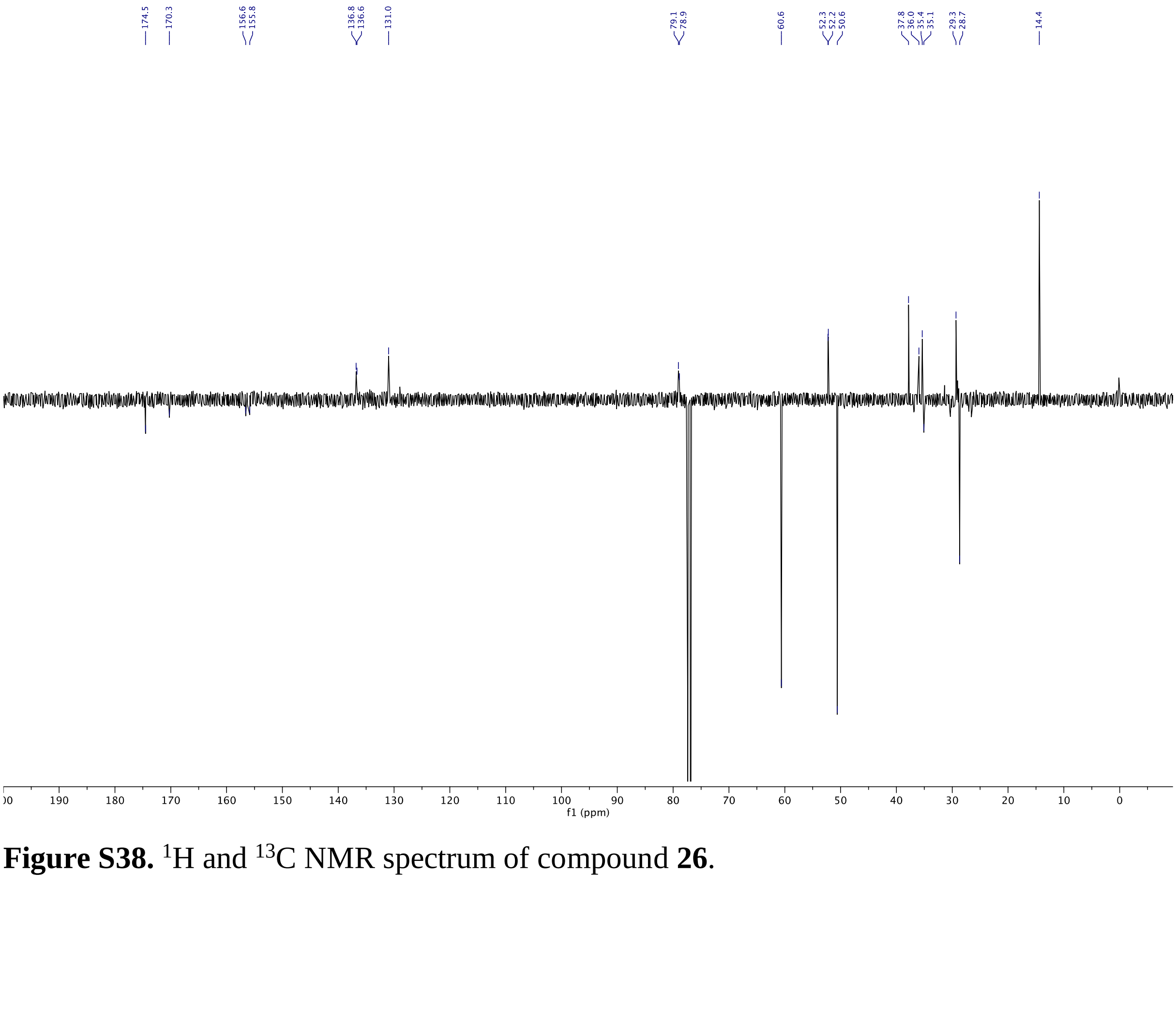

Figure S38. ^{1}H and ^{13}C NMR spectrum of compound **26**.

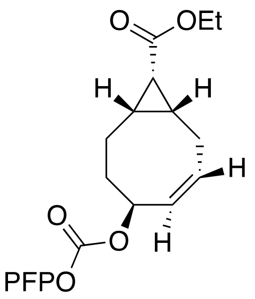

OEt
O
H
H
H
O
O
H
PFPO

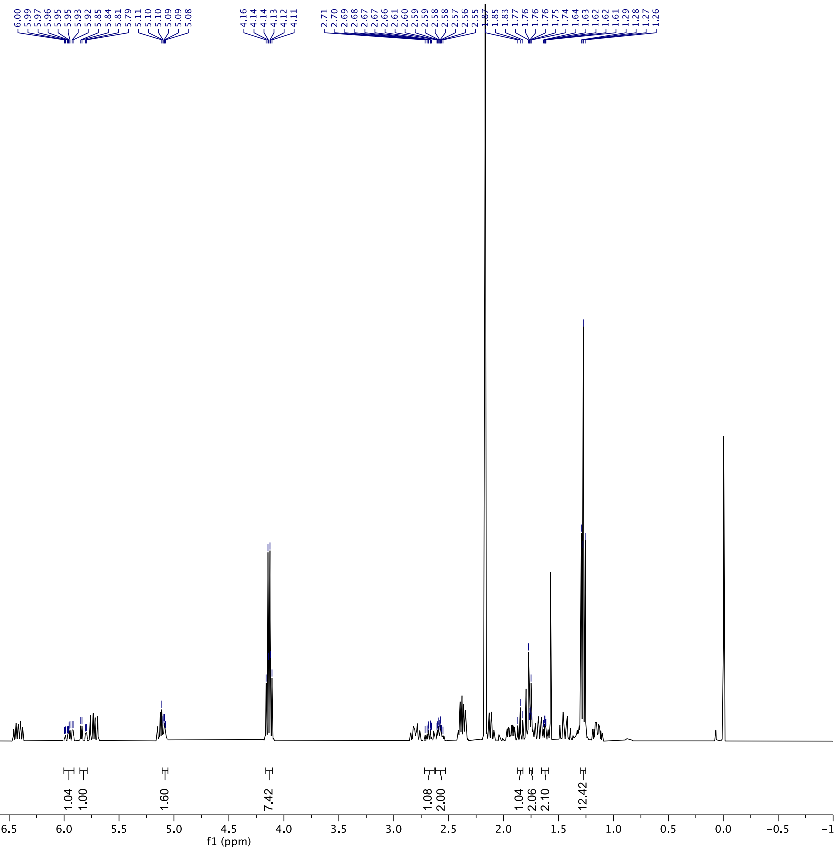

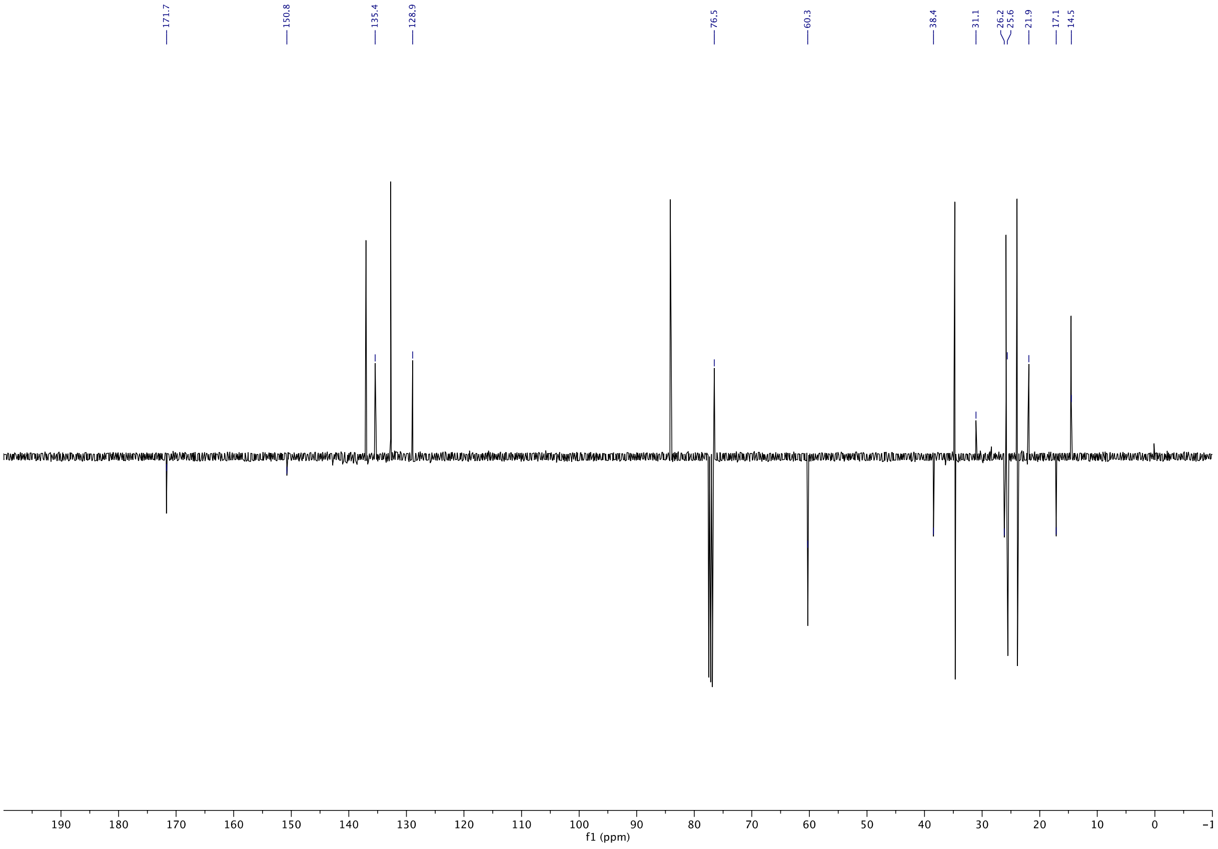

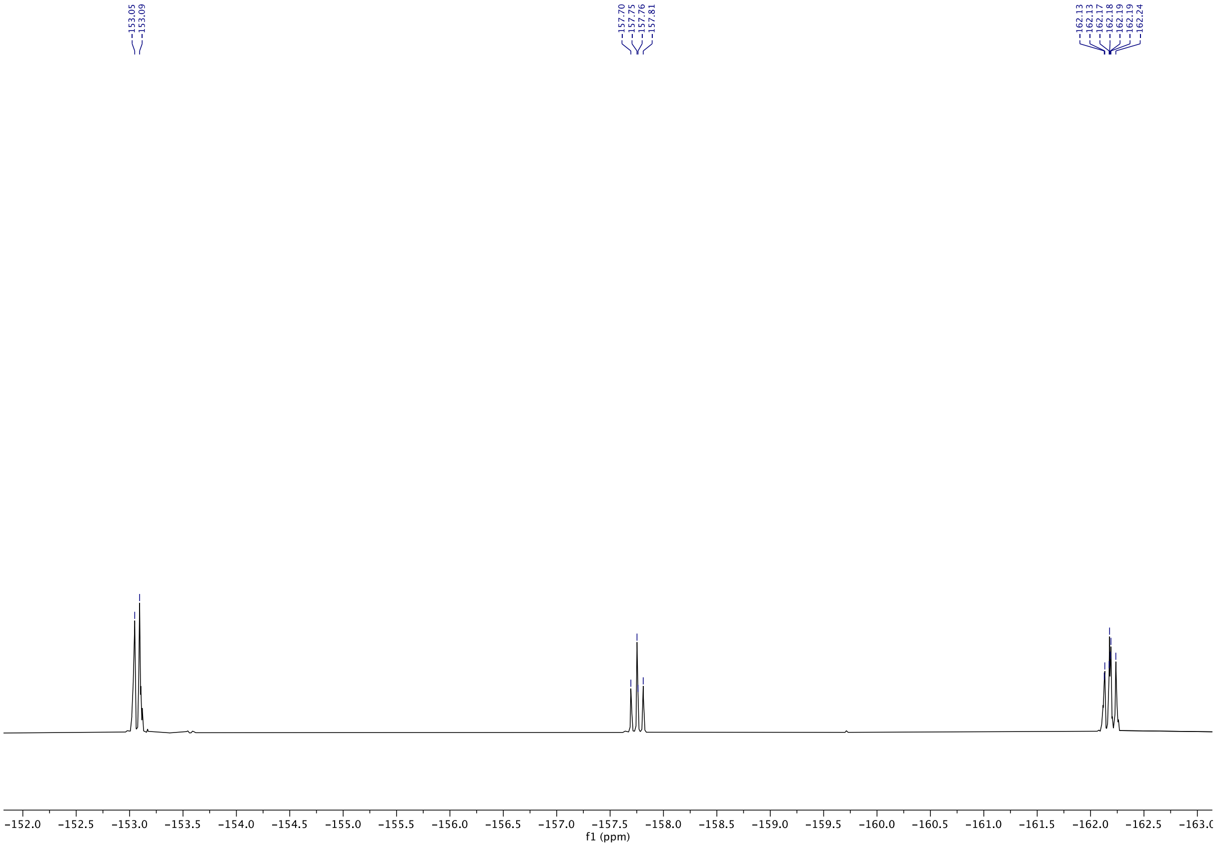

Figure S39. ^{1}H, ^{13}C and ^{19}F spectrum of compound **17**.

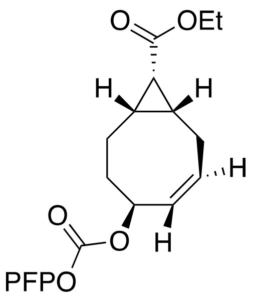
O
OEt
H
H
H
O
O
H
PFPO

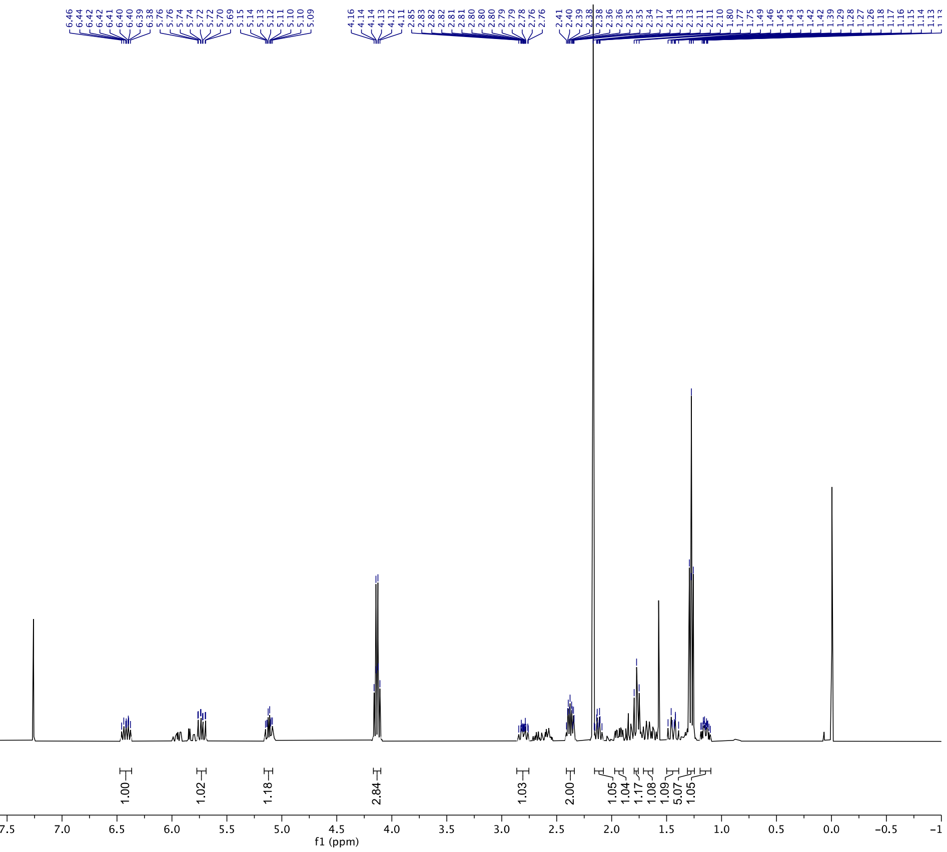
1.00
1.02
1.18
2.84
1.03
2.00
1.05
1.04
1.17
1.08
1.09
5.07
1.05
f1 (ppm)

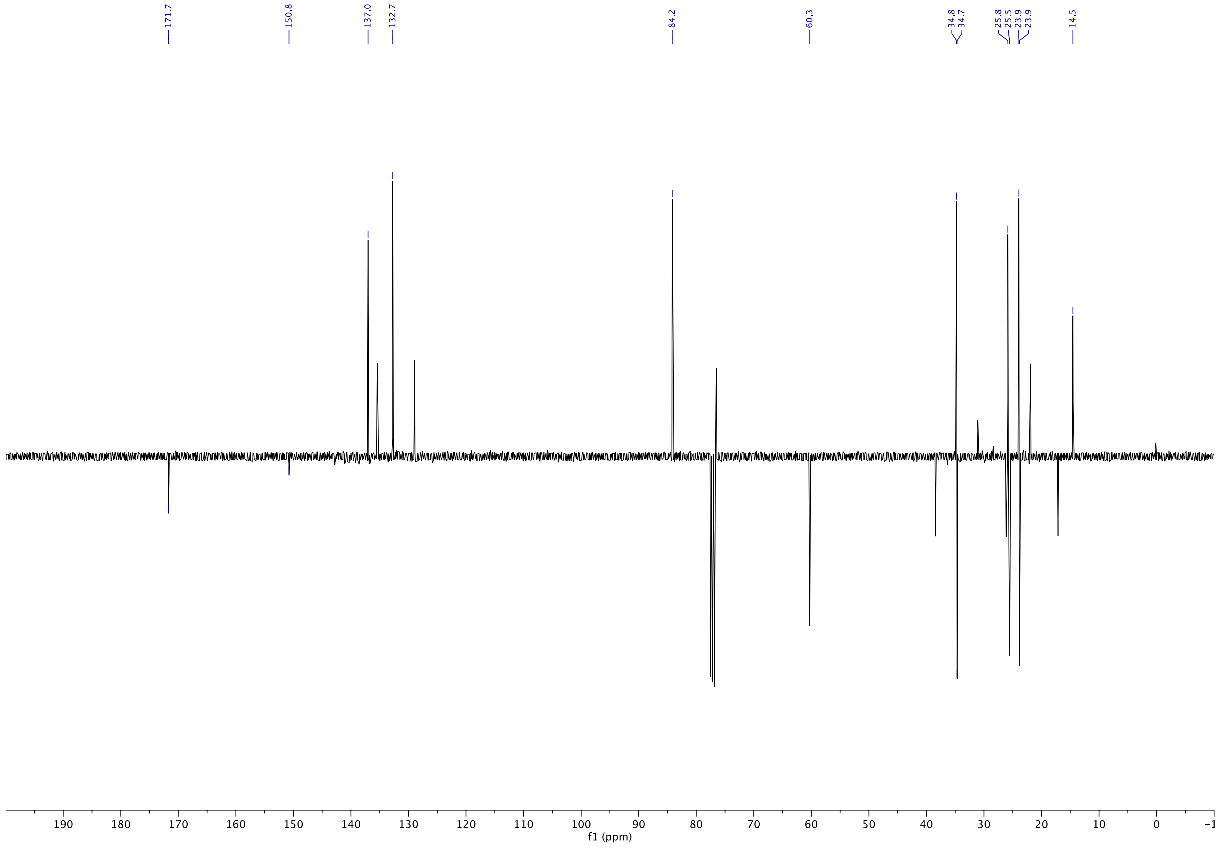
171.7
150.8
137.0
132.7
84.2
60.3
34.8
34.7
25.8
25.5
23.9
23.9
14.5
f1 (ppm)

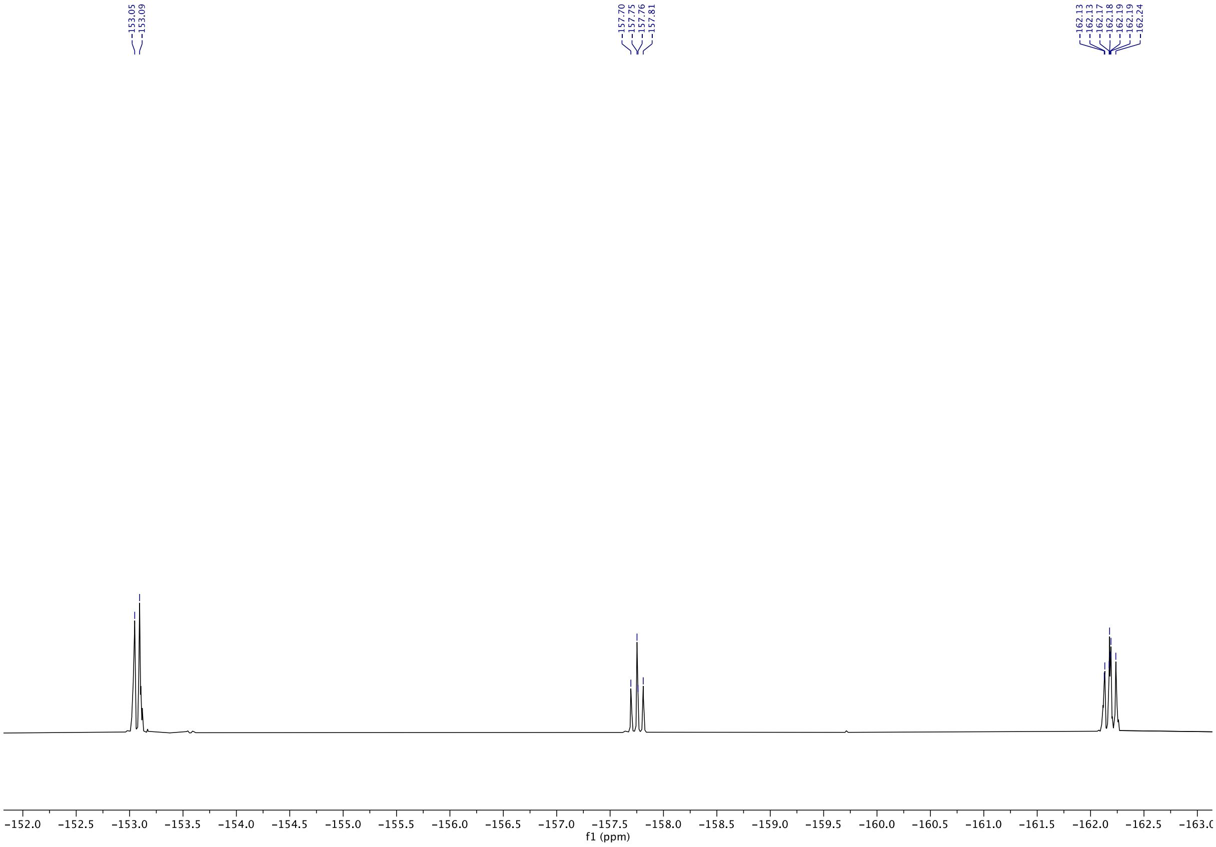

Figure S40. ^{1}H, ^{13}C and ^{19}F NMR spectrum of compound **18**.

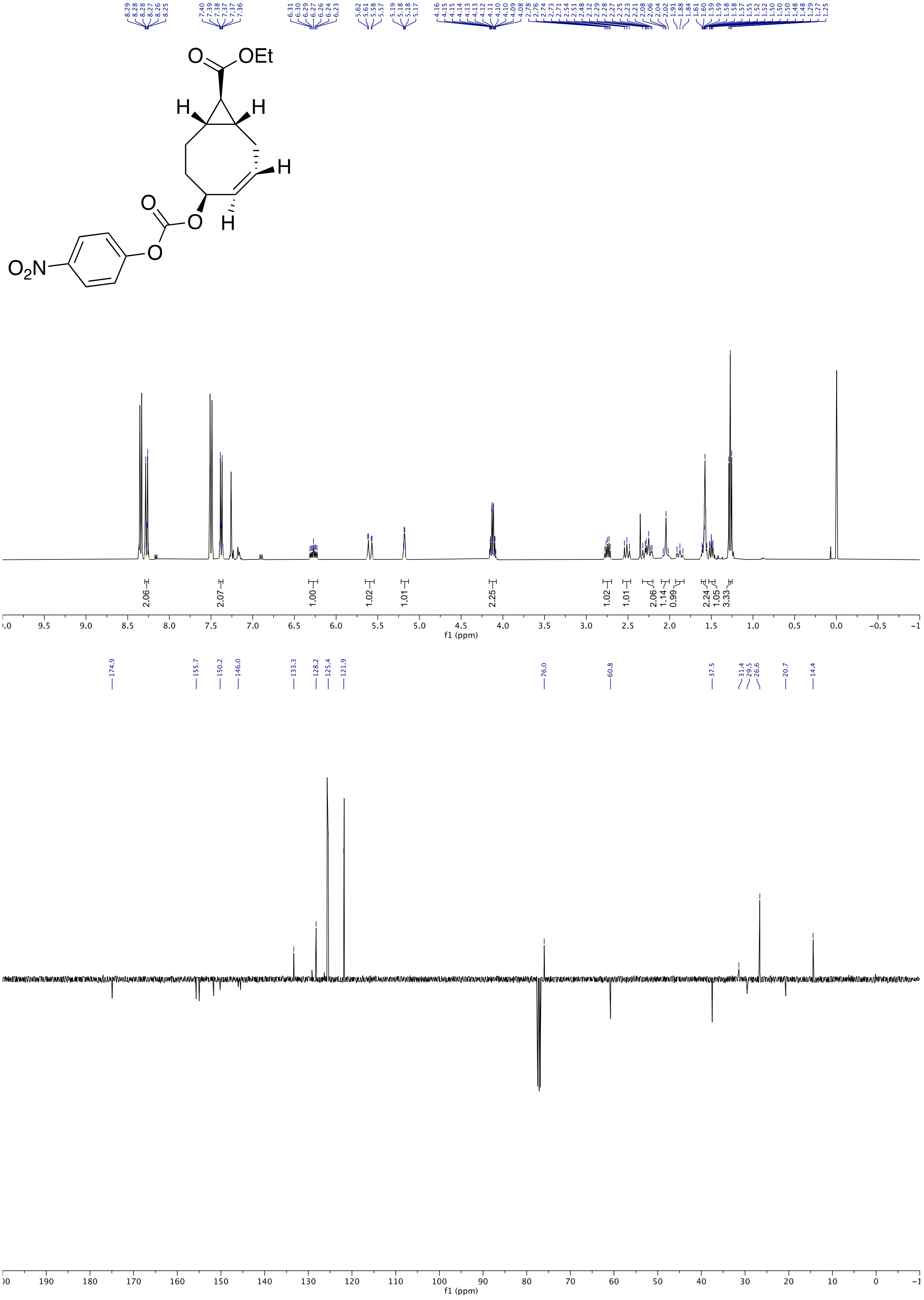

Figure S41. ^{1}H and ^{13}C NMR spectrum of compound **28**.

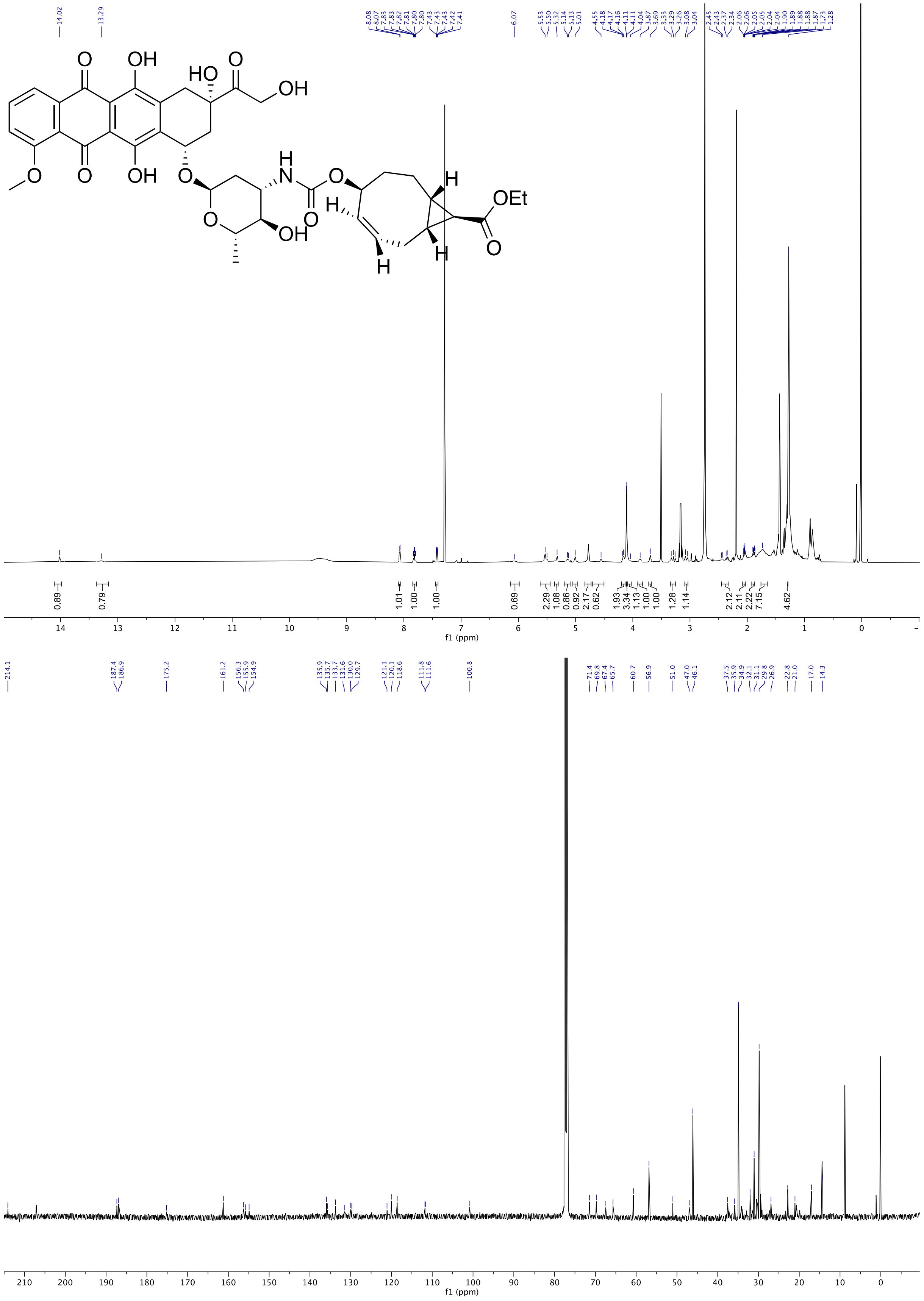

Figure S42. ^{1}H and ^{13}C NMR spectrum of compound **27**.

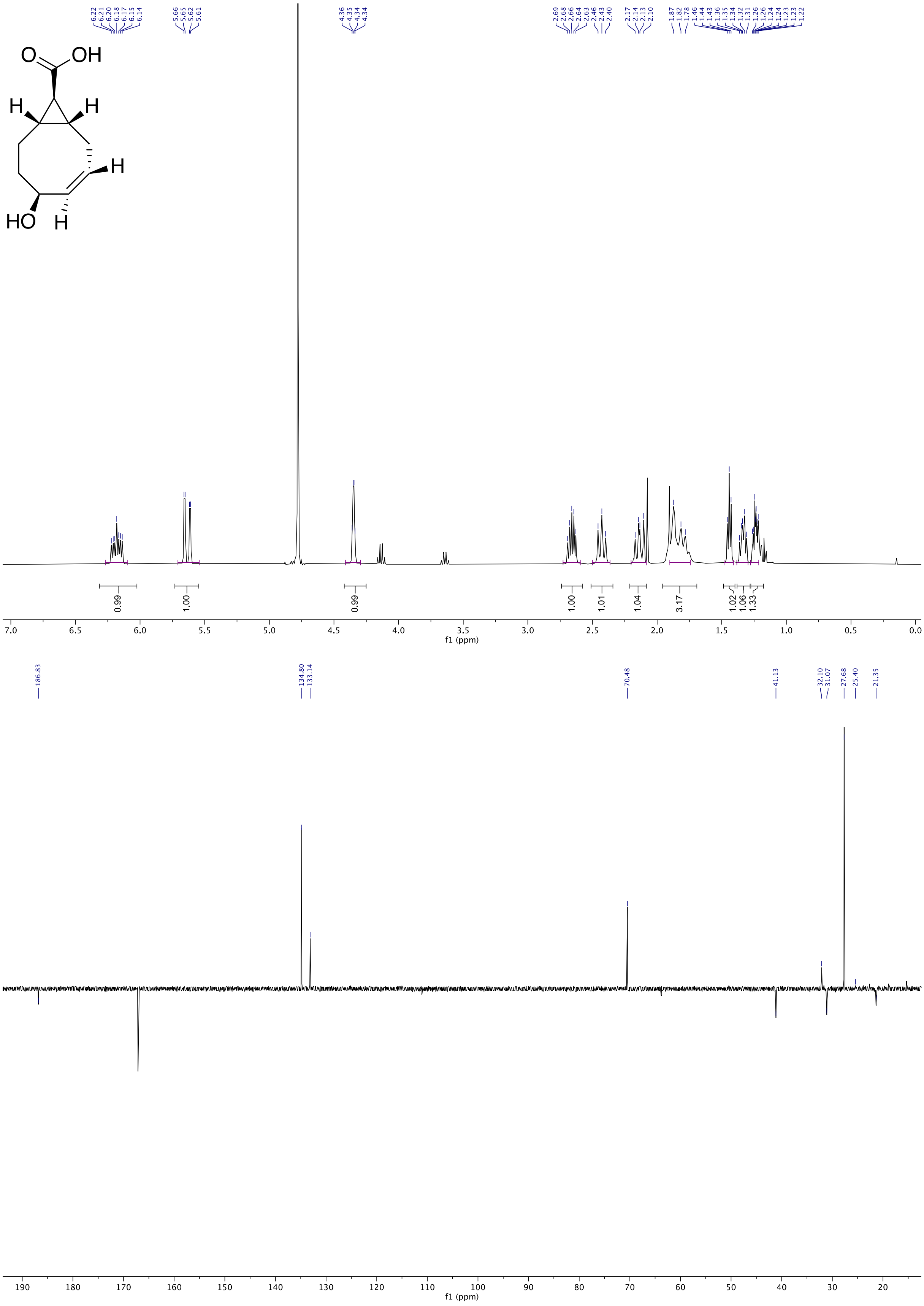

Figure S43. ^{1}H and ^{13}C NMR spectrum of compound **29**.

7. References

[1] J. Dommerholt, S. Schmidt, R. Temming, L. J. A. Hendriks, F. P. J. T. Rutjes, J. C. M. van Hest, D. J. Lefeber, P. Friedl, F. L. van Delft, *Angew. Chem. Int. Ed.* **2010**, *49*, 9422–9425.

[2] D. Blanco-Ania, L. Maartense, F. P. J. T. Rutjes, *ChemPhotoChem* **2018**, *2*, 898–905.

[3] R. M. Versteegen, R. Rossin, W. ten Hoeve, H. M. Janssen, M. S. Robillard, *Angew. Chem. Int. Ed.* **2013**, *52*, 14112–14116.

[4] R. M. Versteegen, W. Ten Hoeve, R. Rossin, M. A. R. de Geus, H. M. Janssen, M. S. Robillard, *Angew. Chem. Int. Ed.* **2018**, *57*, 10494–10499.

[5] X. Fan, Y. Ge, F. Lin, Y. Yang, G. Zhang, W. S. C. Ngai, Z. Lin, S. Zheng, J. Wang, J. Zhao, J. Li, P. R. Chen, *Angew. Chem. Int. Ed.* **2016**, *55*, 14046–14050.
